# An epigenetic barrier sets the timing of human neuronal maturation

**DOI:** 10.1038/s41586-023-06984-8

**Published:** 2024-01-31

**Authors:** Gabriele Ciceri, Arianna Baggiolini, Hyein S. Cho, Meghana Kshirsagar, Silvia Benito-Kwiecinski, Ryan M. Walsh, Kelly A. Aromolaran, Alberto J. Gonzalez-Hernandez, Hermany Munguba, So Yeon Koo, Nan Xu, Kaylin J. Sevilla, Peter A. Goldstein, Joshua Levitz, Christina S. Leslie, Richard P. Koche, Lorenz Studer

**Affiliations:** 1 The Center for Stem Cell Biology and Developmental Biology Program, Memorial Sloan Kettering Cancer Center, New York, NY USA; 2 Computational Biology Program, Memorial Sloan Kettering Cancer Center, New York, NY USA; 3 Department of Anesthesiology, Weill Cornell Medicine, New York, NY USA; 4 Department of Biochemistry, Weill Cornell Medicine, New York, NY USA; 5 Weill Cornell Neuroscience PhD Program, New York, NY USA; 6 Louis V. Gerstner Jr Graduate School of Biomedical Sciences, Memorial Sloan Kettering Cancer Center, New York, NY USA; 7 Center for Epigenetics Research, Memorial Sloan Kettering Cancer Center, New York, NY USA; 8 Present Address: Institute of Oncology Research (IOR), Bellinzona Institutes of Science (BIOS+), Bellinzona, Switzerland; 9 Present Address: Faculty of Biomedical Sciences, Università della Svizzera Italiana, Lugano, Switzerland; 10 Present Address: Microsoft AI for Good Research, Redmond, WA USA

**Keywords:** Stem-cell differentiation, Stem-cell biotechnology, Neural stem cells, Neuronal development

## Abstract

The pace of human brain development is highly protracted compared with most other species^1–7^. The maturation of cortical neurons is particularly slow, taking months to years to develop adult functions^3–5^. Remarkably, such protracted timing is retained in cortical neurons derived from human pluripotent stem cells (hPSCs) during in vitro differentiation or upon transplantation into the mouse brain^4,8,9^. Those findings suggest the presence of a cell-intrinsic clock setting the pace of neuronal maturation, although the molecular nature of this clock remains unknown. Here we identify an epigenetic developmental programme that sets the timing of human neuronal maturation. First, we developed a hPSC-based approach to synchronize the birth of cortical neurons in vitro which enabled us to define an atlas of morphological, functional and molecular maturation. We observed a slow unfolding of maturation programmes, limited by the retention of specific epigenetic factors. Loss of function of several of those factors in cortical neurons enables precocious maturation. Transient inhibition of EZH2, EHMT1 and EHMT2 or DOT1L, at progenitor stage primes newly born neurons to rapidly acquire mature properties upon differentiation. Thus our findings reveal that the rate at which human neurons mature is set well before neurogenesis through the establishment of an epigenetic barrier in progenitor cells. Mechanistically, this barrier holds transcriptional maturation programmes in a poised state that is gradually released to ensure the prolonged timeline of human cortical neuron maturation.

## Abstract (web summary)

The slow maturation of human neurons is regulated by epigenetic modification in nascent neurons, mediated by EZH2, EHMT1, EHMT2 and DOT1L.

## Main

The development of the central nervous system follows a coordinated sequence of events in which diverse cell identities are specified, differentiated and assembled into mature circuits. Although fundamental developmental steps are broadly conserved throughout mammalian evolution, the pace of development is protracted in humans compared with rodents or even primates^1–4^. The in vivo sequential order, duration and pace of developmental transitions are largely maintained ex vivo, such as during in vitro pluripotent stem cell (PSC) differentiation^10^. For instance, PSCs from various species, differentiated towards cerebral cortex lineages, faithfully recapitulate the sequential generation of neuron subtypes and glia, following a ‘schedule’ that largely matches the species-specific pace of in vivo cortical development^8,11–13^. The neurogenic capacity of neural stem cells and the sequential specification of cortical cell types is orchestrated by specific transcriptional and epigenetic pathways^14–17^. Temporal variations of neurogenesis and cell-fate specification during evolution have been linked to the increased human brain size and complexity^5^. However, the mechanisms that direct the timing of neuronal maturation after the establishment of neuronal fate remain largely unexplored. The pace of mouse versus human neuronal maturation shows a marked (approximately tenfold) timing difference compared to the two to threefold difference in the rate of early embryogenesis^8,9,13,18–21^. Neuronal maturation can last weeks, months or even years, depending on the species^6^. One prominent example is the human cerebral cortex, where synaptogenesis and neural circuit maturation continue for years into late postnatal life^6,7^. Similarly, hPSC-derived cortical neurons require months to reach mature electrophysiological and synaptic function^9,13,22–25^.

Extrinsic signals, including neuron–glia interactions^26,27^, network activity^28^ and secreted molecules^29^ can modulate aspects of neuronal morphogenesis, excitability and connectivity. However, compelling evidence indicates that the pace of neuronal maturation follows a cell-intrinsic programme. hPSC-derived cortical neurons transplanted into the rapidly maturing mouse neocortex develop adult-like morphologies, connectivity and dendritic spine function after nine months, compared with the four weeks required for mouse PSC-derived cortical neurons^9,30^. Furthermore, grafting human cortical neurons into mouse stroke models or human dopaminergic neurons into a rat model of Parkinson’s disease takes more than five months to induce functional recovery^24,31^. Accordingly, transplanted human neurons follow a species-specific timing of maturation rather than adopting the pace of the host species. Such protracted maturation timing poses a major challenge for studying neurological disorders that involve altered functionality of postnatal neural networks^32^. Thus, understanding the mechanisms that drive maturation timing is critical for accessing the full potential of hPSC technologies for modelling and treating brain disorders.

## hPSC-based model of neuronal maturation

**Fig. 1:**
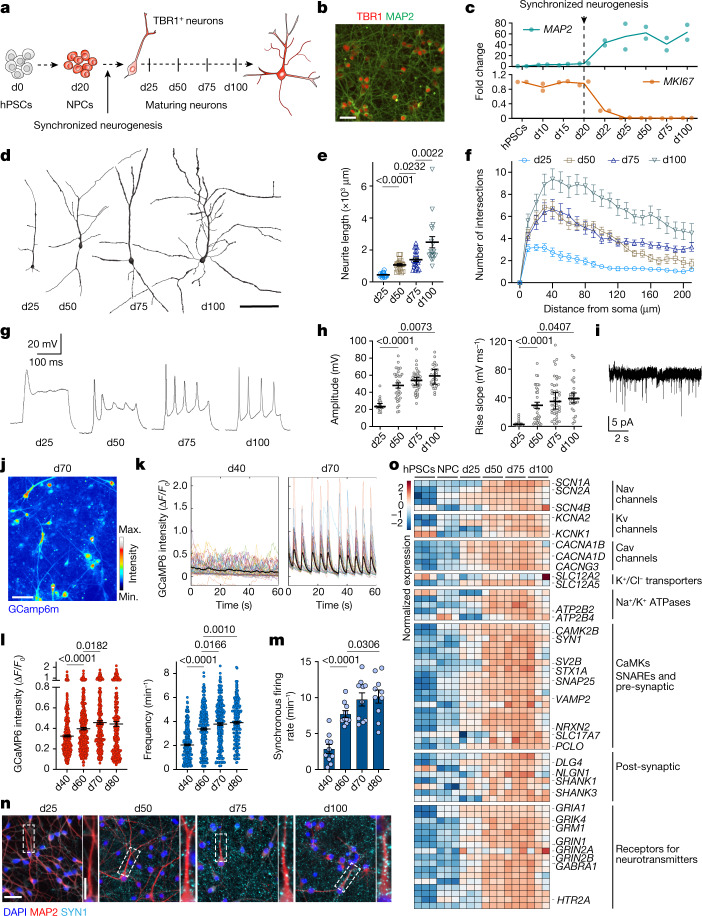
Morphological and functional maturation of synchronized cortical neurons derived from hPSCs. **a**, Experimental paradigm. hPSC-derived cortical NPCs were induced for synchronized neurogenesis at d20, and neurons were analysed at d25, d50, d75 and d100. **b**, Representative images of neuronal cultures stained for TBR1. **c**, Expression of *MKI67* and *MAP2* throughout the differentiation by quantitative PCR with reverse transcription (RT–qPCR). *n* = 2 independent experiments. **d**–**f**, Representative reconstructions of neuronal morphologies (**d**) and quantification of neurite length (**e**) and complexity (Sholl analysis) (**f**). d25: *n* = 16; d50: *n* = 20; d75: *n* = 23; d100: *n* = 18 (neurons from 2 independent experiments). **g**, Representative traces of electrophysiological recordings of evoked action potentials. **h**, Quantification of electrophysiological measurement of action potential amplitude and rise slope in neurons over time. d25: *n* = 25; d50: *n* = 33; d75: *n* = 43; d100: *n* = 29 (neurons from 10 independent experiments). **i**, Representative trace of mEPSCs at d75. **j**, Representative maximal intensity projection of Ca^2+^ imaging at d70. **k**, Representative traces of normalized GCaMP6m intensity in d40 and d70 neurons **l**, Quantification of amplitude and frequency of spontaneous Ca^2+^ spikes. d40: *n* = 395; d60: *n* = 418; d70: *n* = 299; d80: *n* = 239 (neurons from 2 independent experiment). **m**, Synchronous firing rate per min of imaging. *n* = 10 fields of view (FOV) per timepoint from 2 independent experiments. **n**, Representative images of SYNI and MAP2 staining in maturing neurons. Regions highlighted in the main image are enlarged on the right. **o**, Heat map for the normalized expression of selected transcripts important for neuronal functionality by RNA-seq. *n* = 3 independent experiments. CaMK, Ca^2+^/calmodulin-dependent protein kinase. Data are mean ± s.e.m. Scale bars: 50 μm (**b**); 100 μm (**d**,**j**); 50 μm (**n**) and 20 μm (**n**, enlarged view). Two-tailed unpaired *t*-test (**e**,**h**,**m**). Welch’s one-way ANOVA with Games–Howell’s correction (**l**). Source Data

**Extended Data Fig. 1:**
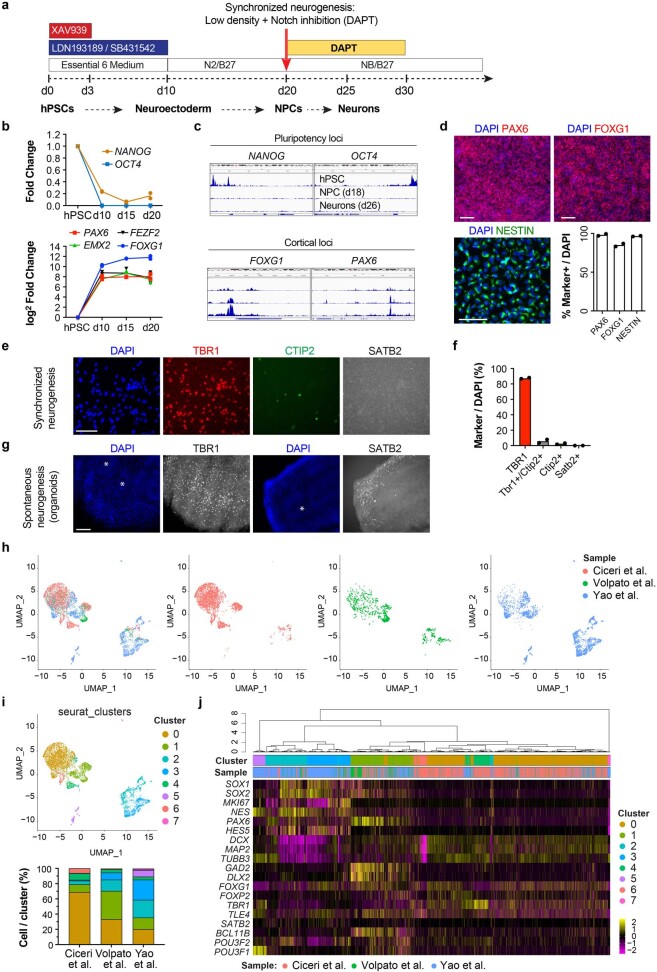
A novel platform for the synchronized generation of cortical neurons from hPSCs. **a**, Schematics of differentiation protocol based on dual-SMAD and WNT inhibition. Top panel indicate differentiation days, basal media, and small molecules treatments. Bottom panel indicate cell stages/types found at transition points. The red arrow indicates cell-passaging at low density in presence of notch pathway inhibitor DAPT. **b**, Expression of pluripotency (top) and cortical (bottom) specific markers by RT-qPCR throughout the differentiation (n = 2 independent experiments). **c**, Representative Genome browser traces of ATACseq peaks at hPSC, NPC and neuron stages in Pluripotency (*NANOG, OCT4*) and cortical (*PAX6, FOXG1*) loci (n = 2 independent experiments). **d**, Representative images and quantification of the fraction of cells expressing PAX6, FOXG1 and NESTIN cortical NPC markers at d20 of differentiation (n = 2 independent experiments). **e**–**f**, Representative images (e) and quantification (f) of the fraction of neurons generated through synchronized neurogenesis and expressing TBR1, CTIP2 and SATB2 cortical neuron markers (n = 2 independent experiments). **g**, Representative images of neurons generated through spontaneous neurogenesis (cortical organoids) and stained with antibodies against cortical neurons markers (n = 2 independent experiments). * Marks rosette structures of neural precursor cells. **h**, Single-cell RNAsequencing (scRNA-seq) experiments showing UMAP plots for the integrated dataset from the present study, Volpato et al., and Yao et at. **i**, Seurat clusters (top) and bar plot (bottom) for percentage of cells in each cluster across datasets. **h**, Hierarchical clustering of integrated scRNA-seq showing the expression of indicated markers across samples of origin and Seurat clusters. Colors for samples and clusters match those of (h) and (i). Histograms depict mean ± s.e.m. Scale bars are 50 μm (e); 100 μm (d) and 200 μm (g). Source Data

**Extended Data Fig. 2:**
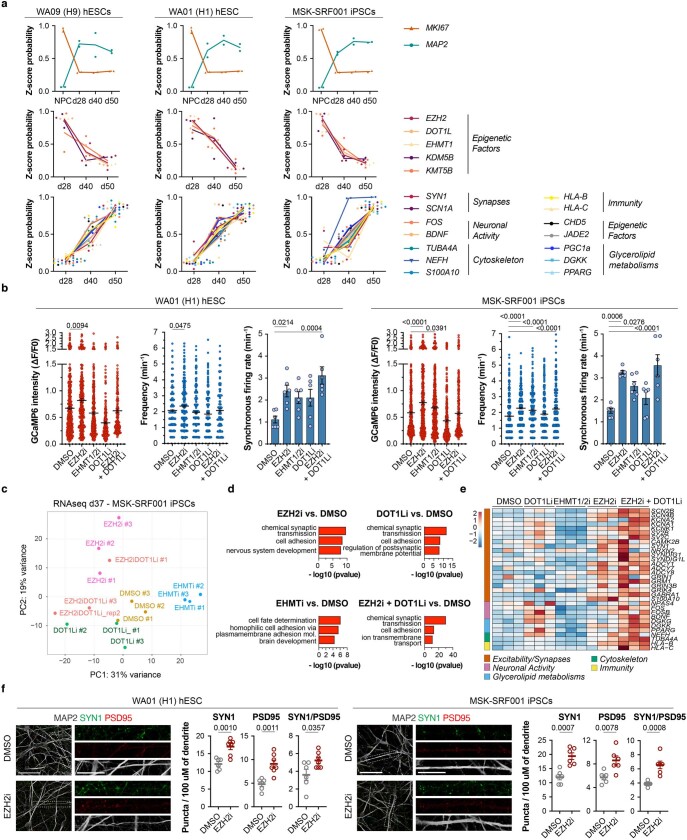
Validation of maturation signatures and paradigms of epigenetic inhibition in neurons derived from additional hPSC and iPSC lines. **a**, RT-qPCR expression analysis of NPC and neuron markers (top), selected epigenetic factors (middle) and maturation markers capturing several maturation phenotypes (bottom), upon synchronized differentiation of WA09 and WA01 hPSC and MSK-SRF001 iPSC. Data are represented as z-score normalized expression (n = 2 independent experiments per cell line). **b**, Quantification of amplitude and frequency of spontaneous Ca^2+^ spikes and synchronous firing rate in neurons derived from treated versus DMSO control NPCs of WA01 hESC and MSK-SRF001 iPSC. Amplitude and frequency, WA01: DMSO n = 352, EZH2i n = 313, EHMT1/2i n = 324, DOT1Li n = 310, EZH2i + DOT1Li n = 184; MSK-SRF001: DMSO n = 423, EZH2i n = 372, EHMT1/2i n = 485, DOT1Li n = 362, EZH2i + DOT1Li n = 315 (neurons from 2 independent experiments). Synchronous firing rate (n = 6 FOV from 2 independent experiments). **c**–**e**, RNA-seq studies in d37 neurons derived from MSK-SRF001 iPSC upon NPC treatments versus DMSO control conditions. PCA plot for RNA-seq datasets show samples distribution according to treatments (c). GO for upregulated transcripts (d). Heatmap for the normalized expression of representative transcripts within selected bivalent genes in treated and control conditions (e). n = 3 independent experiments. **f**, Representative images and quantification of the number of SYN1 and PSD95 synaptic puncta in d65 neurons derived from EZH2i versus DMSO conditions from WA01 and MSK-SRF001 lines (n = 6 FOV from 2 independent experiments per each line). Data is mean ± s.e.m. Welch’s one-way ANOVA with Games-Howell’s multiple comparisons test (amplitude and frequency in b); ordinary one-way ANOVA with Dunnett correction (synchronicity in b); Fisher’s Exact test (d); two-tailed unpaired t-test (f). Scale bars in (f) are 50 μm and 25 μm (insets). FOV, field of view. Source Data

A challenge in using PSC-based models to study human neuronal maturation is the poor temporal synchronization and cell heterogeneity during differentiation. Typically, multiple neuronal lineages coexist with neuronal precursor cells (NPCs), which generate newly born neurons that each may differentiate at a heterochronic pace. Here we developed a novel hPSC-based platform that generates homogeneous populations of cortical neurons in a temporally synchronized manner (Fig. 1a). We induced central nervous system neuroectoderm from hPSCs by dual SMAD inhibition^25^ and WNT signalling inhibition^22^ (Extended Data Fig. 1a), which efficiently pattern NPCs expressing the cortical specific markers *FOXG1*, *PAX6*, *EMX2* and *FEZF2* by day of differentiation (d)10 (Extended Data Fig. 1b), as further shown by stage-dependent accessibility changes at pluripotency versus forebrain chromatin loci (Extended Data Fig. 1c). Our platform yields a homogeneous population of cortical NPCs by d20 (Extended Data Fig. 1d), which can be triggered towards synchronous neurogenesis via optimized density of cell replating and treatment with DAPT, an inhibitor of Notch signalling, a pathway that is critical for NPC maintenance^33,34^ (Extended Data Fig. 1a and Supplementary Fig. 2a). By d25, nearly all Ki67^+^ NPCs have exited the cell cycle and turned into roughly isochronic MAP2^+^ post-mitotic neurons (Supplementary Fig. 2b,c), as confirmed by EdU birth-dating (Supplementary Fig. 2d,e) and validated in independent hPSC and induced pluripotent stem cell (iPSC) lines (Extended Data Fig. 2a). Synchronized neurons can be maintained for more than 100 days with no additional neurogenesis occurring after d25 (Fig. 1c and Supplementary Fig. 2f). Single-cell RNA-sequencing (scRNA-seq) studies at d27 confirmed rapid and efficient NPC depletion and generation of post-mitotic neurons compared with lengthier, non-synchronized hPSC cortical differentiation protocols (Extended Data Fig. 1h–j).

There is a strong correlation between birth date and identity of cortical neurons^11,12,35^. Accordingly, synchronized neurogenesis generated nearly pure early born, lower layer TBR1^+^ neurons (Fig. 1b and Extended Data Fig. 1e,f). This contrasts with other cortical differentiation systems in which neurogenesis occurs spontaneously throughout differentiation, generating multiple neuronal identities that coexist with NPCs (for example, TBR1- and SATB2-expressing neurons in cortical organoids; Extended Data Fig. 1g). scRNA-seq analysis confirmed lower layer excitatory neuron identity with only a minor population of GABAergic (γ-aminobutyric acid-producing) cells (Extended Data Fig. 1h–j). Thus, induction of synchronized neurogenesis provides an excellent platform for tracing the intrinsic maturation of roughly coetaneous human cortical neurons over months in vitro.

## hPSC-derived neurons mature gradually

We first tracked the morphometric and functional development of neurons at d25, d50, d75 and d100 (Fig. 1d). We observed a significant increase in the total length and complexity of neurite arborizations over time (Fig. 1d–f), with progressively more mature electrophysiological properties. Newly born neurons at d25 exhibited abortive or low-amplitude evoked single action potentials and gradually acquired more mature intrinsic functionality, including hyperpolarized membrane potential, decreased input resistance, repetitive evoked action potentials with increased amplitude and faster kinetics (Fig. 1g,h and Supplementary Table 1), and progressively developed functional excitatory synapses, as evidenced by the presence of miniature excitatory postsynaptic currents (mEPSCs) (Fig. 1i). Calcium imaging using GCaMP6m (Fig. 1j,k) confirmed a marked but gradual increase in amplitude and frequency of spontaneous Ca^2+^ spikes (Fig. 1l) and revealed a sparse-to-synchronous firing switch in network activity by d60 (Fig. 1k,m and Supplementary Videos 1 and 2).

The gradual onset of functional properties correlated with increased localization of SYN1 in presynaptic puncta-like structures (Fig. 1n) and with the concerted expression, shown by RNA sequencing (RNA-seq) at d25, d50, d75 and d100, of genes related to neuronal excitability (voltage-gated ion channels) and connectivity (pre- and postsynaptic compartments and receptors for neurotransmitters), including those encoding cation/chloride transporters (*SLC12A5* and *SLC12A2* (also known as *KCC2* and *NKCC1*)) that modulate the excitatory-to-inhibitory developmental GABA switch^36^ and glutamate receptor subunits that are known to correlate with neuronal maturity^37^ (*GRIN2B* and *GRIN2A*) (Fig. 1o).

**Fig. 2:**
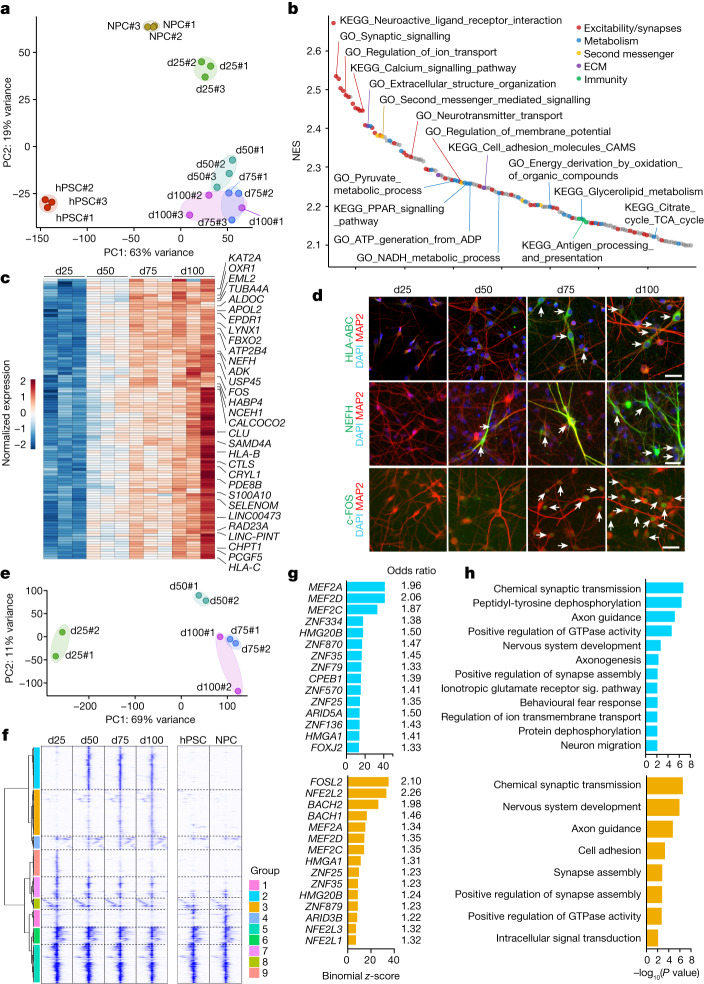
Molecular staging of neuronal maturation. **a**, PCA plot of RNA-seq datasets show distribution of samples according to their time of differentiation based on top 1000 differentially expressed transcripts with variance stabilized normalization. **b**, Waterfall plot of top 150 pathways that are enriched in more mature neurons by GSEA in d50 versus d25 comparison. NES, normalized enrichment score. **c**, Heat map for the normalized temporal expression of strict monotonically upregulated transcripts (maximum log fold change (FC) >1 at any comparison, expression > 5 reads per kilobase per million mapped reads (RPKM) at any timepoint, s.e.m. at d100 < 1). **d**, Representative images of neurons at indicated timepoints, stained with antibodies for indicated maturation markers. **e**, PCA plot of ATAC-seq dataset shows distribution of samples according to their maturation stage. **f**, Agglomerative hierarchical clustering by Ward linkage of differentially accessible ATAC-seq peaks in neurons identifies nine groups of peaks with stage-specific accessibility. **g**, Top 15 enriched transcription factor binding motifs at late-opening ATAC-seq peaks by the hypergeometric test (top, group 2; bottom, group 3). Odds ratio indicates the normalized enrichment of transcription factor motifs in the cluster compared to the background. **h**, GO term analysis for genes linked at late-opening ATAC-seq group 2 (top) and 3 (bottom) peaks show enrichment for synaptic-related pathways. Fisher’s Exact test with Benjamini correction. Sig., signalling. RNA-seq and downstream analyses: *n* = 3 independent experiments; ATAC-seq and downstream analyses: *n* = 2 independent experiments. Source Data

Principal component analysis (PCA) of RNA-seq data showed a sample distribution according to developmental stage, with hPSC-to-NPC and NPC-to-neurons representing the most distant transitions. In neurons, pronounced changes occurred between d25 and d50, followed by more subtle differences between d50, d75 and d100 (Fig. 2a). On the basis of PCA, we inferred d25, d50 and d100 as three discrete maturation stages. Gene set enrichment analysis (GSEA) in d50 versus d25 revealed significant enrichment for neuronal excitability and synapse-related gene ontology (GO) terms. Energy and glycerolipid metabolism and PPAR signalling were also enriched, linking metabolism to neuronal maturation^38,39^. We additionally observed enrichment for immunity-related GO terms such as antigen processing and presentation (Fig. 2b). Similar GO terms were enriched between d100 versus d50 (Supplementary Fig. 3a), further supporting a gradual unfolding of the maturation signature. We therefore unbiasedly selected differentially expressed transcripts showing monotonic upregulation (Fig. 2c, Supplementary Fig. 3b and Methods), which efficiently captured multiple maturation phenotypes, including cytoskeleton (*TUBA4A* and *NEFH*), Ca^2+^ signalling and homeostasis (*ATP2B4*), ATP biosynthesis (*ALDOC*), lipid and cholesterol metabolism (*APOL2* and *NCEH1*), protein biosynthesis and degradation (*AARS*, *FBXO2* and *USP45*), antioxidant responses (*OXR1*), immunological (*HLA-B* and *HLA-C*) and activity-dependent changes (*FOS* and *LINC00473*) (Fig. 2c). We validated the stage-specific expression of selected maturation markers (*HLA-ABC*, *NEFH* and *FOS)* by immunofluorescence (Fig. 2d) and by RT–qPCR in neurons derived from additional hPSC and iPSC lines (Extended Data Fig. 2a). The progressive upregulation of specific transcripts largely matched trends of in vivo gene expression based on BrainSpan^40^, with changes occurring late perinatally or early postnatally (Supplementary Fig. 3c).

We next assessed chromatin accessibility changes by assay for transposase-accessible chromatin with sequencing (ATAC-seq) at d25, d50, d75 and d100, which, similar to RNA-seq datasets, showed sample alignment along the maturation timeline by PCA (Fig. 2e) with many peaks changing accessibility between d25 and d50 followed by more subtle changes afterwards (Supplementary Fig. 4a). To uncover maturation-dependent chromatin dynamics, we compiled an atlas of around 20,000 ATAC-seq peaks that included all differentially accessible peaks across pairwise comparisons of d25, d50, d75 and d100 samples. Unbiased clustering of these peaks outlined nine groups (Fig. 2f). Groups 5, 6 and 8 mapped primarily at gene promoters. All other ATAC-seq groups mapped primarily at putative enhancers following two basic, maturation-dependent patterns (Fig. 2f and Supplementary Fig. 4b). Groups 1 and 9 defined peaks with increased accessibility in immature neurons (Fig. 2f). Conversely, peaks in groups 2, 3 and 4 progressively gained accessibility during maturation. Inference of group-specific upstream regulators via transcription factor motif analysis revealed that peaks that were highly accessible in young neurons were enriched for motifs that are important during early cortical development^41^, including *OTX2*, *SOX4*, *EMX2*, *LHX2*, *POU3F1* and *POU3F2* (Supplementary Fig. 4c). Group 2 and 3 peaks showed instead high enrichment for activity-dependent transcription factor motifs^28,42,43^, including *MEF2* and *AP-1* complex members (Fig. 2g), as further confirmed by d50 versus d25 and d100 versus d50 comparisons (Supplementary Fig. 4d). Notably, gain in accessibility at peaks associated with activity-dependent transcription factors was correlated with their increased expression (for example, in Fig. 2c,d) and coincides with the onset of synchronous network activity (Fig. 1j–m). Furthermore, genes linked to the late-opening group 2 and 3 peaks were enriched for synapse-related GO terms (Fig. 2h).

## Epigenetic switch of neuronal maturation

**Fig. 3:**
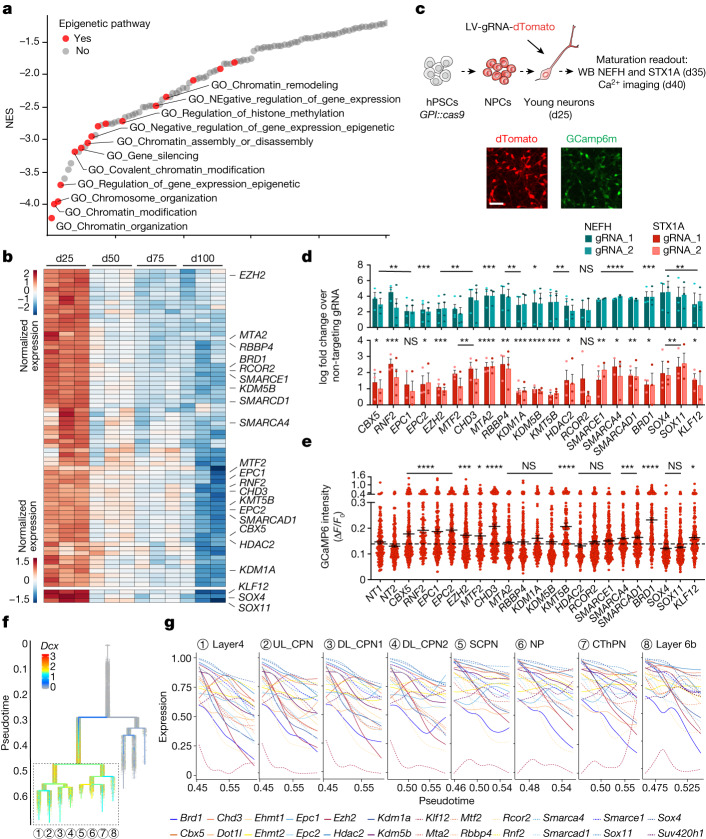
An epigenetic switch drives neuronal maturation. **a**, Waterfall plot of the top 100 pathways that are negatively correlated with neuronal maturation by GSEA in d50 versus d25 comparison of RNA-seq samples. *n* = 3 independent experiments. Red dots indicate epigenetic pathways. **b**, Heat map for normalized expression of monotonically downregulated chromatin regulators during maturation (with maximum logFC > 1 at any comparison). Top, epigenetic factors; bottom, transcription factors. *n* = 3 independent experiments. Labelled genes were selected for gene knockout studies. **c**, Experimental paradigm for gene knockout in cortical neurons derived from hPSCs: Cas9 expressing neurons at d25 were infected with lentiviruses encoding gene-specific gRNAs. Induction of precocious maturation was assessed by western blot (WB) at d35 and Ca^2+^ imaging at d40. Scale bar, 50 μm. **d**, Normalized expression by western blot of the maturation markers NEFH and STX1A upon gene knockout in neurons (two gRNAs per gene). Histograms depict average log_2_FC ± s.e.m. over non-targeting gRNA samples. Dots represent independent experiments. **e**, Amplitude of spontaneous Ca^2+^ spikes of individual neurons upon gene knockout. The dotted line represents the average amplitude for the two non-targeting gRNA. Dots represent individual neurons from two independent experiments. **f**, Branching lineage tree from scRNA-seq data of mouse development (Di Bella et al.^41^) showing *Dcx* expression. **g**, Temporal expression of mouse chromatin regulator genes homologous to the human genes that are perturbed in hPSC-derived neurons (**d**) across multiple neuron subtypes in the mouse neocortex. UP, upper layer; DL, lower layer; CPN, callosal projection neurons; SCPN, subcerebral projection neurons; NP, near projecting; CThPN, cortico-thalamic projection neurons. ****P < 0.0001, ***P < 0.001, **P < 0.01, *P < 0.05. NS, not significant. *n* and *P* values in **d** and **e** are reported in Supplementary Tables 2 and 3. Two-tailed one sample *t*-test (**d**); Welch’s one-way ANOVA with Games–Howell’s multiple comparisons test (**e**). Source Data

We next sought to identify mechanisms responsible for the protracted maturation of hPSC-derived neurons. When focusing on genes downregulated during maturation, chromatin organization and epigenetic-related pathways emerged as the most significantly regulated categories by GSEA, for both d50 versus d25 and d100 versus d50 comparisons (Fig. 3a and Supplementary Fig. 5a,c,d). Focusing on epigenetic factors, we identified a core set of transcripts whose levels monotonically decreased during maturation (Fig. 3b), recapitulating expression dynamics observed in the human cortex in vivo (Supplementary Fig. 5b). Monotonically downregulated chromatin regulator genes encode members of several complexes including Polycomb repressive complex 1 and 2 (PRC1/2), BAF, MOZ and MORF, NuRD, and histone lysine demethylases and methyltransferases, a finding confirmed in additional hPSC and iPSC lines (Extended Data Fig. 2a). To test whether the retention of these epigenetic pathways limits neuronal progression towards maturity, we performed CRISPR–Cas9 loss-of-function studies for 21 genes comprising 18 chromatin regulators and 3 transcription factors (*SOX4*, *SOX11* and *KLF12*) that exhibited monotonic downregulation (Fig. 3b,c). We transduced synchronized post-mitotic neurons expressing Cas9 at d25 (GPI::Cas9 hPSC line; Supplementary Fig. 6 and Methods) with an arrayed lentiviral library encoding dTomato and gene-specific guide RNAs (gRNAs) (two gRNAs per gene and 2 non-targeting control gRNAs). We screened each perturbation for the ability to trigger precocious expression of the cytoskeleton NEFH and presynaptic STX1A maturation markers (Supplementary Fig. 7b) by western blot at d35 and by performing Ca^2+^ imaging at d40 (Fig. 3c). Western blot analysis revealed increased NEFH and STX1A expression across many gene perturbations relative to non-targeting gRNAs (Fig. 3d and Supplementary Fig. 7c). Furthermore, loss of function of 12 out of 21 factors induced significantly increased amplitudes of spontaneous Ca^2+^ spikes (Fig. 3e) and synchronous firing rates (Supplementary Fig. 7d). Those chromatin regulators comprise genes related to PRC1/2 (*CBX2*, *RNF2*, *EPC1*, *EPC2I*, *EZH2I* and *MTF2*), NuRD (*CHD3*), lysine methyltransferases (*KMT5B*), BAF (*SMARCA4* and *SMARCAD1*) and bromodomain-containing complexes (*BRD1*). Although our study does not rule out the possibility that other pathways (Fig. 2b and Supplementary Figs. 3a,b and 5c,d and) or additional epigenetic factors (Fig. 3b) further regulate the maturation process, we identified several chromatin regulators whose loss of function robustly triggers neuronal maturation. Notably, this epigenetic signature is similarly downregulated across multiple subtypes of maturing neurons in the mouse cortex in vivo (Fig. 3f,g), albeit at a faster pace than in human cells^44^. Together, these results suggest the existence of an epigenetic ‘brake’ that is released gradually to ensure a protracted unfolding of neuronal maturation programmes.

## An epigenetic barrier in NPC sets maturation pace

**Fig. 4:**
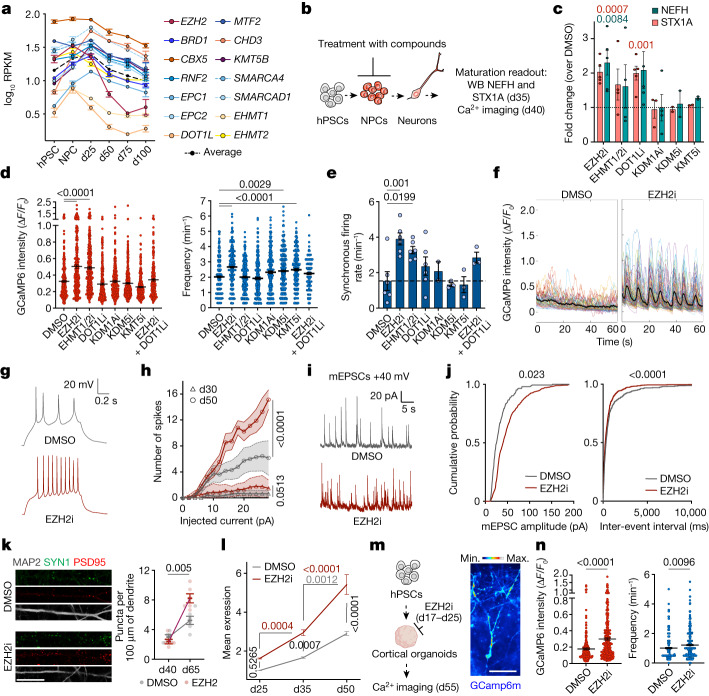
Transient inhibition of epigenetic factors in NPCs drives faster maturation in neurons. **a**, Temporal expression of epigenetic hits from gene knockout studies by RNA-seq (*n* = 3 independent experiments). **b**, Experimental paradigm. NPCs were treated transiently (d12 to d20) with epigenetic inhibitors. Neuronal maturation was assessed by western blot and Ca^2+^ imaging. **c**, Normalized expression of NEFH and STX1A by western blot. EZH2i, DOT1Li: *n* = 5; EHMT1/2i: *n* = 4; KMT5i: *n* = 2; KDM1Ai (STX1A), *n* = 3; KDM1Ai (NEFH) *n* = 4 (independent experiments). **d**, Amplitude and frequency of spontaneous Ca^2+^ spikes. DMSO: *n* = 292; EZH2i: *n* = 317; EHMT1/2i: *n* = 331; DOT1Li: *n* = 309; KDM1Ai: *n* = 282; KDM5i: *n* = 326; KMT5i: *n* = 326; EZH2i + DOT1Li: *n* = 221 (neurons from 2 independent experiment) **e**, Synchronous firing rate. DMSO, EZH2i, EHMT1/2, DOT1Li: *n* = 6; KDM1A: *n* = 2; KDM5i, KMT5i: *n* = 3 (FOV from 2 independent experiments). **f**, Representative traces of normalized GCaMP6m signal. **g**,**h**, Representative traces at d50 (**g**) and number of evoked action potentials (**h**) per injected current. DMSO: d30, *n* = 7; d50, *n* = 11. EZH2i: d30, *n* = 8; d50, *n* = 12 (neurons). Error bands indicate s.e.m. **i**,**j**, Representative traces (**i**) and amplitude and frequency (**j**) of mEPSCs (+40 mV, d60–d75). DMSO: *n* = 5; EZH2i: *n* = 8 (neurons). **k**, Representative images and quantification of juxtaposed SYN1–PSD95 puncta. *n* = 6 FOV from 2 independent experiments. Scale bar, 25 μm. **l**, Normalized expression (to d25 DMSO) of ‘relaxed’ monotonic upregulated transcripts by RNA-seq in neurons from EZH2i and DMSO-treated NPCs. *n* = 3 independent experiments. **m**, Experimental paradigm for transient EZH2i in forebrain organoids and representative image of Ca^2+^ imaging at d55. Scale bar, 50 μm. **n**, Amplitude and frequency of spontaneous Ca^2+^ spikes in organoids. DMSO: *n* = 222; EZH2i: *n* = 297 (4–8 organoids per condition from 2 batches). Data are mean ± s.e.m. Two-tailed ratio-paired *t*-test (**c**); Welch’s one-way ANOVA with Games–Howell’s correction (**d**); one-way ANOVA with Dunnett’s correction (**e**); mixed-effect model with Tukey’s correction (**h**); Kolmogorov–Smirnov test (**j**); two-tailed unpaired *t*-test (**k**); two-way ANOVA with Šidák correction (**l**); two-tailed Welch’s test (**n**). Source Data

**Fig. 5:**
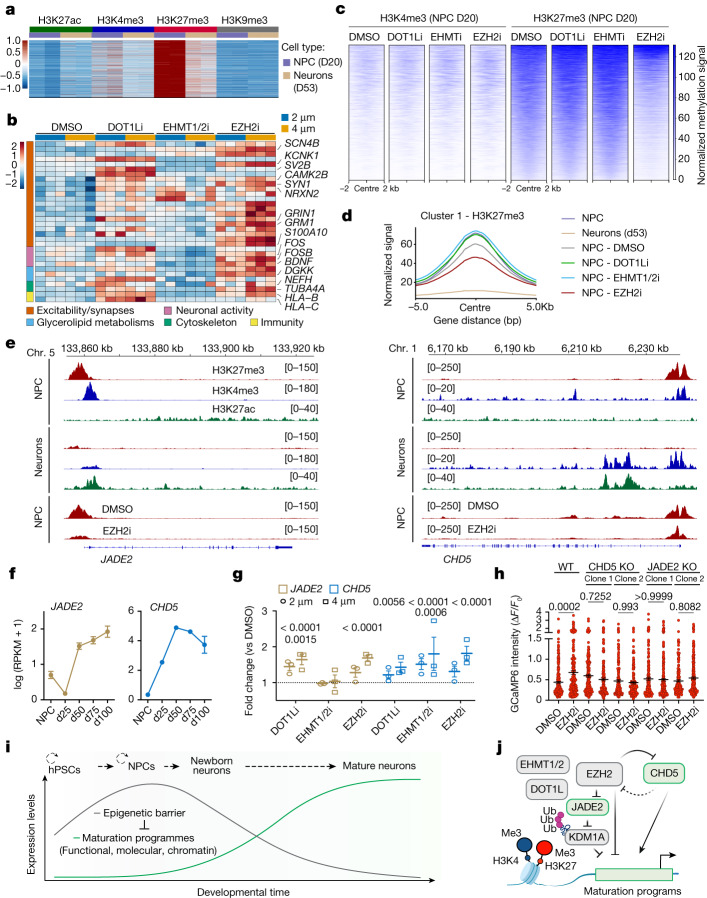
An epigenetic barrier in NPCs controls the onset of neuron maturation programmes. **a**, Heat map for cleavage under targets and release using nuclease (CUT&RUN) peaks with bivalent status in NPCs that get resolved towards active chromatin via H3K27me3 reduction in neurons (*n* = 2 independent experiments). Data are normalized signal from cluster 1 from CUT&RUN in Extended Data Fig. 10. **b**, Heat map for the normalized expression of representative bivalent genes by RNA-seq of d38 neurons derived from treated NPCs (2 and 4 μM) (*n* = 3 independent experiments). **c**, Tornado plots for the normalized H3K4me3 and H3K27me3 signals at bivalent peaks in NPCs upon epigenetic inhibition (*n* = 2 replicates per condition). **d**, Normalized H3K27me3 signal at bivalent peaks in NPCs upon epigenetic inhibition, untreated NPC and neurons. **e**, Representative tracks of H3K27me3, H3K4me3 and H3K27ac (untreated NPCs and neurons) and H3K27me3 (NPCs upon EZH2i and DMSO treatments) at *CHD5* and *JADE2* genomic loci. **f**,**g**, *CHD5* and *JADE2* expression by RNA-seq during maturation (**f**) and in d38 neurons derived from NPC upon epigenetic inhibition versus DMSO (2 and 4 μM) (**g**). *n* = 3 independent experiments. **h**, Amplitude of spontaneous Ca^2+^ spikes in neurons from EZH2i versus DMSO conditions derived from wild-type (WT), CHD5-knockout (KO) and JADE2-KO hPSC lines. DMSO: WT, *n* = 241; CHD5-KO clone 1, *n* = 184; CHD5-KO clone 2, *n* = 165; JADE2-KO clone 1, *n* = 183; JADE2-KO clone 2, *n* = 171 JADE2-KO clone 1,. EZH2i: WT, *n* = 190; CHD5-KO clone 1, *n* = 197; CHD5-KO clone 2, *n* = 147; JADE2-KO clone 1, *n* = 222; JADE2-KO clone 2, *n* = 213 (neurons from 2 independent experiments). **i**,**j**, Main conclusions of the study. **i**, The gradual unfolding of maturation signatures in hPSC-derived neurons is marked by the retention of an epigenetic barrier established at NPC stage. **j**, Key members of the epigenetic barrier maintain maturation programmes in a poised state through the deposition of repressive histone marks. Ub, ubiquitin. Data are mean ± s.e.m. Wald’s test with Benjamini–Hochberg correction (**g**); Welch’s one-way ANOVA with Games–Howell’s correction (**h**). Source Data

**Extended Data Fig. 3:**
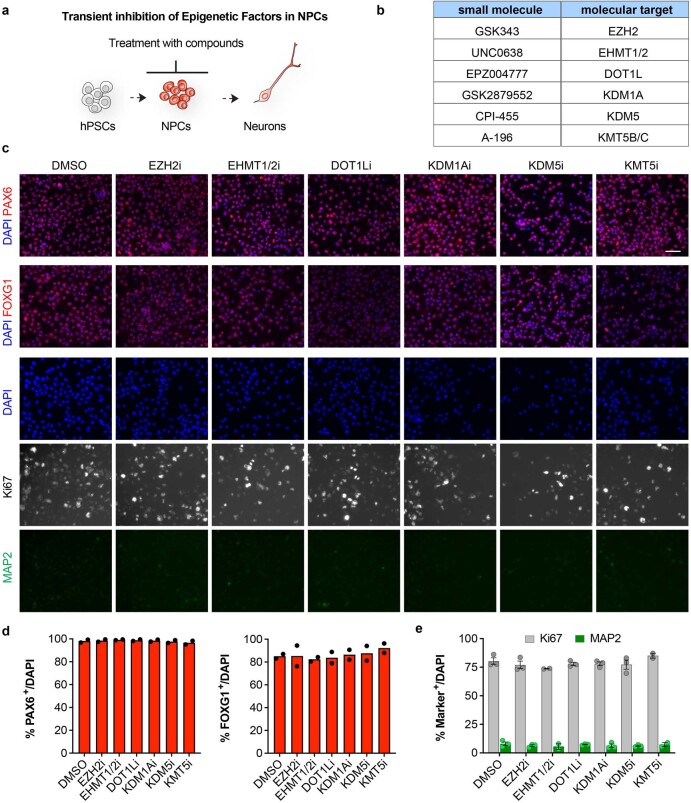
Transient inhibition of epigenetic factors in NPC does not alter cortical patterning and neurogenesis. **a**, Schematic of experimental paradigm for transient inhibition of chromatin regulators at progenitor cell stage. NPC were treated with small molecule from d12 to d20. **b**, Small molecule compounds used in the study and corresponding intracellular targets. **c**, Representative images of d20 NPCs treated with small molecule before the induction of synchronized neurogenesis and stained with antibodies against cortical markers PAX6 and FOXG1, the proliferation marker Ki67 and the neuron marker MAP2. **d**, Quantification of the fraction of cells expressing PAX6 and FOXG1 at d20 in treated versus control conditions (n = 2 independent experiments). **e**, Quantification of the fraction of progenitor cells (KI67 +) and neurons (MAP2 +) at d20 in treated versus control conditions. DMSO n = 3, EZH2i n = 3, EHMT1/2i n = 2, DOT1Li n = 3, KDM1Ai n = 3, KDM5i n = 3, KMT5i n = 2 (independent experiments). Histograms depict mean ± s.e.m. Scale bars are 50 μm. Source Data

**Extended Data Fig. 4:**
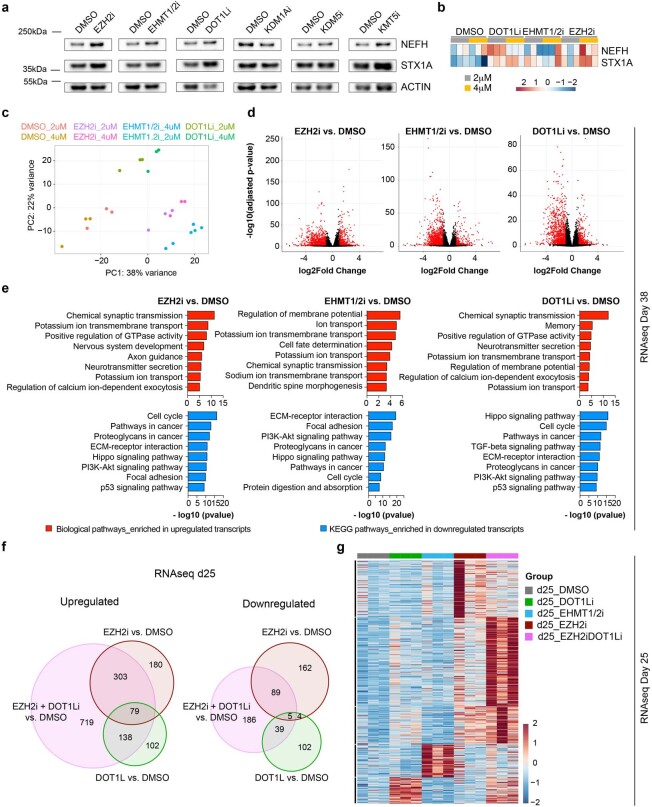
A small molecule mini screen identify EZH2, EHMT1/2 and DOT1L inhibition in NPC as a maturation driver in neurons. **a**, Representative western blots for the expression of NEFH and STX1A maturation markers in the transient inhibition of epigenetic factors in NPC experiments. NPC were treated with small molecule from d12 to d20 and neurons derived from each condition were analysed at d38. Quantification is shown in Fig. 4c. Gels/blots from the same experiments were processed in parallel. n = 2–5 independent experiments **b**, Heatmap for the expression of NEFH and STX1A by RNA-seq across treatments. **c**, PCA plot for RNA-seq dataset at d38 show samples distribution according to the pharmacological treatments (n = 3 independent experiments). **d**, Volcano plot for the indicated pairwise comparisons from RNA-seq studies (treatment with 4 μM of inhibitors). Red dots represent differentially expressed significant transcript (FDR 0.05) that show Fold Change >=2. **e**, GO for upregulated (top) and downregulated (bottom) transcripts from RNA-seq studies in pairwise comparisons (n = 3 independent experiments). **f**, Venn diagram for upregulated and downregulated transcripts by RNA-seq at d25 in neurons derived from transient treatment with EZH2i, DOT1Li and combined EZH2i + DOT1Li (n = 3 independent experiments). **g**, Heatmap showing k-means clustering for the normalized expression of “relaxed” monotonically upregulated transcripts that are differentially expressed in d25 neurons upon indicated treatments and combination (logFC > 1, FDR < 0.05). Wald’s test with Benjamini-Hochberg false discovery rate (d); Fisher’s Exact test (e). Source Data

Temporal expression analysis revealed that most epigenetic factors regulating neuronal maturation are expressed already in dividing NPCs (Fig. 4a), raising the possibility that those chromatin regulators may establish an epigenetic barrier during hPSC-to-NPC transition, prior to neuronal exit. We tested this hypothesis by applying pharmacological inhibition (i) of the histone lysine methyltransferases EZH2, KMT5B, EHMT1, EHMT2 and DOT1L and the demethylases KDM1A and KDM5 (Extended Data Fig. 3a,b) directly at the NPC stage. We treated NPCs transiently from d12 to d20, following cortical specification. The compounds were washed out and withdrawn at d20, before inducing synchronized neurogenesis. Neurons derived following epigenetic inhibition or DMSO treatment at the NPC stage were cultured under identical conditions and assessed for maturation by western blot at d35 and Ca^2+^ imaging at d40 (Fig. 4b). Neither treatment altered *PAX6* or *FOXG1* expression or induced precocious neurogenesis, based on NPC/neuron ratio at d20 (Extended Data Fig. 3c–e). Transient inhibition of EZH2, EHMT1/2 and DOT1L in NPCs using GSK343, UNC0638 and EPZ004777, respectively, induced increased NEFH and STX1A expression in d35 neurons (Fig. 4c and Extended Data Fig. 4a). Moreover, transient EZH2 inhibition triggered highly significant enhancement of all measured properties including amplitude, frequency and synchronicity of Ca^2+^ spikes relative to DMSO controls (Fig. 4d–f and Supplementary Videos 3 and 4). EHMT1/2 inhibition induced increased amplitude and synchronicity of Ca^2+^ spikes, whereas DOT1L inhibition showed a modest enhancement in synchronous firing rates (Fig. 4d–f and Supplementary Videos 3, 5 and 6). These results were recapitulated in neurons from additional hPSC and iPSC lines (Extended Data Fig. 2b). We next assessed global transcriptional changes in neurons generated from NPCs upon inhibition of EZH2, EHMT1/2 or DOT1L by RNA-seq at d38 (Extended Data Fig. 4c,d). Downregulated genes captured pathways typical of NPCs and newborn neurons (Extended Data Fig. 4e), including the SOX transcription factor and the Notch pathway. Consistent with enhanced neuronal functionality, upregulated transcripts were enriched for synaptic transmission and ion transmembrane transport GO terms (Extended Data Fig. 4e) as confirmed in iPSC-derived neurons (Extended Data Fig. 2c–e). These results demonstrate that enhanced neuronal maturation can be achieved through transient epigenetic inhibition at NPC stage and identified EZH2, EHMT1/2 and DOT1L as key components of the epigenetic barrier. Although each treatment enhanced transcriptional and functional maturation features, inhibition of EHMT1/2 had a more limited effect than inhibition of EZH2 or DOT1L (Fig. 5b and Extended Data Fig. 2e and 10f). We therefore focused on EZH2 and DOT1L to test whether combined inhibition might further enhance maturation. RNA-seq of d25 neurons revealed increased expression of a subset of maturation transcripts upon combined inhibition versus individual inhibition of EZH2 and DOT1L (Extended Data Fig. 4f,g). However, both amplitude and frequency of Ca^2+^ spikes at d40 upon combined EZH2 and DOT1L inhibition showed a smaller effect than inhibition of EZH2 alone, while triggering increased synchronous firing in neurons across different hPSC lines (Fig. 4d,e and Extended Data Fig. 2b). Given the limited benefit of combined treatment, we focused on transient EZH2 inhibition for in-depth electrophysiological studies.

**Extended Data Fig. 5:**
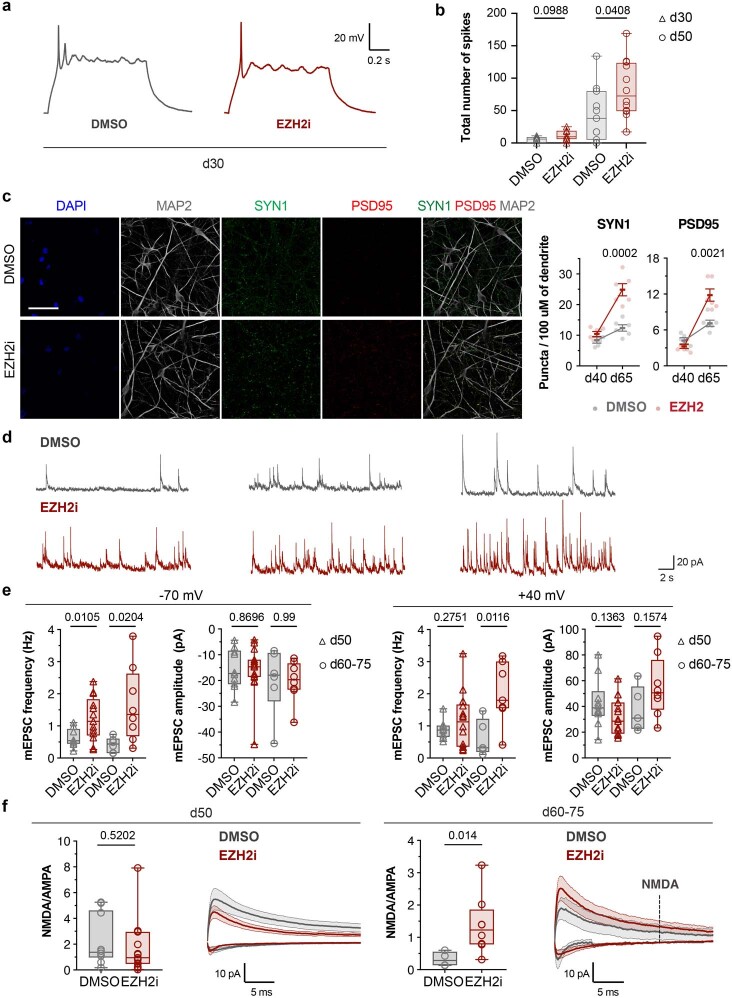
Transient EZH2i in NPCs triggers enhanced synaptic maturation in neurons. **a**, Representative traces of evoked action potentials at d30 in EZH2i versus DMSO conditions. **b**, Quantification of the total number of spikes in neurons at d30 and d50 from EZH2i versus DMSO conditions. DMSO: d30 n = 7, d50 n = 11; EZH2i: d30 n = 8, d50 n = 12. **c**, Representative images (d65) and quantification (d40 and d65) for the number of SYN1 and PSD95 puncta in neurons from DMSO and EZH2i groups (n = 6 FOV from 2 independent experiments). whiskers are mean ± s.e.m. Insets are shown in Fig. 4k. **d**, Additional traces for mEPSC recorded at +40 mV in d60-75 neurons from EZH2i and DMSO groups. **e**, Quantification of amplitude and frequency of mEPSCs recorded at −70mV and +40 mV in d50 and d60-75 neurons from EZH2i versus DMSO conditions. −70mV frequency: DMSO d50 n = 9, EZH2i d50 n = 13, DMSO d60-75 n = 6, EZH2i d60-75 n = 8. −70mV amplitude: DMSO d50 n = 10, EZH2i d50 n = 15, DMSO d60-75 n = 6, EZH2i d60-75 n = 8. +40 mV frequency: DMSO d50 n = 10, EZH2i d50 n = 14, DMSO d60-75 n = 5, EZH2i d60-75 n = 7. +40 mV amplitude: DMSO d50 n = 10, EZH2i d50 n = 15, DMSO d60-75 n = 5, EZH2i d60-75 n = 8 neurons. **f**, Quantification of the NMDA/AMPA ratio in neurons at d50 and d60-75 from EZH2i and DMSO conditions. Averaged traces of the mEPSC are shown on the right. Error bands depict s.e.m. DMSO d50 n = 10; EZH2i d50 n = 15; DMSO d60-75 n = 4; EZH2i d60-75 n = 8. In (b) (e) (f) boxes extend from the 25th to 75th percentiles and the middle line represents the median; whiskers extend from min to max data points. Two-tailed t-test (c); two-tailed Welch’s test (b, e, f). Scale bars are 50 μm. FOV, field of view. Source Data

**Extended Data Fig. 6:**
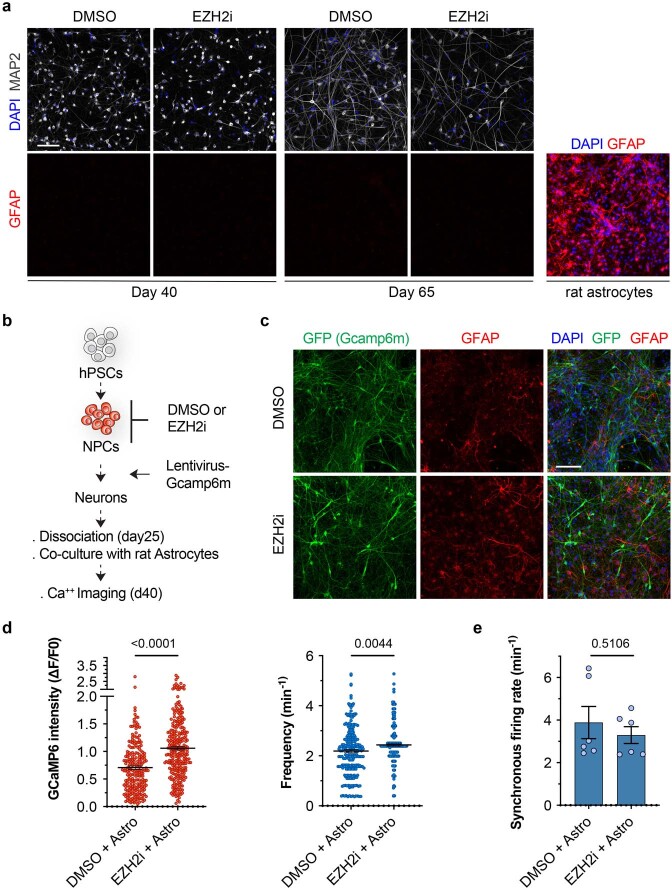
Transient inhibition of EZH2 triggers enhanced neuronal maturation even in the presence of astrocytes. **a**, Transient inhibition of EZH2 at NPC stage does not trigger the generation of astrocytes under synchronized neurogenesis conditions. Representative images of MAP2 and GFAP staining at d40 and d65 in cultures derived following the synchronized neurogenesis paradigm upon EZH2i versus DMSO. Primary rat astrocytes are positive control for the GFAP staining. **b**, Schematic of experimental paradigm for the Ca^2+^ imaging of hPSC-derived neurons in EZH2i and DMSO conditions in co-culture with primary rat astrocytes. hPSC-derived neurons were infected with GCamp6m lentiviruses four days before dissociation and prior to seeding onto rat primary astrocytes. **c**, Representative images of hPSC-derived neurons expressing GCamp6m in co-culture with rat primary astrocytes. **d**, Amplitude and frequency of spontaneous Ca^2+^ spikes of individual neurons. DMSO + Astro n = 225, EZH2i + Astro n = 264 (neurons from 2 independent experiments). **e**, Synchronicity rate of spontaneous network activity in EZH2i versus DMSO conditions in co-culture with rat astrocytes (n = 6 FOV from 2 independent experiments). Two-tailed Welch’s test (d, e) Scale bars are 100 μm. FOV, Field of View. Source Data

Neurons derived from NPCs treated with the EZH2 inhibitor appeared functionally similar to neurons derived from the DMSO control condition at early stage (d30) but rapidly became more excitable with increased numbers of evoked action potentials at d50 (Fig. 4g,h and Extended Data Fig. 5a,b). Furthermore, neurons derived from both human embryonic stem cells and iPSCs upon treatment with the EZH2 inhibitor at NPC stage showed increased densities of the presynaptic, postsynaptic and juxtaposed synaptic markers SYN1 and PSD95 at d65 (Fig. 4k and Extended Data Figs. 2f and 5c). There was a marked temporal increase in the frequency of AMPAR and NMDAR-mediated mEPSCs upon EZH2 inhibition, with a more subtle and delayed effect on amplitude (Fig. 4i,j and Extended Data Fig. 5d,e). The ratio of NMDAR-mediated to AMPAR-mediated currents was also increased (Extended Data Fig. 5f), as the emergence of strong NMDA currents remains a challenge in hPSC-derived neurons^45^. Of note, EZH2 inhibition in NPCs did not induce the emergence of astrocytes under synchronized neurogenesis conditions (Extended Data Fig. 6a), and the resulting neurons exhibited significant enhancement of amplitude and frequency of Ca^2+^ spikes relative to DMSO even when co-cultured on rat primary astrocytes (Extended Data Fig. 6b–d).

**Extended Data Fig. 7:**
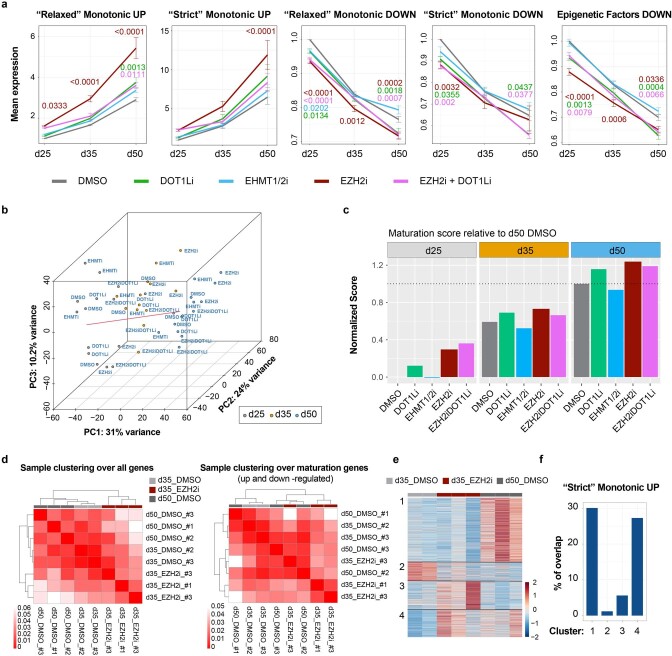
Temporal analysis of maturation signatures upon transient epigenetic inhibition and maturation scoring. **a**, RNA-seq analysis for the temporal expression of “relaxed” and “strict” monotonic up and down -regulated transcripts during maturation in neurons derived from NPC treated with inhibitors of epigenetic pathways. Data are normalized to d25 DMSO control condition and represented as mean ± s.e.m (n = 3 independent experiments). **b**, PCA plot for the sample distribution in the space defined by PCA1, 2 and 3. Red line indicate the vector for the maturation of control DMSO samples used as a reference to score epigenetic perturbations. **c**, Score for the maturation level achieved by neurons upon transient epigenetic inhibition at NPC stage compared to DMSO controls based on the dot product similarity metrics for RNA-seq samples in the coordinate system defined by 3 Principal Components (see Methods). **d**, Cross-correlation analysis based on the whole transcriptome (left) and based on the merged up and down -regulated maturation transcripts from lists in (a) (left) of RNA-seq samples of neurons from d35 EZH2i group with d35 and d50 neurons from DMSO condition. **e**, k-means clustering analysis of differentially expressed genes (with fold change > = 2 and FDR-adjusted p-value < 0.05, Wald’s test with Benjamini-Hochberg false discovery rate) between d25, d35 and d50 DMSO and between d35 EZH2i versus d35 DMSO contrasts. **f**, Percent overlap of the genes in cluster 1–4 from (e) with the “strict” monotonic upregulated transcript during maturation. Ordinary two-way ANOVA with Šídák’s multiple comparison test of treatment versus DMSO at each time point (a). Wald’s test with Benjamini-Hochberg false discovery rate (e). Source Data

In line with the faster acquisition of functionality (Fig. 4g–k and Extended Data Fig. 5), transient EZH2 inhibition in NPCs triggered a marked acceleration in the pace of transcriptional maturation in human neurons, which reached expression levels at d35 similar to d50 control neurons (Fig. 4l and Extended Data Fig. 7a). Despite the greater induction of maturation transcripts at d25, combined EZH2 and DOT1L inhibition only mildly accelerated transcriptional maturation at later stages (Extended Data Fig. 7a). Those data were further confirmed using a transcriptional maturation score (Extended Data Fig. 7b,c). Correlation analysis confirmed that d35 neurons following transient EZH2 inhibition clustered with d50 DMSO samples (Extended Data Fig. 7d), although the maturation effect of EZH2 inhibition was more obvious for maturation-related transcripts than for total transcripts. This analysis also revealed the induction of a subset of transcripts upon EZH2 inhibition that was not observed in DMSO d35 and d50 groups, which included a subset of ‘strict’ monotonic upregulated maturation genes (Extended Data Fig. 7e,f). Further studies will be needed to identify potential off-target signatures triggered by EZH2 inhibition. Together, these results indicate that transient EZH2 inhibition at the NPC stage primes human neurons for accelerated functional and transcriptional maturation.

## The epigenetic barrier in forebrain organoids

**Extended Data Fig. 8:**
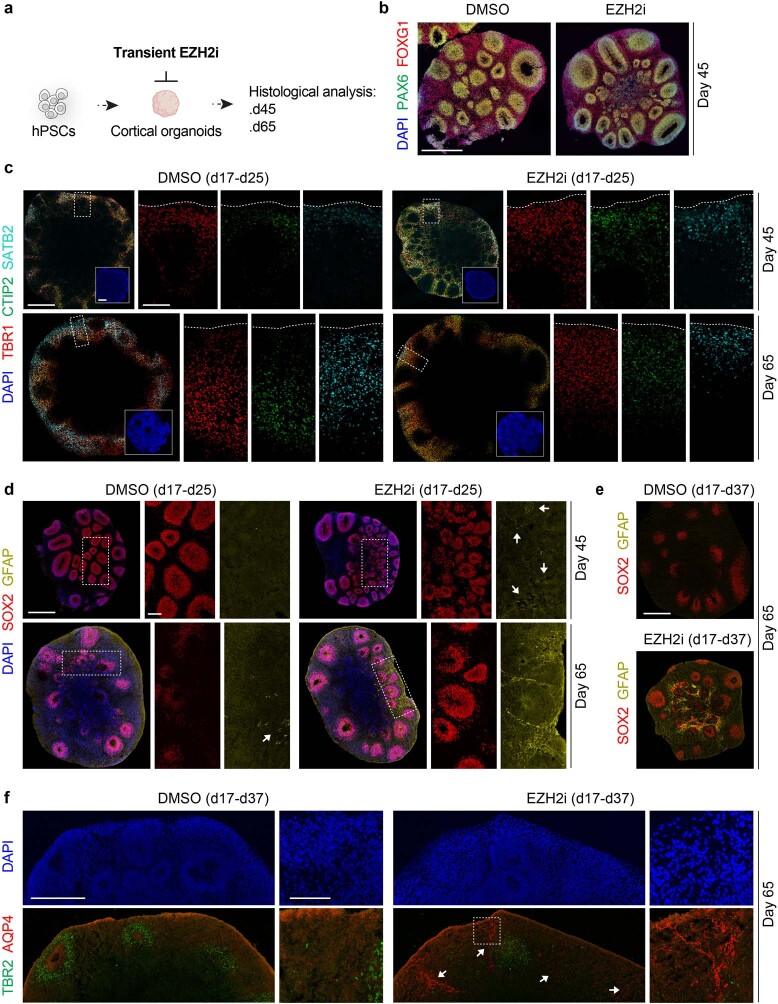
EZH2i accelerates cell-fate specification under spontaneous neurogenesis in human cortical 3D organoids. **a**, Schematic of experimental paradigm for transient inhibition of EZH2 in forebrain organoids. WA09 hPSC-derived organoids were transiently treated with GSK343 or DMSO after the specification of cortical identity (d17-d25 or d17-37 depending on the experiment as specified in the figure panels) and analysed for the generation of cortical cell identities using representative markers at d45 and d65. **b**, Representative images of FOXG1 and PAX6 staining in organoids at d45 showing the acquisition of cortical identity in EZH2i and DMSO control conditions. **c**, Representative images of organoids at d45 and d65 stained for the cortical layer markers TBR1, CTIP2 and SATB2 showing the reduction of SATB2-expressing neurons at d65 in EZH2i versus DMSO control conditions. Insets show the general morphology of organoids with the nuclei staining DAPI. **d**–**e**, Representative images of organoids at d45 and d65 stained for the NPC marker SOX2 and the astrocyte marker GFAP showing the precocious emergence of astrocytes surrounding SOX2^+^ neural rosettes in EZH2i versus DMSO control conditions (d-e). The appearance of GFAP+ cells is enhanced in longer treatment condition with EZH2 inhibitor GSK343 (e). **f**, Representative images of organoids at d65 stained with the intermediate progenitor cell marker TBR2 and the astrocyte marker AQP4 confirming the precocious emergence of astrocytes in EZH2i versus DMSO control conditions. Dotted boxes depict organoid regions showed at higher magnification in each panel. Arrows in (e) and (f) mark GFAP^+^ fibers and AQP4^+^ cells respectively. n = 5–6 organoids from 2 independent experiments per condition. Scale bars are 500 μm in whole organoid sections and 100 μm for the regions identified by dotted box and showed at higher magnification in all figure panels.

As for the 2D cultures (Fig. 4b), forebrain organoids were transiently treated with GSK343 following neural patterning (d17–d25) and neuronal activity was measured by Ca^2+^ imaging at d55 (Fig. 4m). These experiments also revealed a marked increase in the amplitude and frequency of Ca^2+^ spikes upon EZH2 inhibition versus DMSO treatments in 3D cultures (Fig. 4n and Supplementary Videos 7 and 8). Beyond our findings on post-mitotic neuron maturation, EZH2 has been reported to regulate the sequential specification of neurons and glia in the mouse cortex^14,15,17^. We therefore tested whether transient EZH2 inhibition similarly affects the timing of cell-fate specification during spontaneous neurogenesis in organoids (Extended Data Fig. 8a). EZH2 inhibition did not alter *PAX6* or *FOXG1* expression (Extended Data Fig. 8b). However, consistent with *Ezh2* deletion in mouse cortical NPCs^15^, EZH2 inhibition led to a slightly increased proportion of TBR1^+^ and CTIP2^+^ early born neurons at d45 and a reduction of superficial layer SATB2^+^ neurons at d65 (Extended Data Fig. 8c). Furthermore, we observed a precocious emergence of GFAP^+^ and AQP4^+^ astrocytes surrounding SOX2^+^ neural rosettes by d65, which was exacerbated with longer EZH2 inhibition (Extended Data Fig. 8d–f).

## The epigenetic barrier across species

**Extended Data Fig. 9:**
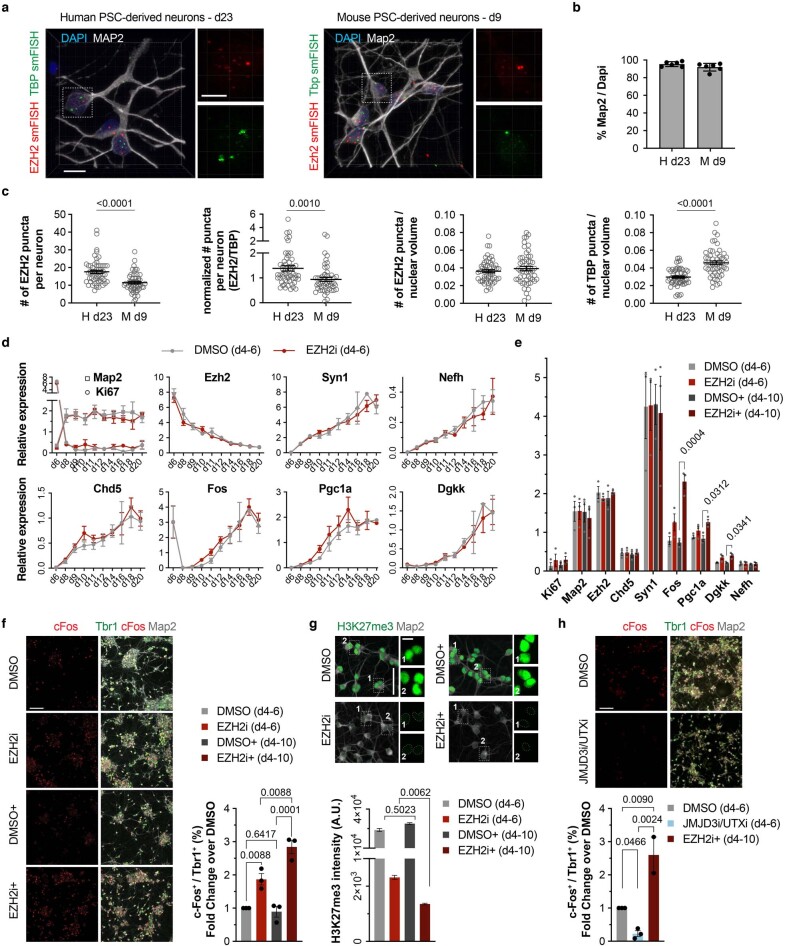
Impact of EZH2i on the maturation of mouse PSC-derived neurons. **a**–**c**, Representative images (a) and quantification (c) of smRNA FISH for *EZH2* and *TBP* coupled with MAP2 immunocytochemistry in the newly born neurons derived from hPSC and mEpiSC at d23 and d9 respectively. (b) depicts the fraction of neurons at comparable differentiation stages in human versus mouse neurons (n = 6 FOV from 2 independent experiments). (c) depicts neurons. Human d23 n = 57, mouse d9 n = 52 (neurons from 8 FOV from 2 independent differentiations). **d**, RT-qPCR expression of neuronal maturation markers upon treatment of mEpiSC-derived NPC with GSK343 and DMSO. d6 n = 5; d8 n = 5; d9 n = 4; d10 n = 5; d11 n = 4; d12 n = 5; d14 n = 5 (DMSO), n = 4 (Ezh2i); d16 n = 2 (DMSO), n = 3 (Ezh2i); d18 n = 2; d20 n = 3(independent experiments). **e**, RT-qPCR expression of maturation markers in mEpiSC-derived neurons at d12 upon different treatment durations with GSK343 and DMSO (n = 3 independent experiments). **f**, Representative images (left) and quantification (right) of the fraction of c-Fos^+^/Tbr1^+^ mEpiSC-derived neurons at d12 upon different durations of treatment with GSK343 and DMSO (n = 3 independent experiments). **g**, Representative images (top) and quantification (bottom) of the mean intensity of H3K27me3 staining in MAP2^+^ mEpiSC-derived neurons at d12. n = 30 neurons from 2 independent experiments. **h**, Representative images (top) and quantification (bottom) of the fraction of c-Fos^+^/Tbr1^+^ mEpiSC-derived neurons at d12 upon treatment with Ezh2i, DMSO and JMJD3i/Utxi. DMSO and JMJD3i/Utxi n = 3, EZH2i+ n = 2 (independent experiments). Ezh2i: 4uM GSK343 d4-6. Ezh2i +: 4uM GSK343 d4-6 plus 2uM GSK343 d6-10. JMJD3i/Utxi: 1uM GSK J4 d4−6. Histograms depict mean ± s.e.m. Two-tailed Welch’s test (c); ordinary one-way ANOVA with Holm-Šídák’s correction (e, f, h); Kruskal-Wallis test with Dunn’s correction (g). Scale bars are 100 μm (f, h); 50 μm (g); 10 μm (insets in g). FOV, field of view. Source Data

Neuronal maturation is particularly protracted in human neurons, raising the possibility that the epigenetic barrier is differentially regulated across species. To this end, we differentiated mouse epiblast stem cells via dual SMAD inhibition (Supplementary Fig. 8a). Similar to our hPSC differentiation (albeit at a faster pace), PAX6^+^ NPCs and forebrain neurons emerged by d3 and d9, respectively. Mouse neurons progressively upregulated maturation markers over approximately 20 days (Extended Data Fig. 9d and Supplementary Fig. 8b–e). We first measured the expression of *EZH2* and the housekeeping gene *TBP*, in the earliest born hPSC-derived neurons (d23) versus neurons derived from mouse epiblast stem cells (d9) by single-molecule RNA fluorescence in situ hybridization (smRNA-FISH). Mouse neurons expressed fewer *Ezh2* mRNA molecules—both absolute and normalized to *Tbp*—compared with human neurons. When normalized to nuclear volume, the number of EZH2 puncta was similar between species, but the ratio of EZH2 versus TBP puncta over nuclear volume was much lower in mouse neurons, consistent with a lower epigenetic barrier in mouse cells than in human cells (Extended Data Fig. 9a–c). Accordingly, transient EZH2 inhibition in NPCs led to only a mild upregulation of maturation markers in mouse neurons (Extended Data Fig. 9d–f). This effect became more pronounced for some markers upon increased duration of Ezh2 inhibition (Extended Data Fig. 9e,f) and correlated with a further reduction in global H3K27me3, the histone post-translational modification downstream of EZH2 (Extended Data Fig. 9g). Inhibition of JMJD3 (also known as KDM6B) and UTX (also known as KDM6A) H3K27 demethylases (which antagonize EZH2) with GSK-J4 in mouse NPCs resulted in decreased neuronal expression of the maturation marker *Fos* (Extended Data Fig. 9h). Although preliminary, these data are compatible with a delayed maturation of mouse neurons upon pharmacological enhancement of the mouse epigenetic barrier.

## The epigenetic barrier regulates poised genes

**Extended Data Fig. 10:**
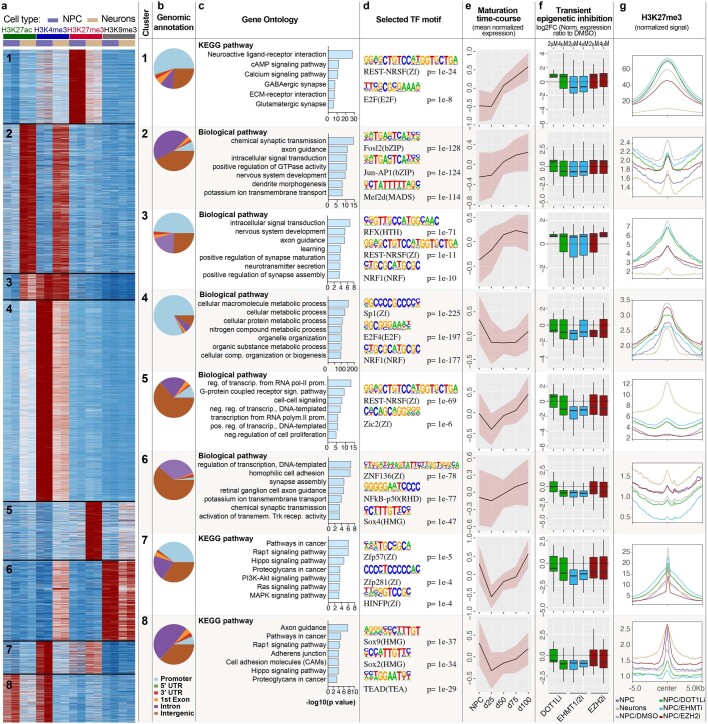
Patterns of histone post translational modifications drive the maturation of hPSC-derived neurons. **a**, Unsupervised clustering of CUT&RUN peaks with differential density in H3K27ac, H3K4me4, H3K27me3 and H3K9me3 signal among NPC and neurons (n = 2 replicates/condition). **b**, Pie charts of CUT&RUN peaks mapped to gene promoters, introns, exons, and intergenic genomic regions for each of the cluster. **c**, GO for genes linked at each cluster. **d**, Top selected statistically significant enriched transcription factor motifs at peaks in each cluster. **e**, Mean normalized expression (z-transform) of differentially expressed genes during the maturation time course intersected with genes linked to each CUT&RUN cluster. Error bands are s.e.m. **f**, Expression of differentially expressed transcripts from (e) that were also differentially expressed in neurons derived from NPC treated with the indicated inhibitors compared to DMSO controls (data from RNAseq studies, n = 3 independent experiments). **g**, Normalized signal of H3K27me3 density in NPC treated with epigenetic inhibitors and untreated NPC and neurons. Pink area in (e) is s.e.m. Box plots in (f) depict the median as center bar, the boxes span 25^th^ and 75^th^ percentiles and whiskers are 1.5*interquartile range. Binomial test (d). Source Data

We next characterized the dynamics of H3K27ac, H3K4me3, H3K27me3 and H3K9me3 in hPSC-derived cortical NPCs and neurons by CUT&RUN. Unsupervised clustering of peaks with differential binding of histone post-translational modification in NPCs versus neurons identified 8 distinct patterns (Extended Data Fig. 10a) and highlighted clusters 1, 2 and 3 as highly enriched for synapse-related genes (Extended Data Fig. 10c). We then intersected the genes linked to each CUT&RUN cluster with all differentially expressed genes identified by RNA-seq of NPCs and maturing (d25 to d100) neurons, irrespective of the directionality of the changes. This revealed a correlation between histone post-translational modification patterns in clusters 1, 2 and 3 and maturation-dependent transcription (Extended Data Fig. 10e). Furthermore, maturation-related genes from clusters 1, 2 and 3 comprised transcripts enriched in neurons derived from transient inhibition of DOT1L or EZH2 relative to DMSO (Extended Data Fig. 10f). Cluster 2 defined peaks with increased dual binding for H3K27ac and H3K4me3 in neurons, marking active chromatin domains at putative enhancers that were enriched for activity-dependent AP1 and MEF transcription factor motifs (Extended Data Fig. 10b,d). By contrast, cluster 1 was dominated by the dual presence of the EZH2-dependent repressive H3K27me3 mark and of active H3K4me3 mark at NPC stage. Such a poised or bivalent state^46^ is resolved toward active chromatin state in neurons via loss of H3K27me3 and acquisition of active H3K27ac (Fig. 5a and Extended Data Fig. 10a). Cluster 3 showed partial bivalency in NPCs but more pronounced H3K27ac induction in neurons (Extended Data Fig. 10a). These results indicate that the EZH2-dependent deposition of H3K27me3 at bivalent genes maintains maturation programmes in a poised state, which is supported by the decreased H3K27me3 signal at bivalent loci in NPC upon EZH2 inhibition (Fig. 5c,d and Extended Data Fig. 10g) and the subsequently increased expression of maturation genes (with bivalent status in NPC; Fig. 5a) in neurons derived from hPSCs and iPSCs upon transient EZH2 inhibition in NPCs (Fig. 5b and Extended Data Figs. 2e and 10f). Those transcripts follow the unperturbed, chronological maturation pattern (Figs. 1o and 2b–d) and participate in synaptic assembly and function, activity-dependent mechanisms (*FOS*, *FOSB* and *BDNF*), glycerolipid metabolism (*DGKK* and *DGKG)*, cytoskeleton maturation (*NEFH* and *TUBA4A*) and immunological programmes (*HLA-B* and *HLA-C*) (Fig. 5b). Of note, bivalent genes also included several chromatin regulator genes with maturation-dependent increased expression (Fig. 5e,f), reduced binding of H3K27me3 upon EZH2 inhibition (Fig. 5e) and dose-dependent induction in neurons upon NPC transient epigenetic inhibition (Fig. 5g). These include *JADE2*, which encodes a ubiquitin ligase for KDM1A^47^, whose loss of function triggered upregulation of maturation markers (Fig. 3d) and *CHD5*, which facilitates neuron-specific gene expression^48^. A functional interaction between EZH2 and JADE2 or CHD5, was further supported by the reduced effect of EZH2 inhibition on the amplitude and frequency of Ca^2+^ spikes in d40 neurons derived from CHD5 and JADE2-knockout hPSC lines (Fig. 5h and Supplementary Fig. 9), suggesting that these epigenetic regulators mediate accelerated maturation responses. Our results indicate that the epigenetic barrier controls the timing of neuron maturation via a dual mechanism—directly, by maintaining maturation genes in a poised state, and indirectly, by modulating the expression of competing epigenetic regulators promoting maturation (Fig. 5i,j).

## Discussion

hPSC technologies offer new approaches to investigate mechanisms of developmental timing. Here, we developed a method to synchronize the generation of cortical neurons from hPSCs, facilitating studies on neuronal maturation. This novel culture system is particularly suited to address neuron-intrinsic mechanisms, as it is largely devoid of glial cells, which are known to affect neuronal function^26,27^. A recent study on neural differentiation rather than neuronal maturation suggested that co-culture of mouse and human PSCs can modulate the timing of differentiation^49^. However, the extrinsic signals affecting timing in such chimeric cultures remain unclear. Finally, it will be valuable to compare maturation rates in neurons generated by different hPSC-based methods^45,50,51^ and to compare the emergence of mature NMDA neurotransmission^45^.

Our study mapped out several maturation phenotypes (synapses, metabolism, immunity-related and epigenetics). It is presently unclear whether independent mechanisms regulate each phenotype or whether a shared clock orchestrates their concerted expression. For example, mitochondrial maturation can regulate both morphological and functional aspects of neuronal maturation^39^. By contrast, human paralogues of the postsynaptic *SRGAP2A* gene^52^ are specifically associated with protracted synaptogenesis and spine morphogenesis by modulating ancestral gene function^53–55^.

Our data indicate that high expression levels and slow downregulation of the epigenetic barrier ensure the protracted human maturation timeline. Conversely, faster maturing mouse neurons seem to have a lower epigenetic barrier, with a smaller number of *Ezh2* transcripts. Accordingly, the induction of maturation markers in mouse neurons is only mildly enhanced by Ezh2 inhibition, and sustaining the epigenetic barrier via inhibition of JMJD3 and UTX H3K27 demethylases may delay their maturation. A recent study reported that translation of *Ezh2*, *Jmjd3* (akso known as *Kdm6b*) and *Utx* (also known as *Kdm6a*) affects the timing of fate specification in mouse NPCs^14^, implicating the contribution of both transcriptional and translational regulation. Distinct rates of transcription initiation and protein turnover correlate with species-specific timescales of early development^18,19,21^. However, further studies are needed to demonstrate causality and applicability to more protracted processes, including neuronal maturation. Energy metabolism^56^ and mitochondrial maturation^39^ also scale with species-specific maturation timing. Dissecting reciprocal interactions between metabolism and the epigenetic barrier may further identify the mechanisms underlying maturation timing. The epigenetic barrier comprises multiple classes of chromatin regulators, and further studies will need to dissect their interplay. For instance, EZH2 and DOT1L cooperate in regulating subsets of maturation genes. However, inhibition of EZH2 induced the expression of maturation genes, whereas inhibition of DOT1L, a pathway linked to stemness^57^, may promote maturation by silencing immaturity-related programmes. Other factors include *SOX4* and *SOX11*, which are downregulated with distinct temporal dynamics^58^ and can modulate cortical neuron maturation in vivo^59^.

Central to our study is the finding that transient inhibition of key epigenetic factors (EZH2, DOT1L and EHMT1/2) in NPCs enable accelerated post-mitotic neuronal maturation, indicating that maturation rates are ‘pre-programmed’ prior to neurogenesis. Furthermore, focusing on EZH2, we found that several maturation-related genes are transcriptionally poised in NPCs via H3K27me3 and H3K4me3 dual deposition. Reducing H3K27me3 levels via EZH2 inhibition accelerated the subsequent expression of many maturation-related transcripts, although further studies are needed to assess maturation-independent, potential off-target effects of EZH2 inhibition. Poised maturation genes comprised ‘effectors’ of neuronal maturity (such as ion channels and synaptic proteins), and other chromatin regulators (CHD5 and JADE2) which ‘competitively’ interact with epigenetic barriers^47,48,60,61^. Although our model highlights the release of repressive PRC2 and H3K27me3 as a driver of maturation timing, it does not negate the contribution of active chromatin-related factors. In fact, gain of H3K4me3 and H3K27ac in neurons also correlated with maturation-dependent transcription and loss of function of the H3K4 demethylases KDM5 or KDM1A also partially drives maturation, probably by globally increasing H3K4 methylation levels^62,63^.

EZH2 and H3K27me3 dynamics have been proposed to drive the temporal competence of NPCs to sequentially generate neurons versus glia in the mouse cortex^14,15,17^. Accordingly, transient EZH2 inhibition in organoids led to precocious specification of astrocytes, beside enhancing neuronal functionality. EZH2 downregulation in cortical NPC is linked to upregulation of maturation transcript during late neurogenesis^17^. This raises the intriguing possibility that a shared epigenetic machinery might coordinate the temporal specification of neuronal identities with the timing of post-mitotic maturation. Notably, the gradual downregulation of the epigenetic barrier is shared across excitatory and inhibitory neuron subtypes in the mouse and human cortex in vivo^41,44^. Our study focused on TBR1^+^ deep layer neurons and further studies should test whether the epigenetic barrier modulates maturation timelines in other neurons, including upper layer neurons. Finally, methods to manipulate maturation timing may enable improved hPSC-based technologies to interrogate emerging properties of human neuronal networks and to model adult-like neuronal function in neurological disease.

## Methods

### PSC lines and cell culture

Experiments with hPSCs and iPSCss was approved in compliance with the Tri-Institutional ESCRO at Memorial Sloan Kettering Cancer Center, Rockefeller University and Weill Cornell Medicine. hPSC lines WA09 (H9; 46XX) and WA01 (H1; 46XY) were from WiCell Stemcell Bank. The GPI::Cas9 line was derived from WA09 hPSCs. MSK-SRF001 iPSCs were from Memorial Sloan Kettering Cancer Center. hPSCs and iPSCs were authenticated by STR. hPSCs and iPSCs were maintained with Essential 8 medium (Life Technologies A1517001) in feeder-free conditions onto vitronectin-coated dishes (VTN-N, Thermo Fisher A14700). hPSCs and iPSCs were passaged as clumps every 4–5 days with EDTA (0.5 M EDTA/PBS) and routinely tested for mycoplasma contamination. The GPI::Cas9 knock-in hPSC line was generated using CRISPR–Cas9-mediated homologous recombination by transfecting H9 hPSCs with the Cas9-T2A-Puro targeting cassette downstream of the *GPI* gene (Supplementary Fig. 6b). Selected clones were validated by genomic PCR and Cas9 mRNA and protein expression by RT–qPCR and western blot, respectively and screened for Karyotype banding. CHD5-KO and JADE2-KO WA09 hPSC lines were generated by the SKI Stem Cell Research Core at Memorial Sloan Kettering Cancer Center (MSKCC) via CRISPR–Cas9 using the following gRNA targets: CHD5, CGTGGACTACCTGTTCTCGG; JADE2, CAGTTTGGAGCATCTTGATG. Mouse epiblast stem cells (EpiSCs) B6.129_4 were a gift from the Vierbuchen laboratory at Memorial Sloan Kettering Cancer Center and were maintained on mouse embryonic fibroblasts as previously described^64^. Rat primary astrocytes were purchased from Lonza (R-CXAS-520) and cultured according to manufacturer instructions.

### Synchronized generation of hPSC-derived cortical neurons

hPSCs (passage 40–50) were differentiated toward cortical excitatory neurons using an optimized protocol based on dual SMAD inhibition and WNT inhibition as follows. hPSCss were dissociated at single cells using Accutase and plated at 300,000 cells per cm^2^ onto Matrigel-coated wells (354234, Corning) in Essential 8 medium supplemented with 10 μM Y-27632. On day 0–2, cells were fed daily by complete medium exchange with Essential 6 medium (E6, A1516401, Thermo Fisher Scientific) in the presence of 100 nM LDN193189 (72142, Stem Cell Technologies), 10 μM SB431542 (1614, Tocris) and 2 μM XAV939 (3748, Tocris) to induce anterior neuroectodermal patterning. On day 3–9 cells were fed daily with Essential 6 medium (E6, A1516401, Thermo Fisher Scientific) in the presence of 100 nM LDN193189 (72142, Stem Cell Technologies), 10 μM SB431542. On day 10–20 cells were fed daily with N2/B27 medium (1:1 NB:DMEM/F12 basal medium supplemented with 1× N2 and B27 minus vitamin A) to generate a neurogenic population of cortical NPCs. N2 and B27 supplements were from Thermo. At day 20, NPCs were either cryopreserved in STEM-CELLBANKER solution (Amsbio) or induced for synchronized neurogenesis as following: NPCs were dissociated at single cells following 45 min incubation with Accutase and seeded at 150,000 cells per cm^2^ onto poly-l-ornithine and laminin/ fibronectin-coated plates in NB/B27 medium (1× B27 minus vitamin A, 1% l-glutamine and 1% penicillin-streptomycin in Neurobasal medium) in the presence of 10 μM Notch pathway inhibitor DAPT for 10 days (until day 30). For long-term culture, neurons were maintained in NB/B27 supplemented with BDNF (450-10, PreproTech), GDNF (248-BD-025, R&D biosystems), cAMP (D0627, Sigma) and ascorbic acid (4034-100, Sigma). From day 20 onwards, cells were fed every 4–5 days by replacing 50% of the medium volume. For neurons-astrocytes co-cultures, rat primary astrocytes were plated onto poly-l-ornithine and laminin/fibronectin-coated plates in NB/B27 medium supplemented with BDNF, GDNF, cAMP and ascorbic acid and allowed to adhere for few days. hPSC-derived neurons at day 25 were dissociated using Accutase and seeded on top of rat astrocytes. Neurons-astrocytes co-cultures were maintained on NB/B27 medium supplemented with BDNF, GDNF, cAMP and ascorbic acid.

### Mouse epiblast stem cell differentiation

Mouse epiblast stem cells (mEpiSCs) B6.129_4 were differentiated as following: on day 0, mEpiSC colonies were lifted from feeders using 0.5 U µl^−1^ collagenase IV in HBSS + +, dissociated to single-cell solution using Accutase, then plated at 220,000 cells per cm^2^ on Matrigel-coated wells in mN2/B27 media^64^ supplemented with 10 µM Y-27632, 100 nM LDN193189, 10 µM SB431542 and 2 µM XAV939. Cells were fed daily with mN2/B27 supplemented with 2 µM XAV939 (day 1), 100 nM LDN193189 (day 1–5), 10 µM SB431542 (day 1–5). On day 6 NPCs were dissociated to single-cell suspension using Accutase and replated at 200,000 cells per cm^2^ onto poly-l-ornithine and laminin/fibronectin-coated plates in NB/B27 medium (10% Neurobasal, 90% Neurobasal A, 1× B27 minus vitamin A, 1% Glutamax, 0.5% penicillin-streptomycin, 0.1% BDNF, 0.1% cAMP, 0.1% ascorbic acid, 0.1% GDNF) supplemented with 10 µM Y-27632 (day 6) and 10 µM DAPT (day 6 and 8). Cells were fed every other day by replacing 50% of the medium volume.

### Cerebral organoids differentiation

On day −1, WA09 (H9) hPSCs were dissociated with EDTA for 10 min at 37 °C and allowed to aggregate into spheroids of 10,000 cells each in V-bottom 96 well microplates (S-Bio) in E8 medium with ROCK inhibitor (Y-27632, 10 μM) and WNT inhibitor (XAV939, 5 μM, Tocris 3748). The next day (day 0), the medium was changed to E6 supplemented 100 nM LDN193189, 10 μM SB431542 and 5 μM XAV939. On day 5, medium was switched to E6 supplemented with 100 nM LDN193189, 10 μM SB431542. On day 8, medium was changed to N2/B27-based organoid medium as previously described^65^. From day 0 to day 14 medium was replaced every other day. On day 14, organoids were transferred to an orbital shaker on 10 cm dishes and half of the medium was changed on a Monday–Wednesday–Friday schedule. Treatment with 4 μM GSK343 or DMSO was performed transiently from day 17–25 or day 17–37 depending on the experiment as indicated in the figures.

### EdU labelling and small molecule treatments

For birth-dating experiments of WA09 (H9) hPSC-derived cortical neurons, 3μM EdU (5-ethynyl-2′-deoxyuridine, A10044 Invitrogen) was added to the culture for 48 h in the following time windows: d18–19, d20–21, d22–23, d24–25, d26–27, d28–29. After treatment, EdU was washed out and neurons were fixed at day40 of differentiation and processed for immunostaining. Treatment of hPSC-derived cortical NPCs with small molecules inhibitors of chromatin regulator was performed from day 12 to day 20 of differentiation (Fig. 4b). Small molecules were washed out and withdrawn starting at day 20 before the induction of synchronized neurogenesis and neurons derived from all the treatments were maintained in the same conditions. Small molecules were dissolved in DMSO and added to the N2/B27 medium at 2 or 4 μM depending on the experiment. DMSO in control conditions was added at the corresponding dilution factor as for epigenetic inhibitors.

Treatment of mEpiSC-derived NPCs was performed as follows: For Ezh2i experiments, 0.04% DMSO or 4μM GSK343 was added to NPC medium on day 4 and 5. For Ezh2i+ experiments this treatment was extended with 0.02% DMSO or 2μM GSK343 being added to medium on day 6, 8 and 10. GSK-J4 was used at 1 μM and added to the medium on day 4 and 5.

The following small molecules targeting epigenetic factors were used in the study and purchased from MedChemExpress: GSK343 (HY-13500), UNC0638 (HY-15273), EPZ004777 (HY-15227), GSK2879552 (HY-18632), CPI-455 (HY-100421), A-196 (HY-100201), GSK-J4 (HY-15648F). A List of small molecules and relative molecular target is reported in Extended Data Fig. 3b.

### Morphological reconstructions and quantification of synaptic puncta

For the morphological reconstruction of WA09 (H9) hPSC-derived neurons, NPCs were infected at day 20 with low-titre lentiviruses expressing dTomato reporter. Following induction of neurogenesis, the resulting neurons were fixed at day 25, 50, 75 and 100. The dTomato reporter signal was amplified by immunofluorescence staining and individual neurons were imaged at Zeiss AXIO Observer 7 epi-fluorescence microscope at 10× magnification. Neuronal morphology was reconstructed in Imaris v9.9.1 software using the filamentTracer function in autopath mode and using the nucleus (using DAPI channel) as starting point. Traces were eventually manually corrected for accuracy of cell processes detection. Neurite length and Sholl Analysis (every 10 μm radius) measurements were performed in the Imaris platform and extracted for quantifications and statistics. For staining with synaptic markers, cells were cultured on μ-plate 96 Well Black (Ibidi) and stained for SYN1 and PSD95 antibodies to visualize pre and post -synaptic puncta respectively and MAP2 to visualize neuronal dendrites. Confocal images were acquired using a 63× immersion objective at a Leica SP8WLL confocal laser-scanning microscope. Three fields of view for each sample from two independent differentiations (total of 6 fields of view per condition) were analysed as following. Single-plane confocal images were open in Fiji v2.9.0 and puncta were detected using the SynQuant plugin (https://github.com/yu-lab-vt/SynQuant). The *z*-score for particle detection was adjusted for accuracy of puncta detection. The other parameters were set as default value. Dendrite length was extracted from the reference MAP2 channel.

### Immunocytochemistry and histology

Cultured cells were fixed with 4% PFA in PBS for 20 min at RT, washed three times with PBS, permeabilized for 30 min in 0.5% Triton X-100 in PBS and then blocked in a solution containing 5% Normal goat serum or Normal donkey serum, 2% BSA and 0.25% Triton X-100 for 1 h at room temperature. Primary antibodies were incubated overnight at 4 °C in the same blocking solution. EdU^+^ cells were detected using the Click-iT EdU Imaging kit (Molecular Probes) with Alexa Fluor 488 according to manufacturer’s instructions. Secondary antibodies conjugated to either Alexa 488, Alexa 555 or Alexa 647 (Thermo) were incubated for 45 min at 1:400 dilution in blocking solution. Cell nuclei were stained with 5 μM 4′-6-diamidino-2-phenylindole (DAPI) in PBS.

Organoids were fixed in 4% PFA overnight at 4 °C, washed 3 times with PBS and cryoprotected in 30% sucrose/PBS. Organoid tissue was sectioned at 30 μm on a cryostat (Leica 3050 S), mounted on microscope slides, allowed to dry at room temperature and stored at −80 °C. On the day of the staining, slides we defrosted for 20 min at room temperature. Sections were first permeabilized in 0.5% Triton X-100 in PBS, blocked for 1 h in 5% normal goat serum, 1% BSA, 0.25% triton in PBS and incubated in the same solution with primary antibodies overnight. The next day, sections were washed with PBS and incubated in secondary antibodies for 2.5 h at room temperature at 1:400 dilution. DAPI 5 μM stain was used to identify cell nuclei. Images were captured using a Leica SP8WLL confocal laser-scanning microscope.

The following primary antibodies and dilutions were used: rabbit anti-PAX6 1:300 (901301, Biolegend); rabbit anti-FOXG1 1:500 (M227, Clonetech); mouse anti-Nestin 1:400 (M015012, Neuromics); mouse anti-MAP2 1:200 (M1406, Sigma); chicken anti-MAP2 1:2000 (ab5392, Abcam); rabbit anti-class III β-tubulin (TUJI) 1:1,000 (MRB-435P, Covance); mouse anti-Ki67 1:200 (M7240, Dako); rabbit anti-Ki67 1:500 (RM-9106, Thermo Scientific); rabbit anti-TBR1 1:300 (ab183032, Abcam); rabbit anti-TBR1 1:500 (ab31940, Abcam); rat anti-CTIP2 1:500 (ab18465, Abcam); mouse anti-SATB2 1:1,000 (ab51502, Abcam); rabbit anti-synapsin I 1:1,000 (S193, Sigma); mouse anti-PSD95 1:500 (MA1-046, Thermo); mouse anti-neurofilament H 1:500 (non-phosphorylated) (SMI32, Enzo Life science); mouse anti c-FOS 1:500 (ab208942, Abcam); mouse anti-HLA Class I ABC 1:150 (ab70328, abcam); goat anti-RFP 1:1,000 (200-101-379, Rockland); rabbit anti-DsRed 1:750 (632496, Clontech); rabbit anti-H3K27me3 1:200 (9733, Cell Signaling Technologies); rabbit anti-GFAP 1:500 (Z033429-2, Dako); chicken anti-GFP 1:1,000 (ab13970, Abcam); rat anti-SOX2 1:200 (14-9811-82, Thermo); rabbit anti-AQP4 1:500 (HPA014784, SIGMA); sheep anti-EOMES 1:200 (AF6166, R&D). The primary antibodies including anti-GFAP antibody were validated for recognition of human antigens to confirm lack of human astrocytes in our synchronized cortical cultures.

### smRNA-FISH

smRNA-FISH was performed on WA09 (H9) hPSC-derived and mEpiSC-derived neurons using ViewRNA Cell Plus Assay Kit (Invitrogen) in RNAse-free conditions according to manufacturer’s instructions to simultaneously detect RNA targets by in situ hybridization and the neuronal marker MAP2 (Alexa Fluor 647) by immunolabelling. Neurons were plated on μ-plate 24 Well Black (Ibidi) plates, fixed and permeabilized for 15 min at room temperature with fixation/permeabilization solution and blocked for 20 min followed by incubation with primary and secondary antibody for 1 h at room temperature. Target probe hybridization with mouse or human -specific viewRNA Cell Plus probe sets was carried at 40 °C under gentle agitation for 2 h. Type 1 (Alexa Fluor 546) and type 4 (Alexa Fluor 488) probe sets were used to detect EZH2 and TBP RNA respectively, using the same fluorophore scheme for neurons derived from mEpiSCs and hPSCs. Pre amplification, amplification and fluorescence labelling steps were carried at 40 °C under gentle agitation for 1 h each. Washes were performed as indicated in the kit’s procedure. Samples were incubated with 5 μM DAPI to visualize cell nuclei and a coverslip was gently placed inside each well using ProLong Glass Antifade Mountant. *z*-stack images at 0.4 μm step and covering the entire cell volume were acquired using a Leica SP8WLL confocal laser-scanning microscope with a 63× immersion objective at 3× digital zoom. *z*-stacks were loaded and projected in Imaris v9.9.1 software for RNA puncta visualization and quantification within each single MAP2 positive neuron. Eight different fields of view (2–5 neurons per field) for each condition (mouse versus human) from two independent batches of differentiations (16 fields of view per condition) were obtained for downstream analysis. The nuclear volume for each neuron was reconstructed and calculated using the Surface function in Imaris Software.

### Electrophysiological recording

For electrophysiological recordings, neurons were plated in 35 mm dishes. Whole-cell patch clamp recordings during the maturation time course were performed at day 25, 50, 75 and 100 of differentiation as previously described^22^. In brief, neurons were visualized using a Zeiss microscope (Axioscope) fitted with 4× objective and 40× water-immersion objectives. Recordings were performed at 23–24 °C and neurons were perfused with freshly prepared artificial cerebral-spinal fluid (aCSF) extracellular solution saturated with 95% O_2_, 5% CO_2_ that contained (in mM): 126 NaCl, 26 NaHCO_3_, 3.6 KCl, 1.2 NaH_2_PO_4_, 1.5 MgCl_2_, 2.5 CaCl_2_, and 10 glucose. Pipette solution for recordings in current clamp configuration contained (in mM): 136 KCl, 5 NaCl, 5 HEPES, 0.5 EGTA, 3 Mg-ATP, 0.2 Na-GTP, and 10 Na_2_-phosphocreatine, pH adjusted to 7.3 with KOH, with an osmolarity of ~290 mOsm. For mEPSCs, the pipette solution contained (in mM): 140 CsCl, 10 NaCl, 10 HEPES, 0.5 EGTA, 3 Mg-ATP, 0.2 Na-GTP, and 10 Na_2_-phosphocreatine, pH adjusted to 7.3 with CsOH. 20 μM (−)-bicuculline methochloride (Tocris), 1 μM strychnine HCl (Sigma), and 0.5 μM tetrodotoxin (TTX) (Alomone Labs) were added to aCSF for mEPSC recordings to block GABA_A_ receptors, glycine receptors, and voltage-gated Na^+^ channels, respectively. Input resistance was measured from a voltage response elicited by intracellular injection of a current pulse ( − 100 pA, 200 ms). Membrane voltage was low-pass filtered at 5 kHz and digitized at 10 kHz using a Multiclamp 700B amplifier connected to a DigiData 1322 A interface (Axon Instruments) using Clampex 10.2 software (Molecular Devices). Liquid junction potentials were calculated and corrected off-line. Action potentials were generated in current clamp from currents injected in 10 pA intervals from 0 to 250 pA. Recordings were analysed for: resting membrane potential, input resistance, rheobase, threshold, as well as action potential amplitude, overshoot, duration, half-width, rise and decay. Neurons were held at −80 mV and continuous recordings of mEPSCs were made using Axoscope software (Molecular Devices). Data processing and analysis were performed using MiniAnalysis (Synaptosoft) version 6 and Clampfit 10.2 (Molecular Devices). Events were detected by setting the threshold value, followed by visual confirmation of mEPSC detection. Whole-cell patch clamp recordings in neurons derived from DMSO and EZH2i conditions (pipettes 3–6 MΩ) were performed in aCSF containing (in mM): 125 NaCl, 2.5 KCl, 1.2 NaH_2_PO_4_, 1 MgSO_4_, 2 CaCl_2_, 25 NaHCO_3_ and 10 d-glucose. pH and osmolarity were adjusted to 7.4 and 300–310 mOsm, respectively. For firing recordings, pipettes were filled with a solution containing (in mM): 130 potassium gluconate, 4 KCl, 0.3 EGTA, 10 Na_2_-phosphocreatine, 10 HEPES, 4 Mg_2_-ATP, 0.3 Na_2_-GTP and 13 biocytin. pH and osmolarity were adjusted to 7.3 (with KOH) and 285–290 mOsm respectively. For mEPSCs recordings the ACSF was supplemented with 1 µM TTX and 100 µM 4-AP and pipettes were filled with a caesium-based solution that contained (in mM): 120 CsMeSO_4_, 8 NaCl, 10 HEPES, 0.3 EGTA, 10 TEA-Cl, 2 Mg_2_-ATP, 0.3 Na_2_-GTP, 13.4 biocytin and 3 QX-314-Cl. pH: 7.3 (adjusted with CsOH) and 290–295 mOsm. Recordings were acquired with a computer-controlled Multiclamp 700B amplifier and a Digidata 1550B (Molecular Devices, California) at a sampling rate of 10 kHz and low-pass filtered at 1 kHz. pClamp 10 software suite (Molecular Devices) was used for data acquisition (Clampex 10.6) and data analysis (Clampfit 10.6). The quantification of the amplitude and inter-event interval of mEPSCs shown in the cumulative probability plots in Fig. 4j was performed taking all the events together. To isolate the NMDA component from mEPSCs recorded at +40 mV, we measured current amplitude 20 ms after the mEPSC onset, where AMPA receptors are desensitized (depicted by the dotted line in Extended Data Fig. 5f)^66–68^. For the calculation of the NMDA/AMPA ratio, the amplitude of the NMDA component was then divided by the amplitude of the peak of the AMPA currents recorded at – 70 mV. Statistical analysis and plots were done in Prism 9 (GraphPad, California). Evoked action potential and traces shown in DMSO versus EZH2i groups in Fig. 4g were elicited with 20 pA injected current.

### Calcium imaging and analysis

hPSC-derived cortical neurons were infected with lentiviruses encoding GCaMP6m and cultured on μ-plate 96 Well Black (Ibidi). In rat astrocytes co-culture experiments, hPSC-derived neurons were infected with GCaMP6m lentiviruses four days before dissociation and prior to seeding onto rat primary astrocytes. For each batch of experiments, the infection and measurement of Ca^2+^ spikes in neurons under control or genetic/pharmacological perturbation has been done in parallel on the same day to account for the variability in the absolute expression of GCaMP6m due to lentiviral delivery. Ca^2+^ imaging was performed as previously described^69^. In brief, on the day of the imaging, cells were gently washed twice in modified Tyrode solution (25 mM HEPES (Invitrogen), 140 mM NaCl, 5 mM KCl, 1 mM MgCl_2_, 10 mM glucose, 2 mM CaCl_2_, 10 μM glycine, 0.1% BSA pH 7.4, pre-warmed to 37 °C) and equilibrated in imaging buffer for 1-2 min (25 mM HEPES, 140 mM NaCl, 8 mM KCl, 1 mM MgCl_2_, 10 mM glucose, 4 mM CaCl_2_, 10 μM glycine, 0.1% BSA pH 7.4, pre-warmed to 37 °C). GCaMP6m fluorescence was recorded on Celldiscover7 (ZEISS) inverted epi-fluorescence microscope with the 488 nm filter under environmental control (37 °C; 95% O_2_, 5% CO_2_) using ZEN Blue 3.1 software at the Bio-Imaging Resource Center (BIRC) at Rockefeller University. Neuronal cultures were imaged for ~3 min at a frame rate of 4–6 frames per second (600–800 frames per time lapse) using a 10× or 20× objective.

hPSC-derived cortical brain organoids were infected with lentiviruses encoding GCaMP6m at day 45 of differentiation and cultured in BrainPhys Imaging Optimized Medium (Stem Cell Technologies) for a week before the imaging. On the day of the imaging, DMSO control and organoids transiently treated with GSK343 were equilibrated in imaging buffer for 30 min and transferred into imaging cuvettes. GCaMP6m fluorescence on intact organoids was recorded by light-sheet microscopy on TruLive3D Imager (Bruker) under environmental control (37 °C; 95% O2 – 5% CO2). Multiple fields of view from 3–4 organoids per condition from 2 independent batches each were imaged for ~2–4 min at a frame rate of 5–10 frames per second at 31.3× effective magnification.

Analysis was performed as previously described^69^. In brief, the live-imaging image stack was converted to TIFF format and loaded into optimized scripts in MATLAB (Mathworks) R2020b and R2021b. Region of Interest (ROI) were placed on the neuron somas to calculate the raw GCaMP6m intensity of each neuron over time. The signal intensity of each raw trace was normalized to baseline fluorescence levels (Δ*F*/*F*_0_) for spike detection. Single-neuron amplitude was calculated from the normalized GCaMp6m intensity for all the detected spikes in each trace (mean Δ*F*/*F*_0_ of detected spikes for each neuron). Single-neuron frequency was calculated as the number of detected spikes in each trace per minute of recording. Network activity was assessed by calculating the synchronous firing rate, defined as the number of detected synchronous Ca^2+^ spikes from all ROI in one FOV per minute of recording. In Figs. 1k and 4k, coloured lines depict the normalized (Δ*F*/*F*_0_) GCaMP6m signal traces of individual neurons in 1 field of view during 1 min of imaging; the black line is the averaged normalized GCaMP6m signal among neurons in 1 field of view. Images in Figs. 1j Fig. 4m were displayed as royal lookup table in FIJI. Supplementary Videos 1–6 show 20 frames per second, Supplementary Videos 7 and 8 show 100 frames per second.

### Image analysis and quantification

Microscopy images were visualized with Adobe Photoshop 2022, Fiji 2.9.0 or Imaris software version 9.9.1. Morphological reconstruction of neurons was performed using Imaris software version 9.9.1. Ca^2+^ imaging analysis was performed using MATLAB software. Quantification of immunofluorescence images was performed in FIJI (ImageJ) version 2.9.0 or using the Operetta high content imaging system coupled with Harmony software version 4.1 (PerkinElmer).

### Protein extraction and western blot

Cells were collected and lysed in RIPA buffer (Sigma) with 1:100 Halt Protease and Phosphatase Inhibitor Cocktail (Thermo Fisher Scientific) and then sonicated for 3× 30sec at 4 °C. Protein lysates were centrifugated for 15 min at more than 15,000 rpm at 4 °C and supernatant was collected and quantified by Precision Red Advanced Protein Assay (Cytoskeleton). 5–10 μg of protein were boiled in NuPAGE LDS sample buffer (Invitrogen) at 95 °C for 5 min and separated using NuPAGE 4–12% Bis-Tris Protein Gel (Invitrogen) in NuPAGE MES SDS Running Buffer (Invitrogen). Proteins were electrophoretically transferred to nitrocellulose membranes (Thermo Fisher Scientific) with NuPAGE Transfer Buffer (Invitrogen). Blots were blocked for 60 min at room temperature in TBS-T + 5% nonfat milk (Cell Signaling) and incubated overnight in the same solution with the respective primary antibodies at 4 °C. The following primary antibodies were used: mouse anti-neurofilament H 1:500 (non-phosphorylated) (SMI32; Enzo Life science); mouse anti-syntaxin 1 A 1:500 (110 111; SYSY); mouse anti-actin 1:500 (MAB1501; Millipore); mouse anti-Cas9 1:500 (14697; Cell Signaling Technology); rabbit anti-CHD3 1:1,000 (ab109195, Abcam); rabbit anti-KDM5B 1:1,000 (ab181089, abcam). The following secondary antibodies were incubated for 1 h at room temperature at 1:1,000 dilution: anti-mouse IgG HRP-linked (7076; Cell Signaling Technology) and anti-rabbit IgG HRP-linked (7074; Cell Signaling Technology). Blots were revealed using SuperSignalTM West Femto Chemiluminescent Substrate (Thermo Fischer Scientific) at ChemiDoc XRS+ system (Bio-Rad). Chemiluminescence was imaged and analysed using Image lab software version 6.1.0 (Bio-Rad). Controls samples were run within each gel and the signal intensity of protein bands of interest was normalized to the intensity of the actin band (loading control) for each sample on the same blot. Uncropped and unprocessed images are shown in Supplementary Figure 1. One sample *t*-test on Fig. 3d was performed by comparing the mean of logFC for each genetic perturbation with the hypothetical mean logFC = 0 (null hypothesis of no changes). Two-tailed ratio-paired *t*-test in Fig. 4c was calculated on normalized marker/actin expression in manipulations versus DMSO.

### RNA isolation and RT–qPCR

Samples were collected in Trizol. Total RNA from hPSC-derived samples was isolated by chloroform phase separation using Phase Lock Gel-Heavy tubes, precipitated with ethanol, and purified using RNeasy Mini Kit (Qiagen) with on-column DNA digestion step. RNA from mouse cells was isolated using Direct-zol microprep kit (Zymo research, R2060). cDNA was generated using the iScript Reverse Transcription Supermix (Bio-Rad) for RT–qPCR and quantitative PCR (qPCR) reactions were performed using SsoFast EvaGreen Supermix (Bio-Rad) according to the manufacturer’s instructions in 96 or 384-well qPCR plates using CFX96 and CFX384 Real-Time PCR Detection systems (Bio-Rad) using 5–10 ng cDNA / reaction. Primers were from Quantitect Primer assays (QUIAGEN) except for the ones in Supplementary Table 4. Results were normalized to the housekeeping genes *GAPDH* or *TBP*.

### DNA constructs and lentivirus production

A Cas9-T2A-PuroR cassette flanked by 5′ and 3′ homology arms for the *GPI* locus was generated by NEBuilder HiFi DNA Assembly Cloning Kit of PCR-amplified fragments according to manufacturer’s instruction. EF1alpha-GCaMP6m lentiviral vector was generated by PCR amplification of GCaMP6m from pGP-CMV-GCaMP6m (Addgene 40754) using with Q5 High Fidelity master mix (NEB) and subcloned into pWPXLd (Addgene 12258) into BamH1 and EcoRI restriction site using standard cloning methods. For the simultaneous expression of gene-specific gRNA under transcriptional control of U6 promoter and dTomato fluorescent reporter driven by EF1alpha promoter, the SGL40.EFs.dTomato vector (Addgene 89398) was modified by inserting a P2A-Basticidin cassette downstream of dTomato sequence to generate the SGL40.EFs.dTomato-Blast backbone. gRNA sequences specific to each gene were designed using SYNTEGO CRISPR design tool (https://www.synthego.com/products/bioinformatics/crispr-design-tool) and validated using CRISPOR tool^70^ (http://crispor.tefor.net). DNA oligos (IDT) were annealed and subcloned into BsmBI restriction sites of SGL40.EFs.dTomato-Blast lentiviral backbone by standard cloning methods. Lentiviruses were produced by transfection of HEK293T cells (ATCC) using the Xtreme Gene 9 DNA transfection reagent (Sigma) with the respective lentiviral vectors along with the packaging vectors psPAX2 (Addgene, 12260) and pMD2.G (Addgene, 12259). Arrayed CRISPR gRNA lentiviral libraries were produced simultaneously. Viruses were collected 48 h post transfection, filtered with 0.22-μm filters and stored in aliquots at −80 °C. The sequence of each gRNA used is reported in Supplementary Table 5.

### RNA-seq sample processing and analysis

Total RNA was extracted as described above. Sample for RNA-seq during chronological maturation at hPSC, NPC, d25, d50, d75 and d100 timepoints were submitted for TruSeq stranded ribo-depleted paired-end total RNA-seq at 40–50 million reads at the Epigenomic Core at Well Cornell Medical College (WCMC). Samples for RNA-seq studies on neurons upon perturbation with epigenetic inhibitors were submitted for paired-end poly-A enriched RNA-seq at 20–30 million reads to the MSKCC Integrated Genomic Core. Quality control of sequenced reads was performed by FastQC. Adapter-trimmed reads were mapped to the hg19 human genome using versions 2.5.0 or 2.7.10b of STAR^71^. The htseq-count function of the HTSeq Python package version 0.7.1^72^ was used to count uniquely aligned reads at all exons of a gene. For the chronological maturation studies, the count values were transformed to RPKM to make them comparable across replicates. A threshold of 1 RPKM was used to consider a gene to be present in a sample and genes that were present in at least one sample were used for subsequent analyses. Differential gene expression across timepoints or treatments with epigenetic inhibitors was computed using versions 1.16 or 1.22.2 of DESeq2 respectively^73^. Variance stabilizing transformation of RNA-seq counts was used for the PCA plots and for heat maps of gene expression. For downstream analysis of trends of gene expression, transcripts were first grouped into ‘monotonically upregulated’ and ‘monotonically downregulated’ based on the characteristics of their expression from d25 to d100 and further categorized in strict: all the transitions satisfy the statistical significance criteria and relaxed: d25 versus d100 transition satisfy the significance criteria and intermediate transitions may not. For all comparisons a significance threshold of false discovery rate (FDR) ≤ 5% was used. Monotonically upregulated (strict): (d50 versus d25*:* FDR ≤ 5%) and (d100 versus d25: FDR ≤ 5%) and (d100 versus d50: FDR ≤ 5%) and (d50 versus d25*:*logFC > 0) and (d75 versus d50: logFC > 0) and (d100 versus d25 logFC > d50 versus d25 logFC). Monotonically downregulated (strict): (d50 versus d25*:*FDR ≤ 5%) and (d100 versus d25*:* FDR ≤ 5%) and (d100 versus d50: FDR ≤ 5%) and (d50 versus d25:logFC <0) and (d75 versus d50: logFC <0) and (d100 versus d25 logFC <d50 versus d25 logFC). Monotonically upregulated (relaxed): (d100 versus d25: FDR ≤ 5%) and (d50 versus d25:logFC > 0) and ((d100 versus d25:logFC >= d50 versus d25: logFC) OR (d75 versus d50: logFC > 0)). Monotonically downregulated (relaxed): (d100 versus d25: FDR ≤ 5%) and (d50 versus d25:logFC <0) and ((d100 versus d25:logFC <= d50 versus d25: logFC) OR (d75 versus d50: logFC <0)). GSEA^74^ was performed on d50 versus d25 and d100 versus d50 pairwise comparisons to test enrichment in KEGG pathways or gene sets from MSigDB using the following parameters: FDR ≤ 5%, minimum gene-set size=15, maximum gene-set size=500, number of permutations = 1000. GO term analysis was performed using v6.8 DAVID^75^ (http://david.abcc.ncifcrf.gov/knowledgebase/). Venn diagrams were generated using Biovenn^76^.

The score for maturation in neurons upon epigenetic inhibition and control conditions (Extended Data Fig. 7b,c). was computed based on the geometric distribution of samples in the three-dimensional coordinate system defined by PCA1, 2 and 3. For each condition (treatment and day of differentiation), the coordinates defining the position of the samples in the 3D PCA space were determined based on the average across replicates. The DMSO d25 coordinates were set as the origin. The vectors defining maturation trajectories for each treatment and timepoint were then measured as the connecting segments between sample coordinates. The vector linking DMSO d25 and DMSO d50 conditions was used to define the chronological maturation trajectory and set as a reference (control vector) to calculate a similarity score for each treatment at any given timepoint. To account for vector magnitude and directionality, the dot product metric treatment vector · control vector was used to calculate the scores. Gene expression correlation heat maps in Extended Data Fig 7d were created from either all genes or maturation genes only by computing Pearson correlation and then running agglomerative hierarchical clustering using complete linkage. *k*-Means clustering in Extended Data Fig 7e was performed on *z*-score converted normalized counts and run using the kmeans function in R with nstart = 25 and *k* = 2:10, stopping when clusters became redundant (*k* = 4).

### ATAC-seq sample processing and analysis

ATAC-seq libraries were prepared at the Epigenetic Innovation Lab at MSKCC starting from ~50,000 live adherent cells plated on 96-wells. Size-selected libraries were submitted to the MSKCC Genomic core for paired-end sequencing at 40–60 million reads. Quality control of sequenced reads was performed by FastQC (version 0.11.3) and adapter filtration was performed by Trimmomatic version 0.36. The filtered reads were aligned to the hg19 reference genome. Macs2 (version 2.1.0)^77^ was used for removing duplicate reads and calling peaks. Differentially accessible peaks in the atlas were called by DESeq2 version 1.16^73^. To define dynamic trends of chromatin accessibility during neuronal maturation as shown in Fig. 3g, agglomerative hierarchical clustering using Ward’s linkage method was done on the union of differentially accessible peaks in pairwise comparisons between d25, d50, d75 and d100 samples. HOMER findMotifsGenome.pl (version 4.6)^78^ was used to investigate the motif enrichment in pairwise comparisons and unbiasedly clustered groups of peaks. Motif enrichment was also assessed by Kolmogorov–Smirnov and hypergeometric tests as previously described^79^. ATAC-seq peaks in the atlas were associated with transcription factor motifs in the updated CIS-BP database^80,81^ using FIMO^82^ of MEME suite version 4.11^83^. Hypergeometric test was used to compare the proportion of peaks containing a transcription factor motif in each group (foreground ratio) with that in the entire atlas (background ratio). Odds ratio represents the normalized enrichment of peaks associated with transcription factor motifs in the group compared to the background (foreground ratio/background ratio). Odds ratio ≥ 1.2 and transcription factor expression from parallel RNA-seq studies (reaching ≥ 1 RPKM) in neurons at any timepoint (d25, d50, d75, d100) was used to filter enriched transcription factor motif.

### CUT&RUN sample processing and analysis

CUT&RUN was performed from 50,000 cells per condition as previously described^84^ using the following antibodies at 1:100 dilution: rabbit anti-H3K4me3 (aab8580, abcam); rabbit anti-H3K9me3 (ab8898, abcam); rabbit anti-H3K27me3 (9733, Cell Signaling Technologies); rabbit anti-H3K27ac (309034, Active Motif), normal rabbit IgG (2729, Cell Signaling Technologies). In brief, cells were collected and bound to concanavalin A-coated magnetic beads after an 8 min incubation at room temperature on a rotator. Cell membranes were permeabilized with digitonin and the different antibodies were incubated overnight at 4 °C on a rotator. Beads were washed and incubated with pA-MN. Ca^2+-^induced digestion occurred on ice for 30 min and stopped by chelation. DNA was finally isolated using an extraction method with phenol and chloroform as previously described^84^. Library preparation and sequencing was performed at the MSKCC Integrated Genomic Core.

Sequencing reads were trimmed and filtered for quality and adapter content using version 0.4.5 of TrimGalore (https://www.bioinformatics.babraham.ac.uk/projects/trim_galore) and running version 1.15 of cutadapt and version 0.11.5 of FastQC. Reads were aligned to human assembly hg19 with version 2.3.4.1 of bowtie2 (http://bowtie-bio.sourceforge.net/bowtie2/index.shtml) and MarkDuplicates of Picard Tools version 2.16.0 was used for deduplication. Enriched regions were discovered using MACS2 with a p-value setting of 0.001 and a matched IgG or ‘no antibody” as the control. The BEDTools suite version 2.29.2 (http://bedtools.readthedocs.io) was used to create normalized read density profiles. A global peak atlas was created by first removing blacklisted regions (https://www.encodeproject.org/annotations/ENCSR636HFF) then merging all peaks within 500 bp and counting reads with version 1.6.1 of featureCounts (http://subread.sourceforge.net). Reads were normalized by sequencing depth (to 10 million mapped fragments) and DESeq2 (v1.22.2) was used to calculate differential enrichment for all pairwise contrasts. Clustering was performed on the superset of differential peaks using k-means clustering by increasing k until redundant clusters arose. Gene annotations were created by assigning all intragenic peaks to that gene, and otherwise using linear genomic distance to transcription start site. The annotations in each cluster were used to intersect with the RNA-seq time series by plotting the average expression z-score of all peak-associated genes which are differentially expressed across any stage. Motif signatures and enriched pathways were obtained using Homer v4.11 (http://homer.ucsd.edu). Tracks of CUT&RUN peaks were visualized in Integrative Genomics Viewer version 2.8.9 (IGV, Broad Institute).

### scRNA-seq sample processing and analysis

Neuronal cultures at day 27 of differentiation were washed three times in PBS, incubated with Accutase supplemented with Neuron Isolation Enzyme for Pierce (Thermo 88285) solution at 1:50 at 37 °C for 45–60 min and gently dissociated to single-cell suspensions via pipetting. After washing in PBS, single-cell suspensions were diluted to 1,000 cells per μl in 1× PBS with 0.04% BSA and 0.2 U μl^−1^ Ribolock RNAse inhibitor (Thermo EO0382) for sequencing. scRNA-seq was performed at the MSKCC Integrated Genomic Core for a target recovery of 10,000 cells per sample using 10X Genomics Chromium Single Cell 3′ Kit, version 3 according to the manufacturer’s protocol. Libraries were sequenced on an Illumina NovaSeq. The CellRanger pipeline (Version 6.1.2) was used to demultiplex and align reads to the GRCh38 reference genome to generate a cell-by-gene count matrix. Data analysis was performed with R v4.1 using Seurat v4.2.0^85^. Cells expressing between 200 and 5,000 genes and less than 10% counts in mitochondrial genes were kept for analysis. Gene counts were normalized by total counts per cell and ScaleData was used to regress out cell cycle gene expression variance as determined by the CellCycleScoring function. PCA was performed on scaled data for the top 2,000 highly variable genes and a JackStraw significance test and ElbowPlot were used to determine the number of principal components for use in downstream analysis. A uniform manifold approximation and projection (UMAP) on the top 12 principal components was used for dimensional reduction and data visualization. FindNeighbors on the top 12 principal components and FindClusters with a resolution of 0.3 were used to identify clusters. Published scRNA-seq datasets for hPSC cortical differentiation were from Yao et al.^86^ (PMID: 28094016) and Volpato et al.^87^ (PMID: 30245212). To compare our dataset to those generated by Yao et al.^86^ and Volpato et al.^87^, Seurat’s anchor-based integration approach^85^ was used using FindIntegrationAnchors with 5,000 features. Single-cell hierarchical clustering and plotting for Extended Data Fig. 1h was performed with HGC^88^ using the Louvain algorithm. Single-cell RNA-seq analysis for mouse cortical development in Fig. 3f,g were from the published dataset from Di Bella et al.^41^ Data were processed using the same pipeline as in the original publication and developmental trajectories were inferred using v1.1.1.URD algorithm^89^.

### Statistics and reproducibility

Sample sizes were estimated based on previous publications in the field. Investigators were not blinded to experimental conditions. However, for knockout and small molecule treatment studies, samples were de-identified respect to the molecular target. Transcriptional and genomic studies were performed with the same bioinformatic pipeline between conditions. Statistics and data representation were performed in PRISM (GraphPad) version 8,9 or 10, excel and R software version 3.5.2 or 4.1. Statistical tests used for each analysis are indicated in the figures’ legend. Data are represented as arithmetical mean ± s.e.m. unless otherwise indicated.

Independent replication from representative micrographs were as following. Fig. 1b, 6 experiments; Fig. 1j, 3 experiments; Fig. 1n, 2 experiments, Fig. 2d, 2 experiments; Fig. 3c, 2 experiments for each genetic perturbation; Fig. 4m, 4 experiments; Supplementary Fig. 2a, 4 experiments; Supplementary Fig. 2f, 3 experiments; Supplementary Fig. 6e, 1 experiment; Extended Data Figs. 6a, 2 experiments; Extended Data Figs. 6c, 2 experiments; Supplementary Fig. 8e, 2 experiments for d12 and d16.

### Reporting summary

Further information on research design is available in the Nature Portfolio Reporting Summary linked to this article.

## Online content

Any methods, additional references, Nature Portfolio reporting summaries, source data, extended data, supplementary information, acknowledgements, peer review information; details of author contributions and competing interests; and statements of data and code availability are available at 10.1038/s41586-023-06984-8.

### Supplementary information

Supplementary Information This file contains Supplementary Figs. 1–9 and Supplementary Tables 1–5.

Reporting Summary

Supplementary Video 1

Supplementary Video 2

Supplementary Video 3

Supplementary Video 4

Supplementary Video 5

Supplementary Video 6

Supplementary Video 7

Supplementary Video 8

### Source data

Source Data Fig. 1

Source Data Fig. 2

Source Data Fig. 3

Source Data Fig. 4

Source Data Fig. 5

Source Data Extended Data Fig. 1

Source Data Extended Data Fig. 2

Source Data Extended Data Fig. 3

Source Data Extended Data Fig. 4

Source Data Extended Data Fig. 5

Source Data Extended Data Fig. 6

Source Data Extended Data Fig. 7

Source Data Extended Data Fig. 9

Source Data Extended Data Fig. 10

## Data Availability

All genomic datasets have been deposited at GEO under accession numbers GSE196075, GSE196109 and GSE226223. Publicly available datasets of human brain development were from BrainSpan atlas of the developing human brain (https://www.brainspan.org/static/download.html), the genome assembly GRCh38 (hg19) (https://www.ncbi.nlm.nih.gov/datasets/genome/GCF_000001405.13/) and the GRCh38 genome assembly (hg38) (https://www.ncbi.nlm.nih.gov/datasets/genome/GCF_000001405.26/). Published datasets of mouse cortical development^41^, and hPSC cortical differentiations^86,87^ were re-analysed. Source data are provided with this paper.
