# Supplementary material for: An epigenetic barrier sets the timing of human neuronal maturation
Source: Nature. 2024 Jan 31;626(8000):881–90. doi: 10.1038/s41586-023-06984-8 (PMC10881400; doi:10.1038/s41586-023-06984-8)
Supplement: Supplementary file 1 — This file contains Supplementary Figs. 1–9 and Supplementary Tables 1–5. [file 41586_2023_6984_MOESM1_ESM.pdf]

nature portfolio

https://doi.org/10.1038/s41586-023-06984-8

Supplementary information

An epigenetic barrier sets the timing of human neuronal maturation

In the format provided by the authors and unedited

An epigenetic barrier sets the timing of human neuronal maturation

Gabriele Ciceri[1]*, Arianna Baggiolini[1,7], Hyein S. Cho[2], Meghana Kshirsagar [2,8], Silvia Benito-Kwiecinski[1], Ryan M Walsh[1], Kelly A Aromolaran[3], Alberto J. Gonzalez-Hernandez[4], Hermany Munguba[4], So Yeon Koo[1,6], Nan Xu[1], Kaylin J. Sevilla[1], Peter A Goldstein[3], Joshua Levitz[4], Christina S Leslie[2,9], Richard P. Koche[5,9], Lorenz Studer[1]*

Affiliations:

[1] The Center for Stem Cell Biology and Developmental Biology program; Memorial Sloan Kettering Cancer Center, New York, NY, USA

[2] Computational Biology Program, Memorial Sloan Kettering Cancer Center; New York, NY, USA

[3] Department of Anesthesiology, Weill Cornell Medicine; New York, NY, USA

[4] Department of Biochemistry, Weill Cornell Medicine, New York, NY, USA

[5] Center for Epigenetics Research, Memorial Sloan Kettering Cancer Center; New York, NY, USA

[6] Weill Cornell Neuroscience PhD program, New York, NY, USA

[7] Present address: Institute of Oncology Research (IOR), BIOS+, Bellinzona 6500, Switzerland & Università della Svizzera Italiana, Lugano 6900, Switzerland.

[8] Present address: Microsoft AI for Good research; Redmond, WA, USA

[9] These authors contributed equally to these work

*Corresponding authors. Email: studerl@mskcc.org; cicerig@mskcc.org

SI guide

Supplementary Figure 1. Uncropped images for Western blots page 1

Supplementary Figure 2. Synchronized neurogenesis and long-term maintenance of cortical neurons generated from hPSCs page 5

Supplementary Figure 3. Gene ontology and BrainSpan comparison for maturation -dependent upregulated transcripts page 6

Supplementary Figure 4. Motif analysis for unbiassed ATACseq clusters and pairwise comparisons of chromatin accessibility during neuronal maturation page 7

Supplementary Figure 5. Gene ontology and BrainSpan comparison for maturation dependent downregulated transcripts page 8

Supplementary Figure 6. Experimental strategy for gene knock-out in hPSCs-derived neurons page 9

Supplementary Figure 7. Representative WB and additional Ca^{2+} imaging analysis for the loss-of-function of epigenetic factors in neurons page 10

Supplementary Figure 8. Generation of cortical neurons from mouse PSC page 11

Supplementary Figure 9. Generation of CHD5 and JADE2 KO hPSC lines page12

Supplementary Table 1. Electrophysiological properties of maturing hPSC-derived cortical neurons page 13

Supplementary Table 2. Additional statistical information of western blot analysis in loss-of-function experiments (Fig. 3d) page 14

Supplementary Table 3. Additional statistical information of Ca^{2+} imaging analysis in loss-of-function experiments (Fig. 3e) page 15

Supplementary Table 4. Sequence of customized qRT-PCR primers page 16

Supplementary Table 5. List of gRNA sequences used for gene KO in hPSC-derived neurons page 17

Supplementary Videos:

Supplementary Video 1. **Representative Ca^{2+} imaging for hPSC-derived cortical neurons at d40.** WA09 hPSC-derived cortical neurons were infected at d25 with lentiviruses encoding GCaMP6m and imaged at d40 of differentiation. Fluorescence was recorded every ~ 200 ms. Video displays 800 frames of imaging at 20 frames/second in one field of view.

Supplementary Video 2. **Representative Ca^{2+} imaging for hPSC-derived cortical neurons at d70.** WA09 hPSC-derived cortical neurons were infected at d25 with lentiviruses encoding GCaMP6m and imaged at d70 of differentiation. Fluorescence was recorded every ~ 200 ms. Video displays 800 frames of imaging at 20 frames/second in one field of view.

Supplementary Video 3. Representative Ca^{2+} imaging for hPSC-derived cortical neurons derived from control (DMSO) treated NPC. WA09 hPSC-derived cortical NPC were transiently treated with DMSO (0.04%) from d12 to d20. Neurons were infected at d25 with lentiviruses encoding GCaMP6m and imaged at d40 of differentiation. Fluorescence was recorded every ~ 200 ms. Video displays 800 frames of imaging at 20 frames/second in one field of view.

Supplementary Video 4. **Representative Ca^{2+} imaging for hPSC-derived cortical neurons derived from NPC treated with the EZH2 inhibitor.** WA09 hPSC-derived cortical NPC were transiently treated with GSK343 (4μM) from d12 to d20. Neurons were infected at d25 with lentiviruses encoding GCaMP6m and imaged at d40 of differentiation. Fluorescence was recorded every ~ 200 ms. Video displays 800 frames of imaging at 20 frames/second in one field of view.

Supplementary Video 5. Representative Ca^{2+} imaging for hPSC-derived cortical neurons derived from NPC treated with the EHMT1/2 inhibitor. WA09 hPSC-derived cortical NPC were transiently treated with UNC0638 (4μM) from d12 to d20. Neurons were infected at d25 with lentiviruses encoding GCaMP6m and imaged at d40 of differentiation. Fluorescence was recorded every ~ 200 ms. Video displays 800 frames of imaging at 20 frames/second in one field of view.

Supplementary Video 6. **Representative Ca^{2+} imaging for hPSC-derived cortical neurons derived from NPC treated with the DOT1L inhibitor.** WA09 hPSC-derived cortical NPC were transiently treated with EPZ004777 (4μM) from d12 to d20. Neurons were infected at d25 with lentiviruses encoding GCaMP6m and imaged at d40 of differentiation. Fluorescence was recorded every ~ 200 ms. Video displays 800 frames of imaging at 20 frames/second in one field of view.

Supplementary Video 7. **Representative Ca^{2+} imaging of hPSC-derived cortical control organoids transiently treated with DMSO.** WA09 hPSC-derived cortical organoids were treated with 0.04% DMSO from d17 to d25 of differentiation, infected with lentiviruses encoding GCaMP6m at day45 and processed for light-sheet imaging at d55 of differentiation. Fluorescence was recorded at 5 frames/second. Video displays 600 frames of imaging at 100 frames/second in one field of view.

Supplementary Video 8. Representative Ca^{2+} imaging of hPSC-derived cortical organoids transiently treated with the EZH2 inhibitor. WA09 hPSC-derived cortical organoids were treated with GSK343 (4μM) from d17 to d25 of differentiation, infected with lentiviruses encoding GCaMP6m at day45 and processed for light-sheet imaging at d55 of differentiation. Fluorescence was recoded at 5 frames/second. Video displays 600 frames of imaging at 100 frames/second in one field of view.

From Extended Data Fig. 4a

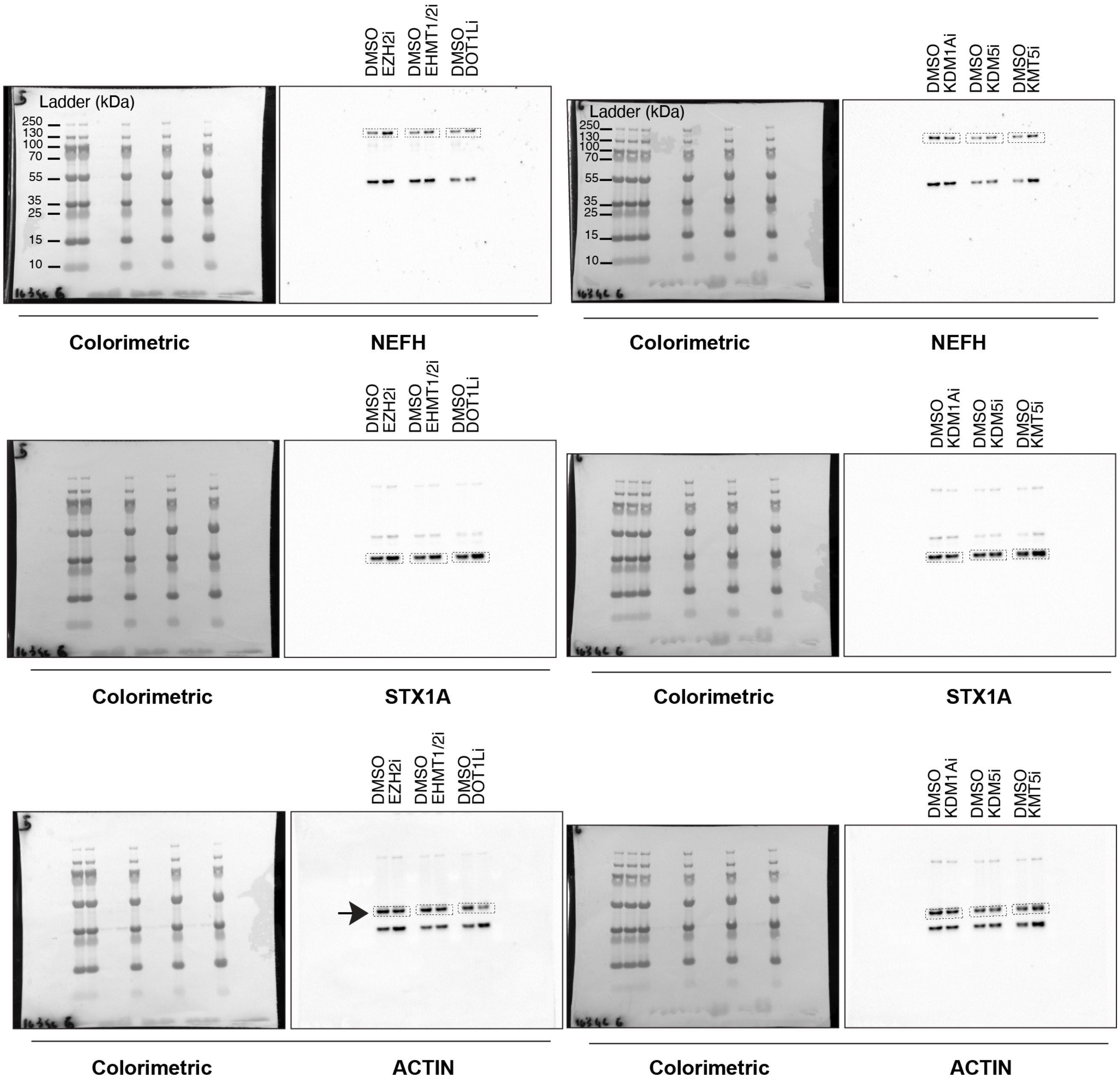

From Supplementary Figure 6

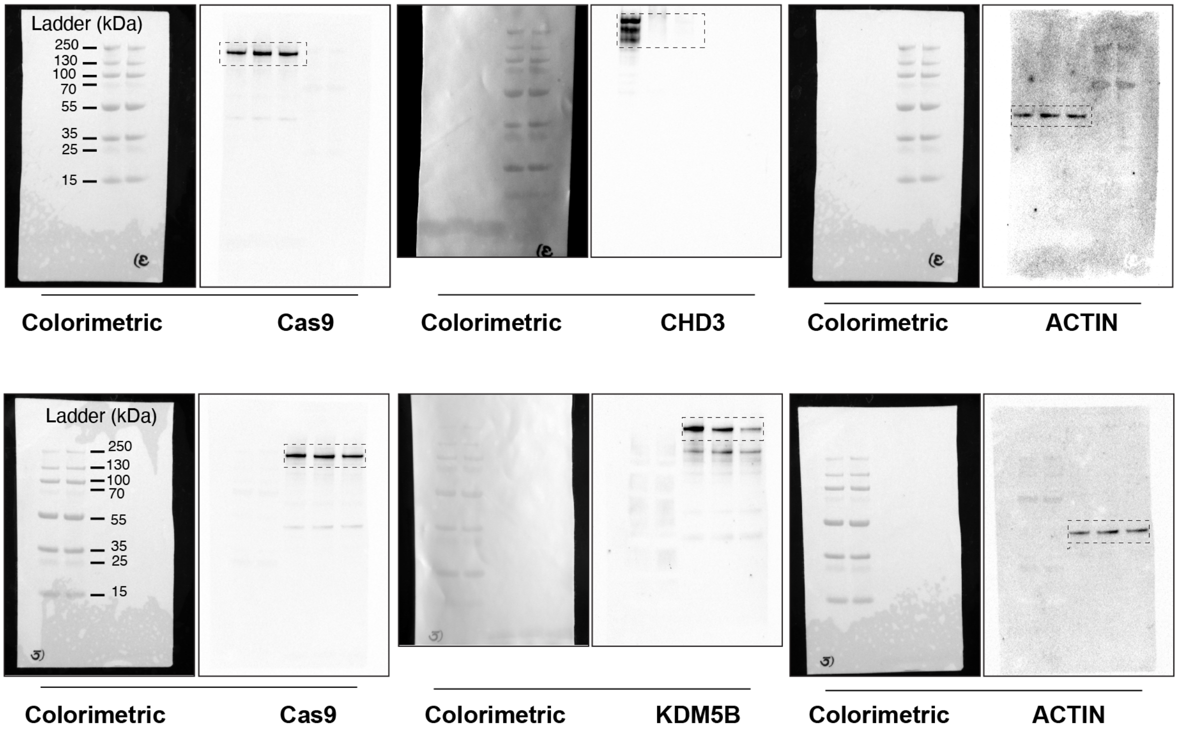

From Supplementary Figure 7 (top left)

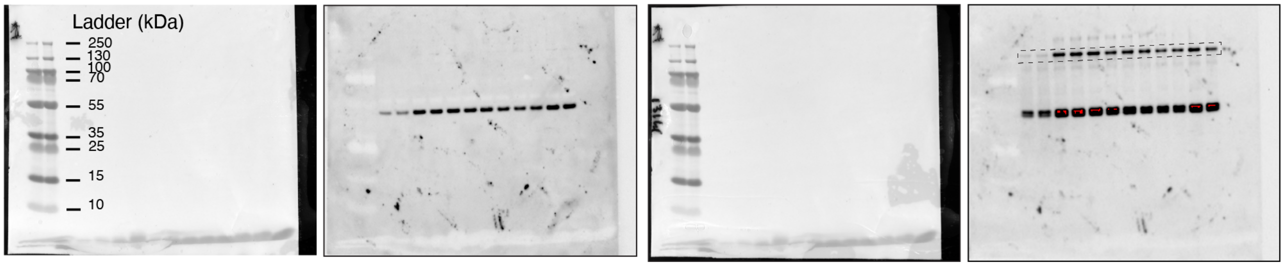

Colorimetric | Homer1 (not shown) | Colorimetric | NEFH

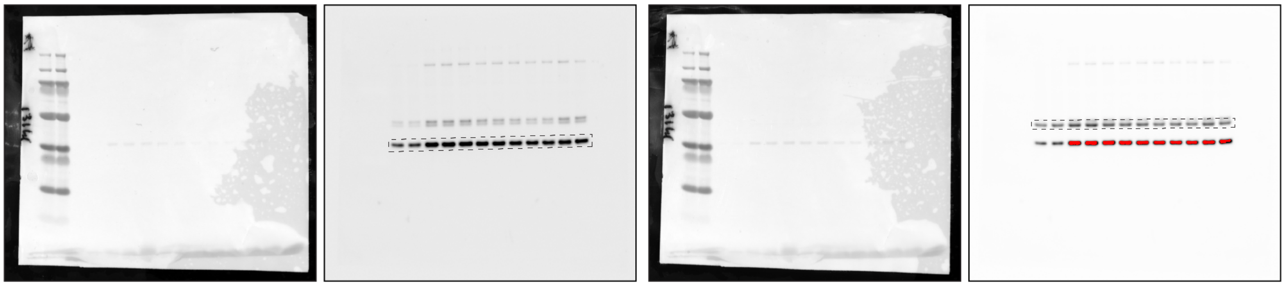

Colorimetric | STX1A | Colorimetric | ACTIN

From Supplementary Figure 7 (top right)

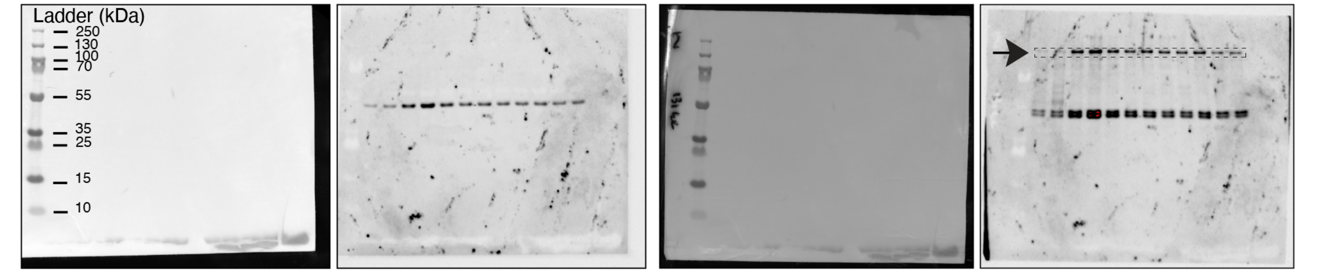

Colorimetric | Homer1 (not shown) | Colorimetric | NEFH

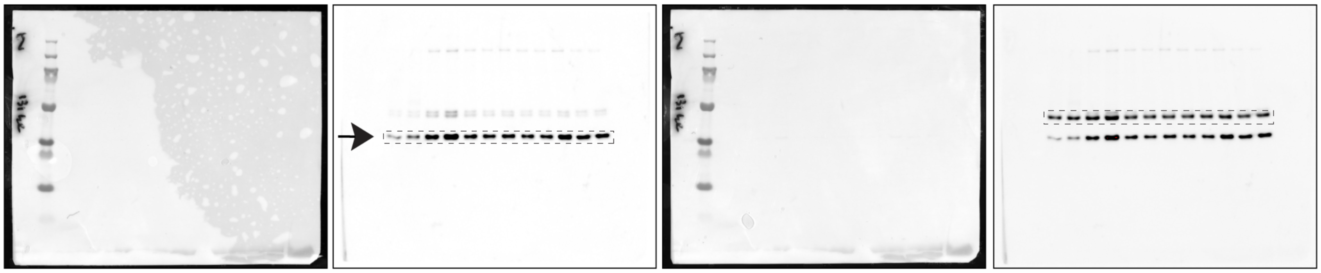

Colorimetric | STX1A | Colorimetric | ACTIN

From Supplementary Figure 7 (bottom left)

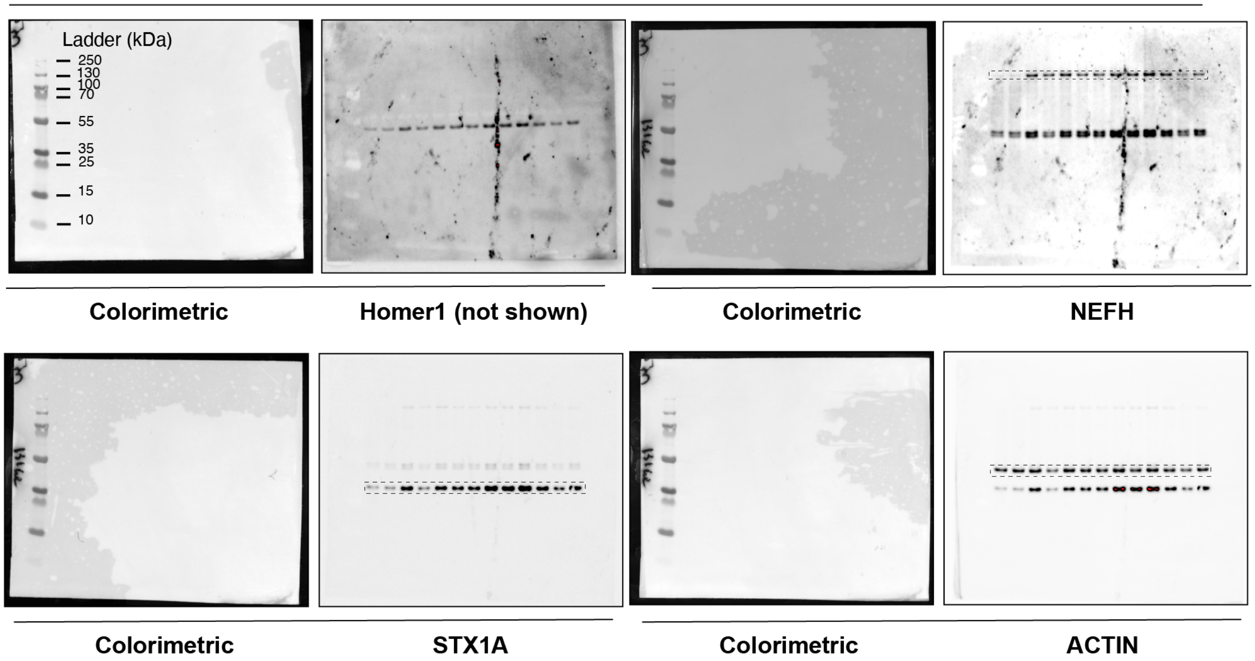

From Supplementary Figure 7 (bottom right)

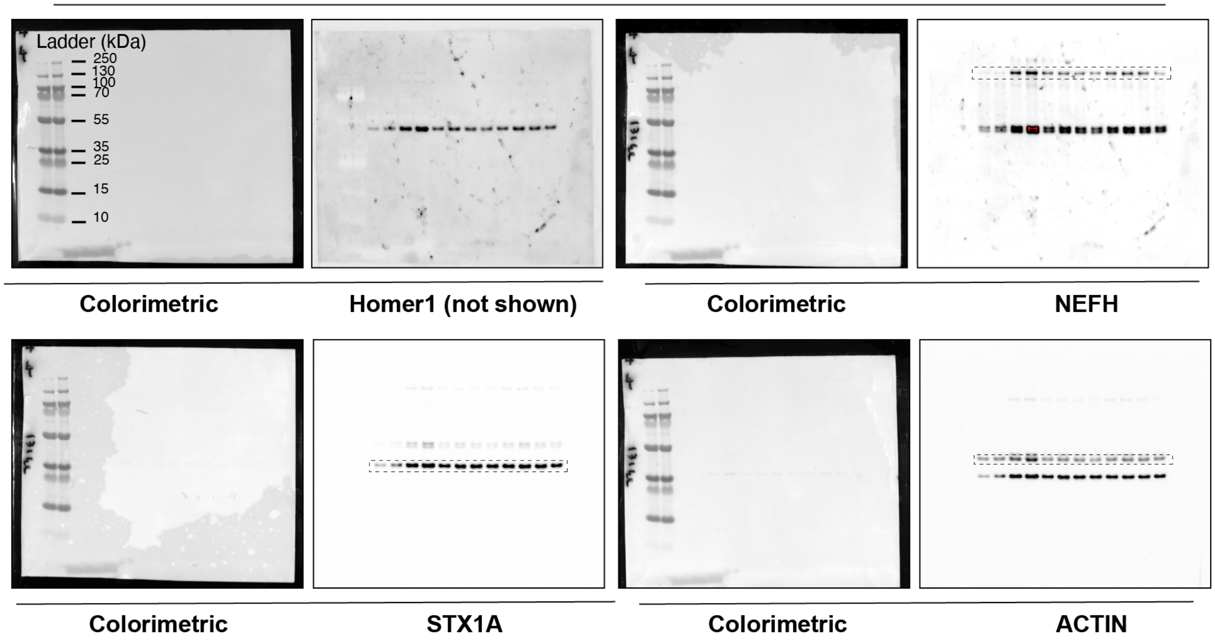

Supplementary Figure 1. **Uncropped images for Western blots**

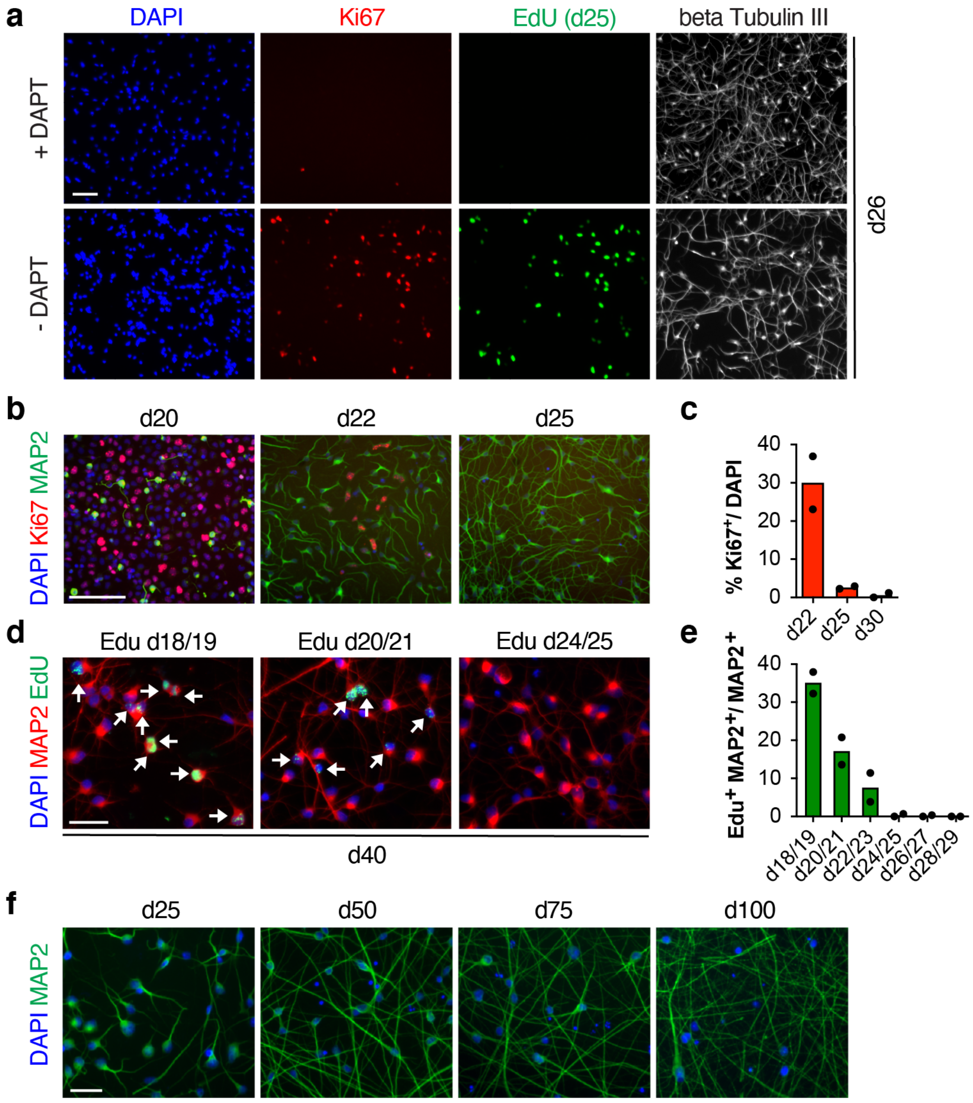

Supplementary Figure 2. Synchronized neurogenesis and long-term maintenance of cortical neurons generated from hPSCs. a, Cell passaging at low density and DAPT treatment rapidly deplete the pool of progenitor cells. Cells were culture in presence or absence of DAPT from d20, pulse labelled with EdU for 24h at d25 and analyzed at d26 by immunostaining for EdU, KI67 and MAP2. **b-c**, Representative images (b) and quantification (c) of percentage of MKI67^{+} NPC and MAP2^{+} neurons after the induction of synchronized neurogenesis at d20 (n = 2 independent experiments). **d-e**, Representative images (d) and quantification (e) of the fraction of d40 MAP2 neurons that were labelled by EdU pulses of progenitor cells at the indicated days (n = 2 independent experiments). **f**, Synchronized cortical neurons can be maintained at high viability in long-term cultures. Representative images of cortical neurons stained with antibody against MAP2 at day25, 50, 75 and 100 of differentiation. Histograms depict mean. Scale bars are 100 µm (a,b); 50 µm (d,f).

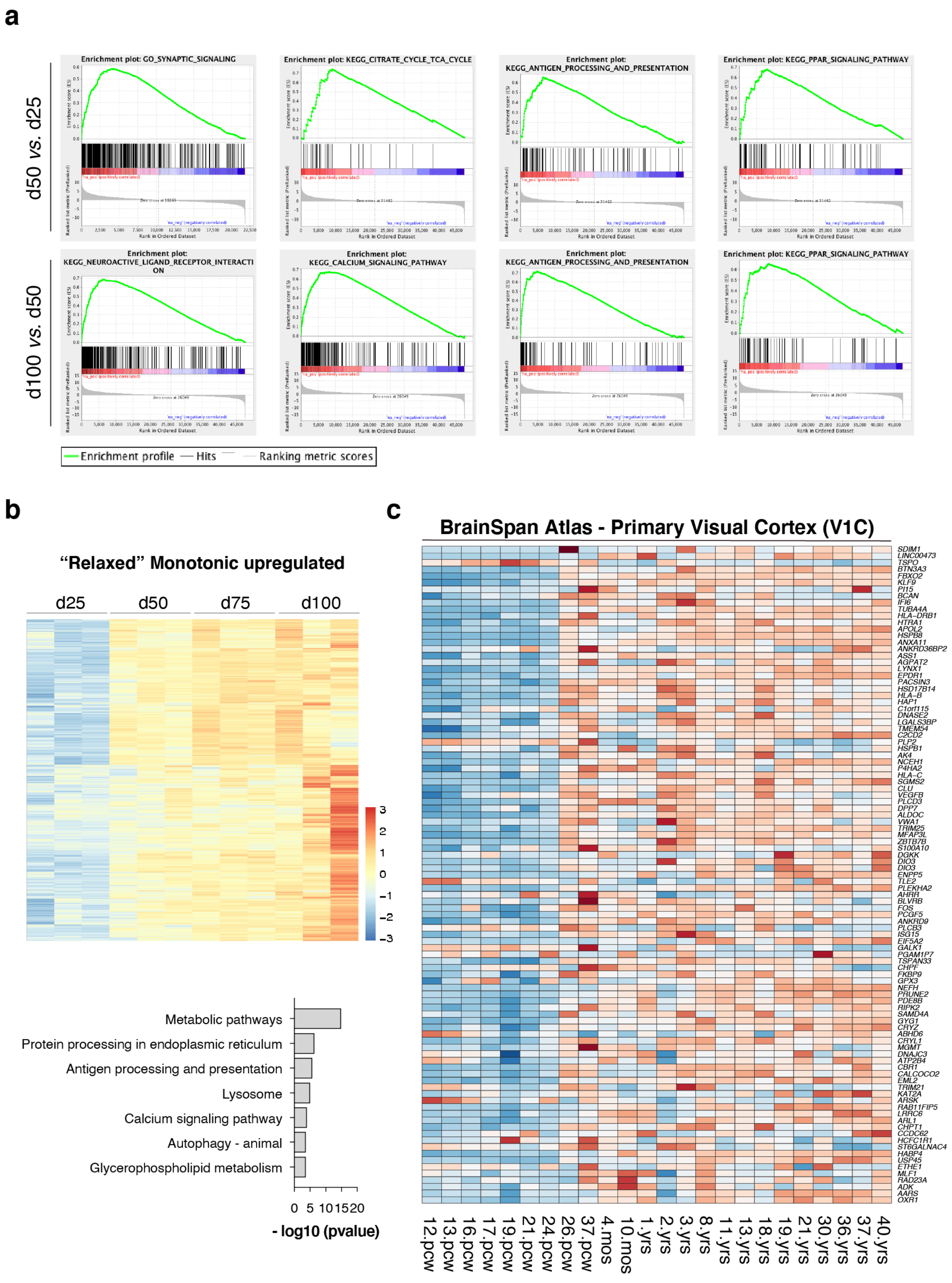

Supplementary Figure 3. Gene ontology and BrainSpan comparison for maturation dependent upregulated transcripts. a, GSEA plots for some of the GO terms from RNAseq studies (n = 3 independent experiments) that positively correlate with neuronal maturation in d50 *vs.* d25 and d100 *vs.* d50 pairwise comparisons. **b,** Heatmap for the normalized temporal expression of "relaxed" monotonically upregulated transcripts during neuronal maturation (top) and GO for KEGG pathways (bottom). **c,** Heatmap for the normalized temporal expression of the corresponding monotonically upregulated transcripts in the BrainSpan atlas of the developing human brain (primary visual cortex) shown in Fig. 2c. Fisher's Exact test (b).

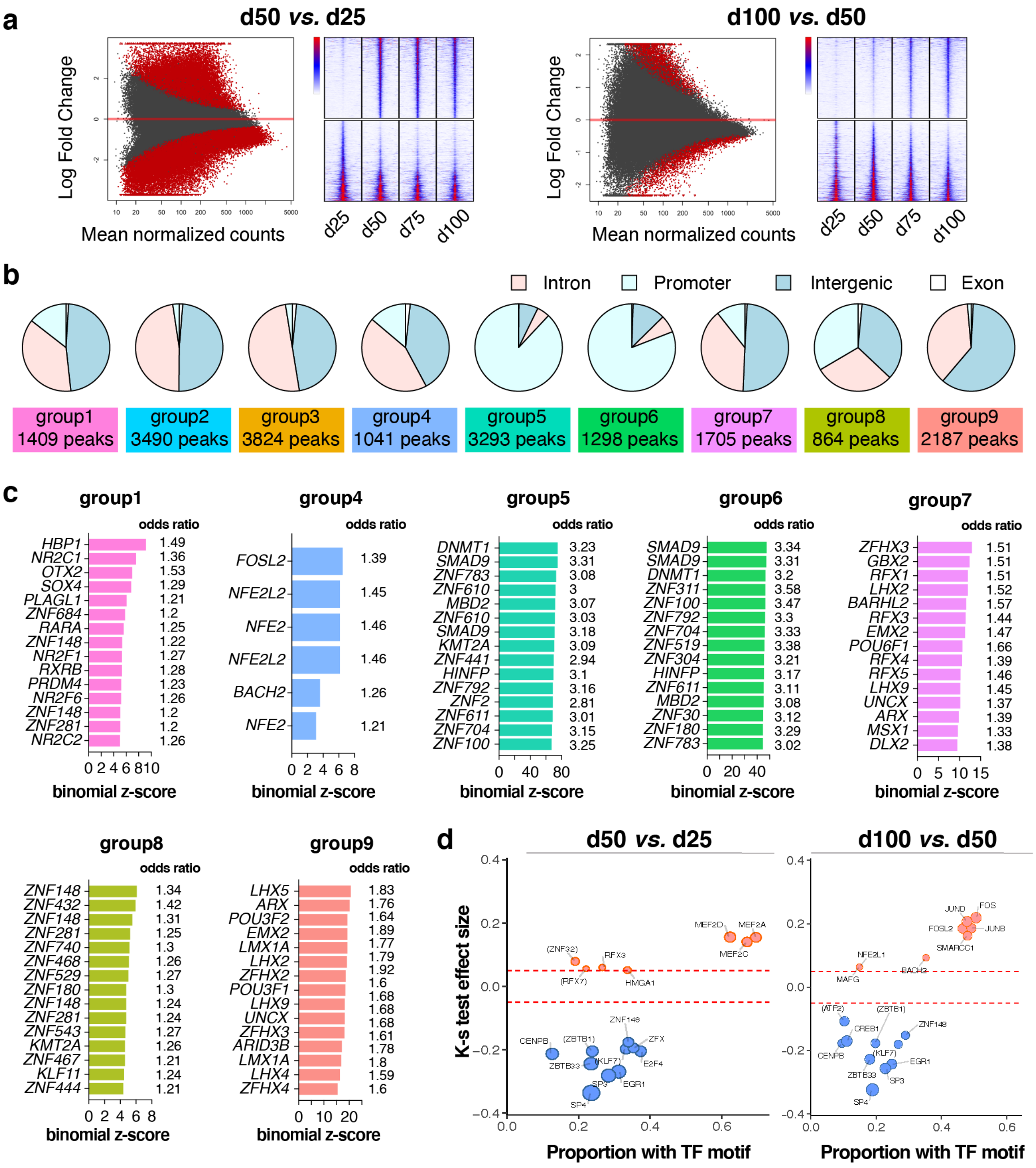

Supplementary Figure 4. Motif analysis for unbiassed ATACseq clusters and pairwise comparisons of chromatin accessibility during neuronal maturation. **a,** MA (left) and tornado plots (right) for differentially accessible ATACseq peaks in d25 *vs.* d50 and d50 *vs.* d100 pairwise comparisons. **b**, Pie charts of ATACseq peaks mapped to gene promoters, introns, exons, and intergenic genomic regions for each of the cluster in Fig. 2f. **c**, Top transcription factor motifs enriched by the hypergeometric test in the indicated groups of ATACseq peaks. Odds ratio indicates the normalized enrichment of transcription factor motifs in the cluster compared to the background. **d,** Top transcription factor motifs enriched in differentially accessible ATACseq peaks in d50 *vs.* d25 and d100 *vs.* d50 pairwise comparisons. (a-d) Analysis derived from ATACseq dataset originated from n = 2 independent experiments.

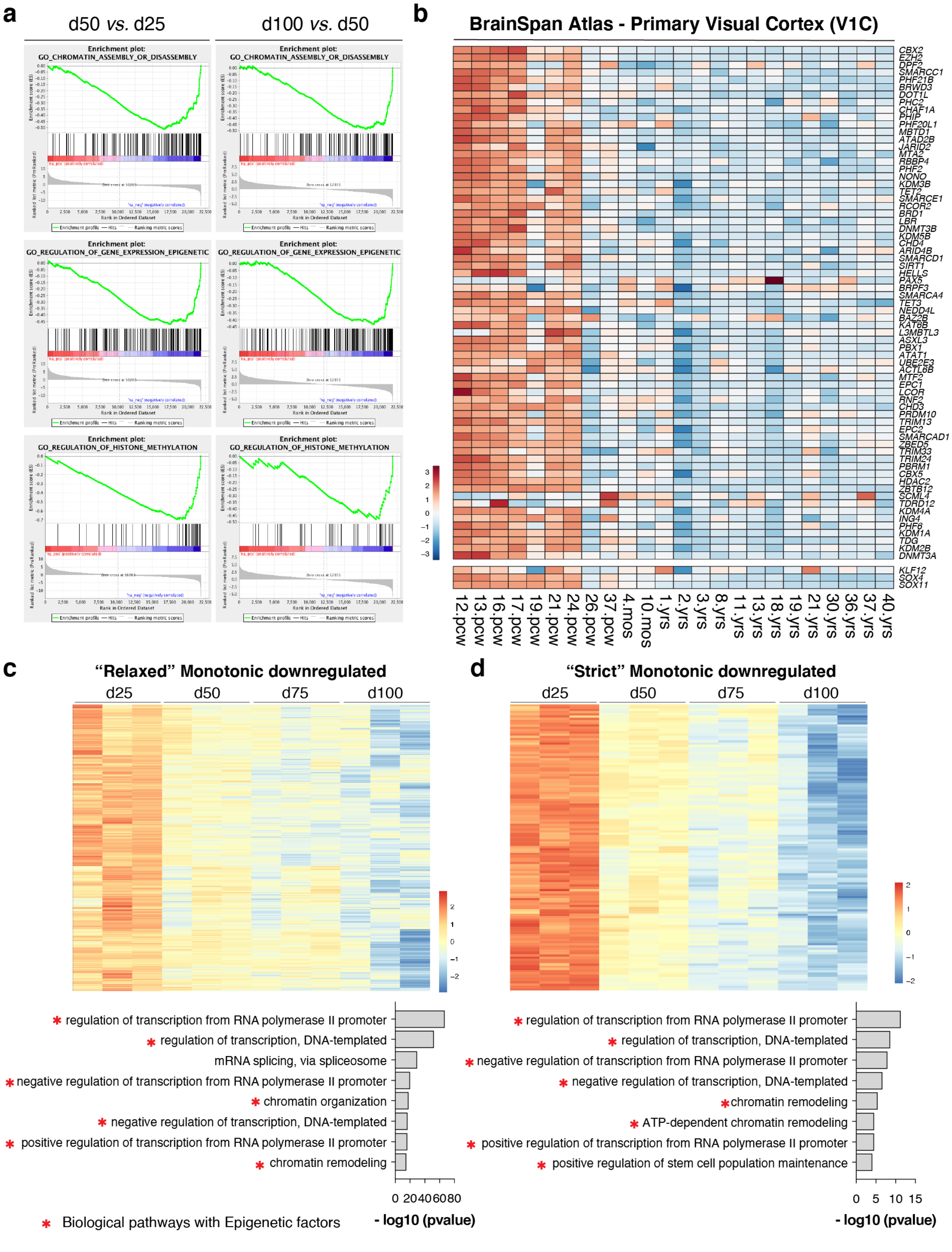

Supplementary Figure 5. Gene ontology and BrainSpan comparison for maturation dependent downregulated transcripts. a, GSEA plots for some of the GO terms from RNAseq studies (n = 3 independent experiments) related to chromatin remodeling in d50 *vs.* d25 and d100 *vs.* d50 pairwise comparisons. **b,** Heatmap for the normalized temporal expression of the corresponding monotonically downregulated chromatin regulators in the BrainSpan atlas of the developing human brain (primary visual cortex) shown in Fig. 3b. **c,** Heatmap for the normalized temporal expression of "relaxed" monotonically downregulated transcripts during neuronal maturation (top) and GO for biological pathways (bottom). **d,** Heatmap for the normalized temporal expression of "strict" monotonically downregulated transcripts during neuronal maturation (top) and GO for biological pathways (bottom). Fisher's Exact test (c, d).

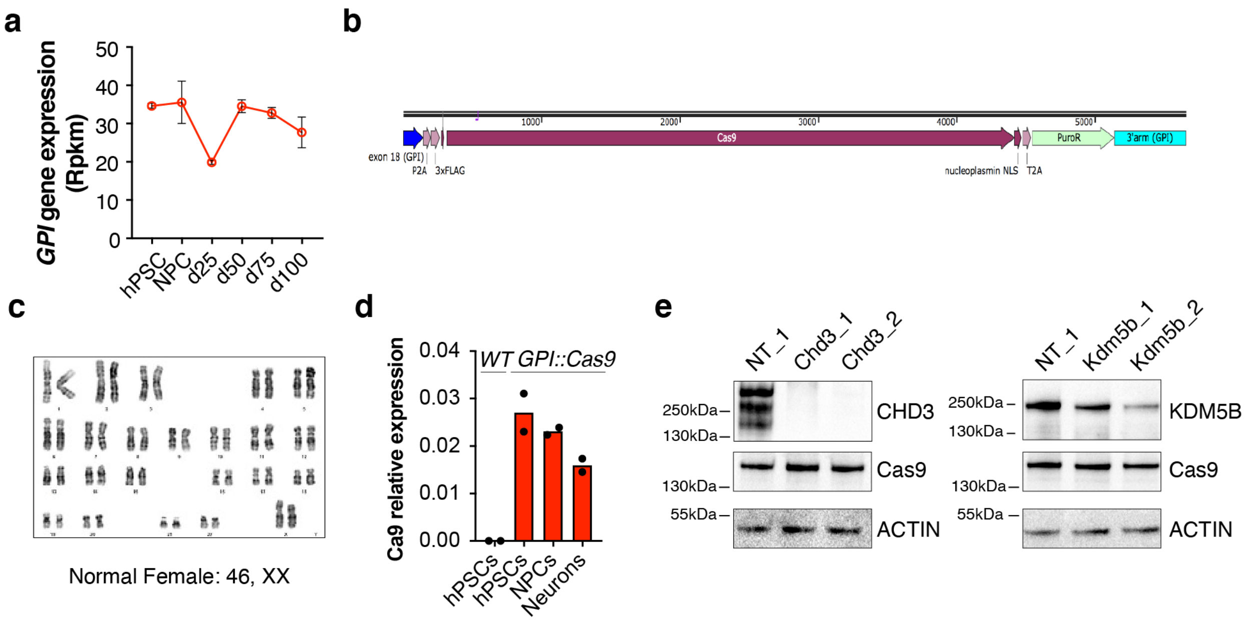

Supplementary Figure 6. Experimental strategy for gene knock-out in hPSCs-derived neurons. a, Expression of *GPI* gene throughout the differentiation (n = 3 independent experiments). **b,** Schematic for the targeting construct used for the generation of the GPI::Cas9 knock-in hPSCs line. Cas9 is linked to the GPI gene via 2A self-cleaving peptide sequence. **c,** Karyotypic analysis of the GPI::Cas9 hPSCs clonal cell line used for the study. **d,** Expression of Cas9 mRNA in the GPI::Cas9 line at hPSC, NPC and neuron stages compared to wild type hPSC (n = 2 independent experiments). **e,** Representative Western Blot analysis for CRISPR/Cas9-based gene KO for CHD3 and KDM5B in neurons using the same strategy shown in Fig. 3c. Cas9 expressing neurons at d25 were infected with lentiviral vectors encoding non-targeting and gene-specific gRNAs and analyzed at day35 of differentiation. Data is depicted as mean ± s.e.m.

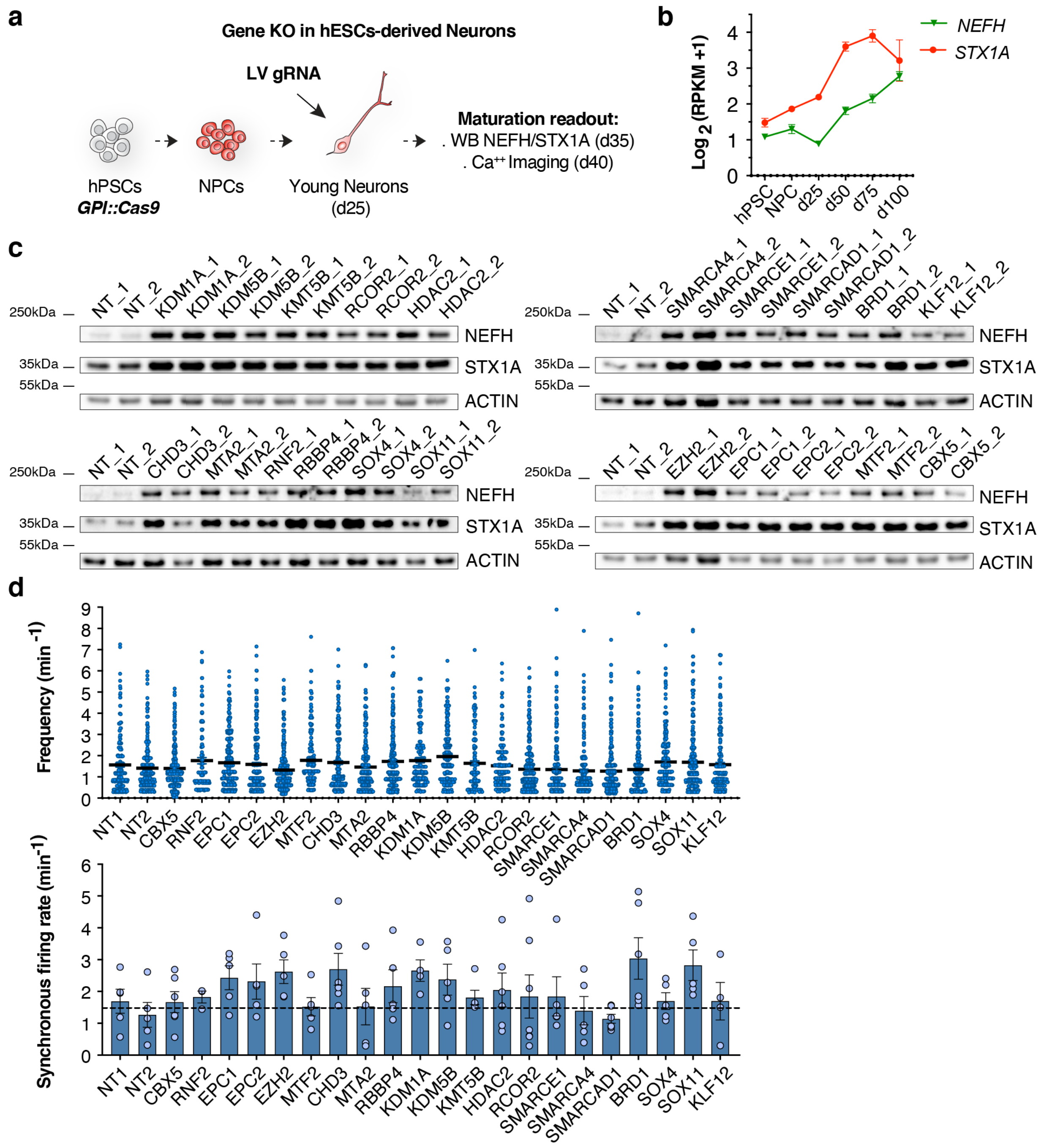

Supplementary Figure 7. Representative WB and additional Ca^{2+} imaging analysis for the loss-of-function of epigenetic factors in neurons. a, Schematic of experimental paradigm for gene KO in hPSCs-derived neurons. **b,** Gene expression for NEFH and STX1A maturation markers throughout the differentiation (n = 3 independent experiments). **c,** Representative Western Blots for the loss-of-function genetic screen of chromatin regulators. Quantification is shown in Fig. 3d. Gels/blots from the same experiments were processed in parallel. **d,** Frequency of spontaneous Ca^{2+} spikes of individual neurons (top) and synchronicity rate of spontaneous network activity in loss-of-function experiments. n are reported in Supplementary Table 3. Synchronous firing rate: NT1, NT1, EPC1, EPC2, EZH2, KDM5B, KMT5B, MTA2, MTF2, RBBP4, SMARCA4, SMARCE1, SMARCAD1, n = 5; BRD1, CBX5, CHD3, HDAC2, n = 6; RNF2, n = 3; KDM1A, KLF12, n = 4, RCOR2, n = 7 (FOV from 2 independent experiments). Histograms and lines depict mean ± s.e.m. Welch's one-way ANOVA with Games-Howell's multiple comparisons test (d, frequency); Welch's one-way ANOVA with Dunnett's multiple comparison test (d, Synchronicity). FOV, field of view.

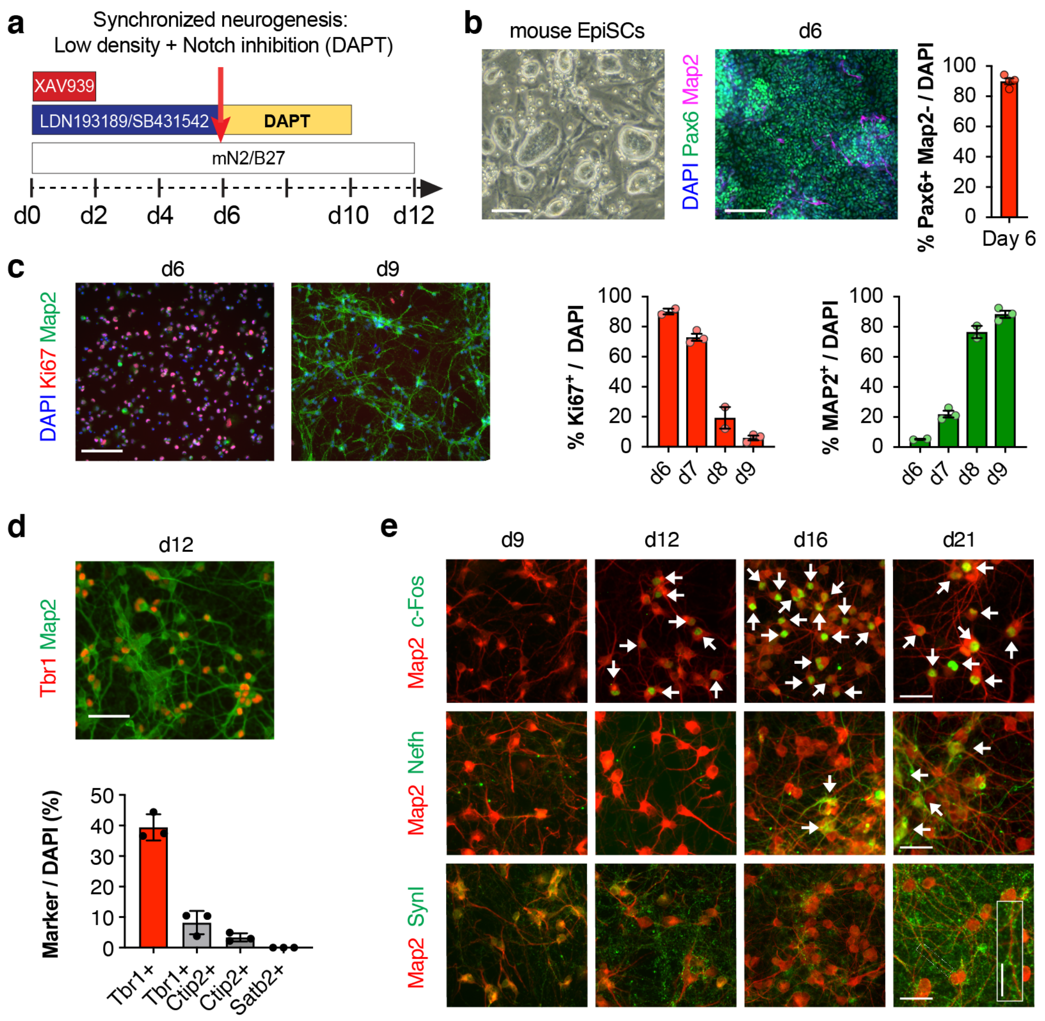

Supplementary Figure 8. Generation of cortical neurons from mouse PSC. a, Schematics of the differentiation protocol of mouse epiblast stem cells (mEpiSC) based on dual-SMAD and WNT inhibition. The panel indicate differentiation days, basal media, and small molecules treatments. The red arrow indicates cell-passaging at low density in presence of notch pathway inhibitor DAPT. **b,** Representative images of mEpiSC colonies using MEF feeders (left) and cultures at d6 stained with the forebrain progenitor cell marker Pax6 and the neuron marker MAP2 (middle). Quantification of the fraction of cells expressing Pax6 is shown on the right (n = 4 independent experiments). **c,** Representative images (left) and quantification (right) of the percentage of $Mki67^{+}$ NPC and $Map2^{+}$ neurons after the induction of synchronized neurogenesis at d6. d6 n = 2, d7 n = 3, d8 n = 2, d9 n = 3 (independent experiments). **d,** Representative images of neuronal cultures stained for Map2 and Tbr1 (top) and quantification (bottom) of the fraction of neurons generated through synchronized neurogenesis and expressing Tbr1, Ctip2 and Satb2 neuron markers (n = 3 independent experiments). **e,** Representative images of neurons at indicated time-points stained with antibodies for indicated maturation markers. Histograms depict mean ± s.e.m. Scale bars are 100 µm (b, c); 50 µm (d, e).

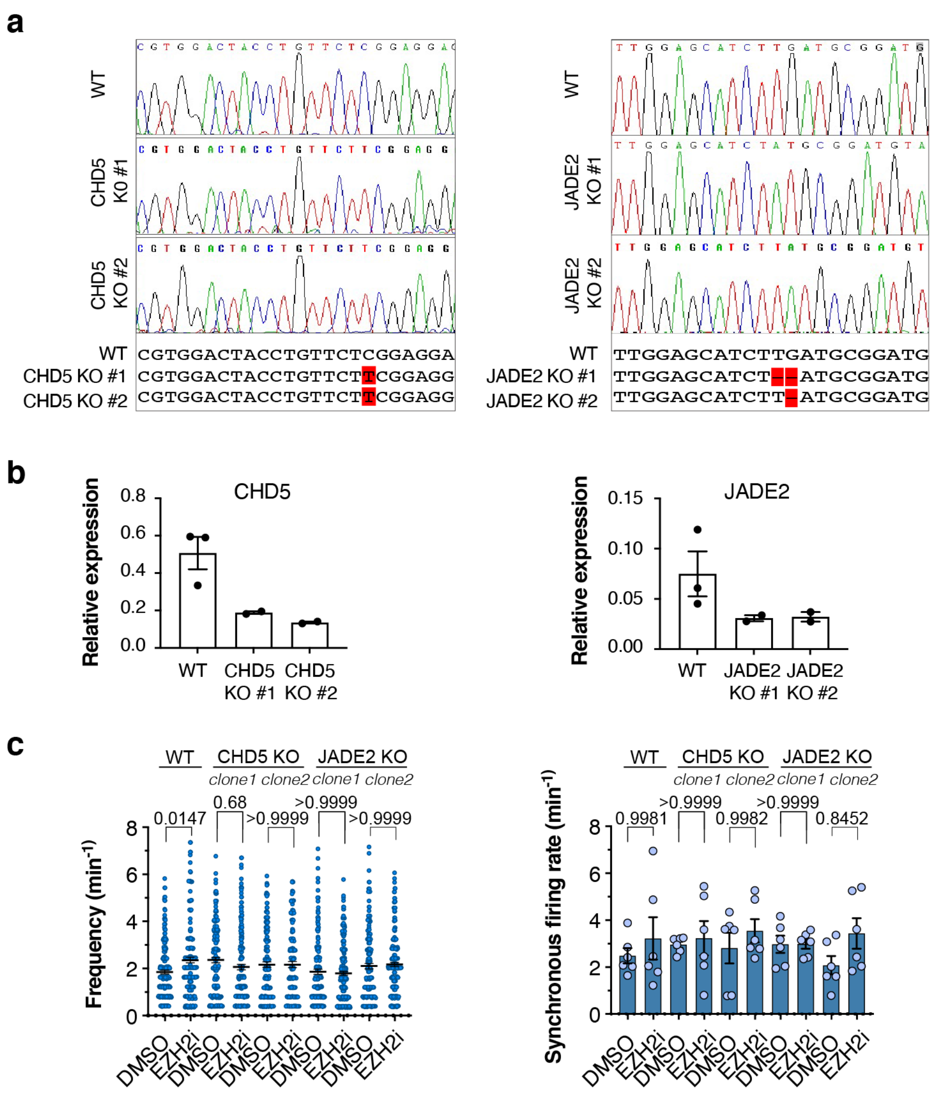

Supplementary Figure 9. Generation of CHD5 and JADE2 KO hPSC lines: a, Sanger sequencing for the verification of indel mutations into the genomic sequence of CHD5 and JADE2 genes following CRISPR/Cas9 genomic targeting. Red boxes identify indels. **b,** qRT-PCR expression of *CHD5* and *JADE2* in hPSC-derived neurons at d35 of differentiation. Left: WT n = 3, CHD5 KO#1 and #2 n = 2; Right: WT n = 3, JADE1 KO#1 and #2 n = 2 (independent experiments). **c,** Frequency of spontaneous Ca^{2+} spikes of individual neurons (left) and synchronicity rate of spontaneous network activity (right) in hPSC-derived neurons from indicated genotype at d40 of differentiation. Frequency. DMSO: n = 241 (WT), n= 184 (CHD5-KO#1), n= 165 (CHD5-KO#2), n= 183 (JADE2-KO#1), n= 171 (JADE2-KO#2); EZH2i n = 190 (WT), n = 197 (CHD5-KO#1), n = 147 (CHD5-KO#2), n = 222 (JADE2-KO#1), n = 213 (JADE2-KO#2) neurons from 2 independent experiments. Synchronous firing rate. n = 6 field of view from 2 independent experiments. Welch's one-way ANOVA with Games-Howell's correction (c, left); ordinary one-way ANOVA with Dunnett's multiple comparison test (c, right). Histograms and lines depict mean ± s.e.m.

	day 25	day 50	day 75	day 100
**RMP (mV)**	-36.7±2.0	-50.2±1.2	-52.3±1.1	-51.6±1.4
**IR (MΩ)**	1,601.5±100.9	731.4±53.3	646.0±48.6	518.5±65.9
**Rheobase (pA)**	98.8±8.5	32.7±6.5	36.0±4.7	29.7±4.5
**APT (mV)**	-8.8±2.4	-31.5±1.2	-30.1±1.0	-34.5±1.0
**APA (mV)**	25.1±1.3	48.2±3.1	53.1±1.8	59.1±2.3
**APD (ms)**	23.9±2.1	14.1±1.4	10.0±1.0	9.5±0.7
**AHP (ms)**	12.0±1.2	6.0±0.7	4.0±0.3	3.5±0.2
**Rise time (ms)**	5.5±0.5	2.5±0.3	1.7±0.2	1.6±0.2
**Decay time (ms)**	9.6±0.9	7.3±1.0	4.3±0.5	4.1±0.3
**Rise slope (mV/ms)**	4.4±0.8	29.7±4.3	39.8±4.0	42.4±4.3
**Decay slope (mV/ms)**	-2.9±0.7	-9.9±1.4	-14.2±1.3	-13.7±1.1
**Cm (pF)**	21.2±1.6	28.3±1.9	35.2±2.0	31.6±1.9
**Rm (MΩ)**	505.2±61.6	192.0±20.0	144.8±8.7	143.6±13.5
**Access resistance (MΩ)**	39.8±2.6	42.9±2.3	36.5±1.9	39.6±1.7
**n**	25	33	43	29

Supplementary Table 1. Electrophysiological properties of maturing hPSC-derived cortical neurons. Quantification of the electrophysiological properties of hPSC-derived neurons at day 25, 50, 75, 100 of differentiation. Results are displayed as mean ± s.e.m. RMP, Resting membrane potential; IR, Input resistance, APT, Action potential threshold; APA, Action potential amplitude; APD, Action potential duration at its half amplitude; AHP, After hyperpolarization amplitude; Cm, Membrane capacitance; Rm, Membrane resistance.

Gene Target	P value (NEFH)	t-value; df (NEFH)	gRNA_1 n	gRNA_2 n	Alpha	P value (STX1A)	t-value; df (STX1A)	gRNA_1 n	gRNA_2 n	Alpha
CBX5	0.0016	t=6.238, df=5	3	3	0.05	0.019	t=3.410, df=5	3	3	0.05
RNF2	0.0043	t=4.452, df=6	3	4	0.05	0.0008	t=6.168, df=6	3	4	0.05
EPC1	0.0042	t=4.160, df=7	4	4	0.05	0.0821	t=2.309, df=4	2	3	0.05
EPC2	0.0007	t=5.766, df=7	4	4	0.05	0.0121	t=3.844, df=5	3	3	0.05
EZH2	0.0019	t=4.833, df=7	4	4	0.05	0.0003	t=7.295, df=6	4	3	0.05
MTF2	0.0034	t=4.339, df=7	4	4	0.05	0.0036	t=5.165, df=5	3	3	0.05
CHD3	0.006	t=4.565, df=5	3	3	0.05	0.0025	t=5.581, df=5	3	3	0.05
MTA2	0.0003	t=9.005, df=5	3	3	0.05	<0.0001	t=13.75, df=5	3	3	0.05
RBBP4	0.0014	t=6.379, df=5	3	3	0.05	0.0017	t=6.107, df=5	3	3	0.05
KDM1A	0.0098	t=4.048, df=5	3	3	0.05	0.0009	t=5.137, df=8	5	4	0.05
KDM5B	0.0156	t=3.598, df=5	3	3	0.05	<0.0001	t=13.39, df=6	3	4	0.05
KMT5B	0.007	t=4.406, df=5	3	3	0.05	0.0003	t=7.380, df=6	3	4	0.05
HDAC2	0.0061	t=4.560, df=5	3	3	0.05	0.0102	t=3.687, df=6	3	4	0.05
RCOR2	0.0551	t=3.058, df=3	2	2	0.05	0.0606	t=2.413, df=5	3	3	0.05
SMARCE1	<0.0001	t=43.44, df=3	2	2	0.05	0.0076	t=4.976, df=4	3	2	0.05
SMARCA4	<0.0001	t=32.47, df=3	2	2	0.05	0.0118	t=4.393, df=4	2	3	0.05
SMARCAD1	<0.0001	t=40.26, df=3	2	2	0.05	0.0015	t=6.246, df=5	3	3	0.05
BRD1	0.0003	t=9.151, df=5	3	3	0.05	0.0141	t=3.690, df=5	3	3	0.05
SOX4	0.0024	t=5.654, df=5	3	3	0.05	0.0042	t=4.972, df=5	3	3	0.05
SOX11	0.0025	t=5.628, df=5	3	3	0.05	0.0013	t=6.457, df=5	3	3	0.05
KLF12	0.0095	t=4.088, df=5	3	3	0.05	0.0162	t=3.993, df=4	3	2	0.05

Supplementary Table 2. Additional statistical information of western blot analysis in loss-of-function experiments (Fig. 3d)

Gene Target	n
NT1	195
NT2	245
BRD1	295
CBX5	352
CHD3	246
EPC1	291
EPC2	271
EZH2	343
HDAC2	227
KDM1A	146
KDM5B	228
KMT5B	157
MTA2	308
MTF2	191
RBBP4	310
RNF2	121
SMARCA4	238
SMARCE1	258
SMARCAD1	307
RCOR2	408
SOX4	243
SOX11	251
KLF12	205

Comparison	p value
NT2 vs. BRD1	<0.0001
NT2 vs. CBX5	<0.0001
NT2 vs. CHD3	<0.0001
NT2 vs. EPC1	<0.0001
NT2 vs. EPC2	<0.0001
NT2 vs. EZH2	0.0005
NT2 vs. HDAC2	>0.9999
NT2 vs. KDM1A	0.5249
NT2 vs. KDM5B	0.9339
NT2 vs. KMT5B	<0.0001
NT2 vs. MTA2	0.8012
NT2 vs. MTF2	0.0213
NT2 vs. RBBP4	0.9559
NT2 vs. RNF2	<0.0001
NT2 vs. SMARCA4	0.0009
NT2 vs. SMARCE1	0.1523
NT2 vs. SMARCAD1	0.0003
NT2 vs. RCOR2	0.5868
NT2 vs. SOX4	0.9997
NT2 vs. SOX11	>0.9999
NT2 vs. KLF12	0.0101
NT2 vs. NT1	0.9528

Supplementary Table 3. Additional statistical information of Ca^{2+} imaging analysis in loss-of-function experiments (Fig. 3e)

Gene name	Forward	Reverse
**DOT1L (human)**	CTGCCGGTCTACGATAAACATC	AGCTTGAGATCCGGGATTTCT
**EHMT1 (human)**	CATGCAGCCAGTAAAGATCCC	CTGCTGTCGTCCAAAGTCAG
**KDM5B (human)**	AGTGGGCTCACATATCAGAGG	CAAACACCTTAGGCTGTCTCC
**KMT5B (human)**	AGGACAGAGTCGCTATGTACC	CAAACTGGTTGCTAGGTCATCAT
**CHD5 (human)**	GAATGACCCACGGTACATGA	CCCTTGTGGACCTCAGACTT
**JADE2 (human)**	ATCGAGTACGACGAGGATGTT	CACACAGACGTTGCACTTGTC
**Ezh2 (mouse)**	CATACGCTCTTCTGTCGACGATG	ACACTGTGGTCCACAAGGCTTG
**Tbp (mouse)**	CTACCGTGAATCTTGGCTGTAAAC	AATCAACGCAGTTGTCCGTGGC
**Chd5 (mouse)**	TGCAACCATCCGTACCTCTTCC	TCAGCACTCTGTGCCCTTCATC
**Syn1 (mouse)**	TATGCCACTGCTGAGCCCTTCA	ATGGCAATCTGCTCAAGCATAGC
**Fos (mouse)**	GGGAATGGTGAAGACCGTGTCA	GCAGCCATCTTATTCCGTTCCC
**SynI (mouse)**	TATGCCACTGCTGAGCCCTTCA	ATGGCAATCTGCTCAAGCATAGC
**Pgc1a (mouse)**	GAATCAAGCCACTACAGACACCG	CATCCCTCTTGAGCCTTTCGTG

Supplementary Table 4. Sequence of customized qRT-PCR primers.

gRNA ID	gRNA Target sequence
NT1	AAAAAGCTTCCGCCTGATGG
NT2	CATAGGTCCCTAGCAACTCC
BRD1#1	AAATAGGATTGCGAATCAGG
BRD1#2	AAACTGCTTCTTCCGCTGAA
CBX5#1	TACCCAGGGAGCACAATACT
CBX5#2	TAAATTCAGAAATTAGCTCA
CHD3#1	GCAACTCTGCTTCTGACCTG
CHD3#2	GGAGCATGTGTTCTCTGAGG
EPC1#1	TTTAGGAACATCATCTTCAG
EPC1#2	GCGGGCTATTTCAGCACAGC
EPC2#1	TTGACGTTGCTCTCTGCCTC
EPC2#2	TATTACAATCGCTTGTACAA
EZH2#1	CGGAAATCTTAAACCAAGAA
EZH2#2	GCAATGAGCTCACAGAAGTC
HDAC2#1	TGGGTCATGCGGATTCTATG
HDAC2#2	ACAGCAAGTTATGGGTCATG
KDM1A#1	ATACTCATCTTCTGAGAGGT
KDM1A#2	ACCCCCTCAAGCCCCACCTG
KDM5B#1	ACTCCCAGTACTTTGCAATC
KDM5B#2	GCAAAGTACTGGGAGTTACA
KMT5B#1	TTTCACAGGTAATGGTAACT
KMT5B#2	AGTCGCTATGTACCATCCTC
MTA2#1	GCGCCATCAACTGAAGCACC
MTA2#2	GAAGAGGAATCAAAGCAGCC
MTF2#1	TGATTGATTCAGATGAAAAA
MTF2#2	CAGATGAAAAATGGCTCTGT
RBBP4#1	TCATGTCACCTGGTTACATC
RBBP4#2	ATTGCCGTTACAGACCAGAA
RNF2#1	ATCATCACAGCCCTTAGAAG
RNF2#2	AATTCACTGTGTAGACTTCG
SMARCA4#1	ATGGTCCCTCTCGCAGCCCA
SMARCA4#2	CTTTCATCTGGTTGTAGCGC
SMARCE1#1	CAGCAAATGCCCAGCACACC
SMARCE1#2	TCCTACCGTGACCCGGCTGT
SMARCAD1#1	AACTGTATTGGAGCAATTTG
SMARCAD1#2	GAACTGTATTGGAGCAATTT
RCOR2#1	TCTGTGGCATAAGCACGATG
RCOR2#2	GCATTTGCCATGGAAGCCAA
SOX4#1	GCCCGAGTCCGAGCTCTCGC
SOX4#2	ATTGTTGGTTTGCTGCACCA
SOX11#1	GCGGATCATGGTGCAGCAGG
SOX11#2	ATTCATGGCTTGCAGCCCGG
KLF12#1	CGTCCACAACTATCCCGATA
KLF12#2	ACAGATAACGAGTCCTCCGG

Supplementary Table 5. List of gRNA sequences used for gene KO in hPSC-derived neurons.
