# Supplementary material for: An epigenetic barrier sets the timing of human neuronal maturation
Source: Nature. 2024 Jan 31;626(8000):881–90. doi: 10.1038/s41586-023-06984-8 (PMC10881400; doi:10.1038/s41586-023-06984-8)
Supplement: Supplementary file 2 — Reporting Summary [file 41586_2023_6984_MOESM2_ESM.pdf]

nature portfolio

Corresponding author(s): Gabriele Ciceri, Lorenz Studer

Last updated by author(s): Nov 4, 2023

Reporting Summary

Nature Portfolio wishes to improve the reproducibility of the work that we publish. This form provides structure for consistency and transparency in reporting. For further information on Nature Portfolio policies, see our Editorial Policies and the Editorial Policy Checklist.

Statistics

For all statistical analyses, confirm that the following items are present in the figure legend, table legend, main text, or Methods section.

n/a	Confirmed	
☐	☒	The exact sample size (n) for each experimental group/condition, given as a discrete number and unit of measurement
☐	☒	A statement on whether measurements were taken from distinct samples or whether the same sample was measured repeatedly
☐	☒	The statistical test(s) used AND whether they are one- or two-sided<br>*Only common tests should be described solely by name; describe more complex techniques in the Methods section.*
☒	☐	A description of all covariates tested
☐	☒	A description of any assumptions or corrections, such as tests of normality and adjustment for multiple comparisons
☐	☒	A full description of the statistical parameters including central tendency (e.g. means) or other basic estimates (e.g. regression coefficient) AND variation (e.g. standard deviation) or associated estimates of uncertainty (e.g. confidence intervals)
☐	☒	For null hypothesis testing, the test statistic (e.g. F, t, r) with confidence intervals, effect sizes, degrees of freedom and P value noted<br>*Give P values as exact values whenever suitable.*
☒	☐	For Bayesian analysis, information on the choice of priors and Markov chain Monte Carlo settings
☒	☐	For hierarchical and complex designs, identification of the appropriate level for tests and full reporting of outcomes
☐	☒	Estimates of effect sizes (e.g. Cohen's d, Pearson's r), indicating how they were calculated

Our web collection on statistics for biologists contains articles on many of the points above.

Software and code

Policy information about availability of computer code

Data collection	LAS X 3.5.5.19976, ZEN Blue 2.3, ZEN Blue 3.1, OLYMPUS CellSens Dimension 1.15, SlideBook6.0.7, Harmony v4.1 (for PerkinElmer Operetta High content imaging system) were used for microscopy: Image Lab (Bio-Rad) version 6.1.0 were used for acquisition of Western Blot chemiluminescence images. Clampex v10.2 and Clampex v10.6 were used for acquisition of electrophysiological data.
Data analysis	Harmony v4.1 (for PerkinElmer Operetta High content imaging system), Imaris v9.9.1 and ImarisViewer v9.9.1, FIJI (ImageJ2) v2.9.0/1.53t were used for image analysis. MATLAB (Mathworks) R2020b and R2021b (with published scripts from Sun & Sudhof. J neurosci Methods, 2021) were using for Calcium Imaging analysis. Image Lab (Bio-Rad) v6.1.0 was used for densitometric analysis of Western Blot analysis. Synaptosoft MiniAnalysis v6 and, Clampfit 10.2 and Clampfit 10.6 were used for analysis of electrophysiological recordings. Integrative Genomics Viewer (IGV) v2.8.9 was used for viewing epigenomic tracks. DAVID v6.8 for gene ontology analysis.<br>FastQC v 0.11.3 and v 0.11.5, STAR v 2.5.0 and v 2.7.10b, HTSeq v 0.7.1, DESeq2 v 1.16 and v 1.22.2, Trimmomatic v 0.36, Macs2 v 2.1.0, FIMO v 4.11, TrimGalore v 0.4.5, cutadapt v 1.15, bowtie2 v 2.3.4.1, MarkDuplicates of Picard Tools v 2.16.0, BEDTools suite v 2.29.2, featureCounts v 1.6.1, Homer v4.11 and v 4.6, CellRanger pipeline v6.1.2 , Seurat v4.2.0, URD v1.1.1 were used for bioinfomatic analyses of RNAseq, ATACseq and Cut&Run data. GraphPad Prism 8, 9 and 10 and R v 3.5.2 and v4.1 were used for statistical computing and graphing.

For manuscripts utilizing custom algorithms or software that are central to the research but not yet described in published literature, software must be made available to editors and reviewers. We strongly encourage code deposition in a community repository (e.g. GitHub). See the Nature Portfolio guidelines for submitting code & software for further information.

Data

Policy information about availability of data

All manuscripts must include a data availability statement. This statement should provide the following information, where applicable:

- Accession codes, unique identifiers, or web links for publicly available datasets
- A description of any restrictions on data availability
- For clinical datasets or third party data, please ensure that the statement adheres to our policy

All genomic datasets have been deposited at GEO GSE196075, GSE196109 and GSE226223. Publicly available datasets of human brain development were from BrainSpan atlas of the developing human brain (https://www.brainspan.org/static/download.html). Genome assembly GRCh38 (hg19) (https://www.ncbi.nlm.nih.gov/datasets/genome/GCF_000001405.13/). GRCh38 genome assembly (hg38) (https://www.ncbi.nlm.nih.gov/datasets/genome/GCF_000001405.26/). Published dataset of mouse cortical development were from Di Bella et al., Nature 2021; PMID: 34163074). Published dataset of hPSC cortical differentiations were from Yao et al., Cell Stem Cell 2017, PMID: 28094016 and Volpato et al. Stem Cell Reports 2018, PMID: 30245212

Field-specific reporting

Please select the one below that is the best fit for your research. If you are not sure, read the appropriate sections before making your selection.

☒ Life sciences ☐ Behavioural & social sciences ☐ Ecological, evolutionary & environmental sciences

For a reference copy of the document with all sections, see nature.com/documents/nr-reporting-summary-flat.pdf

Life sciences study design

All studies must disclose on these points even when the disclosure is negative.

Sample size	No statistical methods were used to predetermine sample sizes. Sample sizes were estimated based on previous experience and previous publications in the field. (Qi et al., Nat Biotechnol 2017; Tchieu et al., Nat Biotechnol 2017).
Data exclusions	No samples were excluded. However, wells with technical failure (extensive monolayer pealing or non-neural contaminating cell populations) were excluded from analysis.
Replication	Number of replicates is specified for each experiment. All experiments were performed in at least 2 independent biological replicates. Experiments which failed to produce pure neuronal populations, or experiments where cells detached from plate surface were not used as replicates (see above).
Randomization	samples were randomly assigned into experimental group. For KO studies and small molecule treatment , samples were de-identified respect to the molecular target and a code was assigned at each condition.
Blinding	Investigators were not blinded because the investigator was aware of days of differentiations and experimental conditions while setting up the experiment. Data collection and analysis in KO studies and small molecule treatment were conducted using de-identified samples respect to the molecular target. Transcriptional and genomic assays were performed with the same bioinformatic pipelines between conditions.

Reporting for specific materials, systems and methods

We require information from authors about some types of materials, experimental systems and methods used in many studies. Here, indicate whether each material, system or method listed is relevant to your study. If you are not sure if a list item applies to your research, read the appropriate section before selecting a response.

Materials & experimental systems

n/a	Involved in the study	
☐	☒	Antibodies
☐	☒	Eukaryotic cell lines
☒	☐	Palaeontology and archaeology
☒	☐	Animals and other organisms
☒	☐	Human research participants
☒	☐	Clinical data
☒	☐	Dual use research of concern

Methods

n/a	Involved in the study	
☐	☒	ChIP-seq
☒	☐	Flow cytometry
☒	☐	MRI-based neuroimaging

Antibodies

Antibodies used	Primary antibodies:<br>rabbit anti-Pax6 (901301, Biolegend)

rabbit anti-FoxG1 (bf1) (M227, Clonetech)
mouse anti-Nestin (M015012, Neuromics)
mouse anti MAP2 (M1406, Sigma)
chicken anti-MAP2 (ab5392, Abcam)
rabbit anti-Class III beta-tubulin TUJI (MRB-435P, Covance)
mouse anti-Ki67 (M7240, Dako)
rabbit anti-Ki67 (RM-9106, Thermo Scientific)
rabbit anti-Tbr1 (ab183032, Abcam)
rat anti-Ctip2 (ab18465, Abcam)
mouse anti-Satb2 (ab51502, Abcam)
rabbit anti-Synapsin I (S193, Sigma)
mouse anti-Neurofilament H (non phosphorylated) (SMI32; Enzo Life science)
mouse anti c-Fos (ab208942, Abcam)
mouse anti-HLA Class I ABC (ab70328, abcam)
goat anti-RFP (200-101-379, Rockland)
rabbit anti-DsRed (632496, Clontech)
mouse anti-Syntaxin 1A (110 111; SYSY)
mouse anti-actin (MAB1501; Millipore)
mouse anti-Cas9 (14697; Cell Signaling Technology)
rabbit anti-Chd3 (ab109195, Abcam)
rabbit anti-KDM5B (ab181089, abcam)
rabbit anti-H3K4me3 (ab8580, abcam)
rabbit anti-H3K9me3 (ab8898, abcam)
rabbit anti-H3K27me3 (9733, Cell Signaling Technologies)
rabbit anti-H3K27ac (39034, Active Motif)
mouse anti-PSD95 (MA1-046, Thermo)
rabbit anti-GFAP (Z033429-2, Dako)
chicken anti-GFP (ab13970, Abcam)
rat anti-SOX2 (14-9811-82, Thermo)
rabbit anti-AQP4 (HPA014784, SIGMA)
sheep anti-EOMES (AF6166, R&D)
rabbit anti-Tbr1 1:500 (ab31940, Abcam)
Normal rabbit IgG (2729, Cell Signaling Technologies)
Secondary antibodies:
anti-mouse IgG HRP-linked (7076; Cell Signaling Technology)
anti-rabbit IgG HRP-linked (7074; Cell Signaling Technology)
donkey anti-mouse Alexa Fluor 488 (A21202, ThermoFisher Scientific)
donkey anti-rabbit Alexa Fluor 488 (A21206, ThermoFisher Scientific)
donkey anti-rat Alexa Fluor 488 (A21208, ThermoFisher Scientific)
donkey anti-sheep Alexa Fluor 488 (A11015, ThermoFisher Scientific)
goat anti-chicken Alexa Fluor 488 (A11038, ThermoFisher Scientific)
donkey anti-mouse Alexa Fluor 555 (A31570, ThermoFisher Scientific)
donkey anti-rabbit Alexa Fluor 555 (A31572, ThermoFisher Scientific)
donkey anti-goat Alexa Fluor 555 (A21432, ThermoFisher Scientific)
goat anti-rabbit Alexa Fluor 555 (A27039, ThermoFisher Scientific)
donkey anti-mouse Alexa Fluor 647 (A31571, ThermoFisher Scientific)
donkey anti-rabbit Alexa Fluor 647 (A31573, ThermoFisher Scientific)
goat anti-rat Alexa Fluor 647 (A21247, ThermoFisher Scientific)
goat anti-chicken Alexa Fluor 647 (A21449, ThermoFisher Scientific)

Validation

rabbit anti-Pax6 (901301, Biolegend), previously Covance Catalog# PRB-278P: Verified reactivity in whole cell extract from HEK293T cells and mouse brain tissue by manufacturer. It appears in 308 citations according to manufacturer website
rabbit anti-FoxG1 (Bf1) (M227, Clonetech): Recognizes C-terminal region of human and mouse Brain factor 1 and specifically reacts with human and mouse Brain factor 1 and validated in rodent tissue by manufacturer. It appears in at least 4 references, according to Takara Website.
mouse anti-Nestin (M015012, Neuromics): generated with human Nestin fragment with reactivity towards Human, Primate and validated by Immunocytochemistry also in neural rosette from differentiation of hPSC by manufacturer. It appears in at least 3 citations according to manufacturer website.
mouse anti MAP2 (M1406, Sigma): Validated for immunocytochemistry and immunofluorescence by manufacturer and it appears in at least 4 citations according to manufacturer website and 321 citations in CiteAb website, which include use iPSC-derived neurons.
chicken anti-MAP2 (ab5392, Abcam): generated with human antigen. Validated for ICC and WB in mouse and rat brain tissue by manufacturer. Widely used in immunofluorescence, cited in 681 references on manufacturer website and 917 citations, including 120 with human reactivity on Citeab website.
rabbit anti-Class III beta-tubulin TUJI (MRB-435P, Covance): highly reactive to neuron specific Class III beta-tubulin (TUJI) and does not identify beta-tubulin found in glial cells and validated for IHC, WB, IF by manufacturer. In appears in 310 publications according to CiteAb website.
mouse anti-Ki67 (M7240, Dako): generated with Human recombinant peptide, validated for WB and IHC by manufacturer and has more than 4000 literature citations according to manufacturer website
rabbit anti-Ki67 (RM-9106, Thermo Scientific): validated for Human reactivity and immunohistology according to manufacturer website
rabbit anti-Tbr1 (ab183032, Abcam): validated for ICC and WB in rodent brain, as well as human cerebral organoid tissue and in human brain lysate, by manufacturer. Widely used for detection of TBR-1 by immunofluorescence. It appears in 14 references on the manufacturer website and 30 citations according to Citeab website.
rat anti-Ctip2 (ab18465, Abcam): Widely used for CTIP2 detection in mouse tissue. Validated for human reactivity and immunocytochemistry/immunofluorescence by manufacturer. It appears in 718 references on the manufacturer website

mouse anti-Satb2 (ab51502, Abcam): Widely used for Satb2 detection in mouse tissue. Validated for human reactivity and immunocytochemistry/immunofluorescence and Knock-out validate by manufacturer. Appears in 269 references on the manufacturer website
rabbit anti-Synapsin I (S193, Sigma): Validated for human reactivity and ICC/IHC by manufacturer. It appears in 40 references on the manufacturer website
mouse anti-Neurofilament H (non phosphorylated) (SMI32; Enzo Life science): Validated for mammalian reactivity and ICC and WB applications by manufacturer. Clone validated in 140 publications from Biolegend website.
mouse anti c-Fos (ab208942, Abcam): Knock out validated and for ICC/IF in human tissue and for mouse and human reactivity and in FOS cell knockout line by manufacturer. A widely used antibody for immunofluorescence, it appears in 75 citations on manufacturers website and 127 citations of Citeab website.
mouse anti-HLA Class I ABC (ab70328, abcam): validated for human reactivity in IHC and WB by manufacturer and it appears in 141 citations on manufacturers website and 157 reference in CiteAb webstite.
goat anti-RFP (200-101-379, Rockland): Validate for WB, IHC, IF by manufacturer and it appears on 26 references on manufacturers website and 36 references in CiteAb webstite.
rabbit anti-DsRed (632496, Clontech): Validated for IHC, ICC, WB applications with 1257 references according to CiteAb website.
mouse anti-Syntaxin 1A (110 111; SYSY): Knockout validated and validate for WB, IHC, ICC and reactivity with human by manufacturer. it appears in 37 citations on manufacturers website
mouse anti-actin (MAB1501; Millipore): highly published monoclonal antibody validated for WB and to date for reactivity for all animal species and cell types with an actin form by manufacturer. It appears in 2886 citations according to CiteAb website
mouse anti-Cas9 (14697; Cell Signaling Technology): Validate for WB, IHC, IF applications by manufacturer and it appears in 135 citations according to manufacture website
rabbit anti-Chd3 (ab109195, Abcam): Knockout validated and validated reactivity with Rat and Human and suitable for WB, IHC-P, ICC/IF by manufacturer. It appears in 3 citations according to manufacturer website
rabbit anti-KDM5B (ab181089, abcam): Knockout validated and validated reactivity with Mouse, Rat, Human and for WB by manufacturer. It appears in 14 citations according to manufacturer website and 22 citations according to CiteAb webstite
rabbit anti-H3K4me3 (ab8580, abcam): ChIP Grade, suitable for ChIP, WB, IHC-P, ICC/IF and reactivity with human by manufacturer. It appears in 1982 citations according to manufacturer website and 2242 citations according to CiteAb webstite
rabbit anti-H3K9me3 (ab8898, abcam): ChIP Grade, validated for WB, IHC-P, ChIP, ICC/IF and reactivity with Mouse and Human by manufacturer. It appears in 1603 citations according to manufacturer website and 1885 citations according to CiteAb website
rabbit anti-H3K27me3 (9733, Cell Signaling Technologies): Validate for WB, IHC, IF, Chip, C&R and reactivity validated in human and mouse and it appears in 1511 citations according to manufacturer website and in1228 citations according to CiteAb website
rabbit anti-H3K27ac (39034, Active Motif): validate for human reactivity, ChIP, ChIP-Seq, ICC/IF, WB, CUT&Tag, CUT&RUN and it appears in 3 references according to manufacturer website and 21 citations according to CiteAb website
mouse anti-PSD95 (MA1-046, Thermo): Knockout validated and validated for human reactivity for applications including ICC/IF by manufacturer. It appears in 230 references according to the manufacturer website.
rabbit anti-GFAP (Z033429-2, Dako): Validated for IHC with 3433 citations according to CiteAb webstite. This includes Tchieu et al. Nat Biotech 2019 on hPSC-derived astrocytes and Fair at al., Stem Cell Report 2020 iPSC-derived cortical organoids among others.
chicken anti-GFP (ab13970, Abcam): Validated for WB, ICC/IF by manufacturer, it appears in 3182 references according to manufacturer website
rat anti-SOX2 (14-9811-82, Thermo): Verified by knockdown and human reactivity for applications including IHC by the manufacturer. It appears in 60 references according to manufacturer website and 80 references according to CiteAb website
rabbit anti-AQP4 (HPA014784, SIGMA): Validated for human and mouse reactivity by manufacturer. It appears in 35 references according to manufacturer website and 39 citations according to CiteAb website
sheep anti-EOMES (AF6166, R&D): Validated for human reactivity by manufacturer, it appears in 9 references according to manufacturers and 31 citations according to CiteAb website. Validated for ICC and IHC applications, including published reports on iPSC -derived brain organoids.
rabbit anti-Tbr1 1:500 (ab31940, Abcam): validated for human reactivity by manufacture and widely used ICC in mouse brain sections and human primary tissue and in iPSC-derived organoids. It appears in 425 references on the manufacturer website.
Normal rabbit IgG (2729, Cell Signaling Technologies): IP, ChiP It appears in 2053 references according to manufacturer website and 2160 citations according to CiteAb website.

Eukaryotic cell lines

Policy information about cell lines

Cell line source(s)	H9 (WA-09): WiCell Stemcell Bank; CRISPR edited GPI::Cas9 knock-in hPSC line was generated in the Studer lab by targeting of H9 hPSC; H1 (WA-01): WiCell Stemcell Bank; MSK-SRF001: Memorial Sloan Kettering Cancer Center; HEK293T: ATCC CRL-3216, mEpiSC B6.129_4: Memorial Sloan Kettering Cancer Center/ Vierbuchen lab (Medina-Cano et al., Dev 2022)
Authentication	hPSC were authenticated by STR, GPI::Cas9 knock-in hPSC line was validated by genomic PCR and Cas9 mRNA and protein expression by qRT-PCR and Western Blot respectively and screened for Karyotype banding, HEK293T authentication from source, no additional STR was performed on mEPISC
Mycoplasma contamination	All cell lines were regularly tested negative for mycoplasma contamination.
Commonly misidentified lines (See ICLAC register)	No commonly misidentified lines were used in this study.

ChIP-seq

Data deposition

☒ Confirm that both raw and final processed data have been deposited in a public database such as GEO.

☒ Confirm that you have deposited or provided access to graph files (e.g. BED files) for the called peaks.

Data access links<br>*May remain private before publication.*	https://www.ncbi.nlm.nih.gov/geo/query/acc.cgi?acc=GSE196109
Files in database submission	Processed data:<br>CnR_H3K27ac_D53_rep1.hg19.sorted.RmDup.10mNorm.bw<br>CnR_H3K27ac_D53_rep2.hg19.sorted.RmDup.10mNorm.bw<br>CnR_H3K27ac_NPC_rep1.hg19.sorted.RmDup.10mNorm.bw<br>CnR_H3K27ac_NPC_rep2.hg19.sorted.RmDup.10mNorm.bw<br>CnR_H3K27me3_D53_rep1.hg19.sorted.RmDup.10mNorm.bw<br>CnR_H3K27me3_D53_rep2.hg19.sorted.RmDup.10mNorm.bw<br>CnR_H3K27me3_NPC_rep1.hg19.sorted.RmDup.10mNorm.bw<br>CnR_H3K27me3_NPC_rep2.hg19.sorted.RmDup.10mNorm.bw<br>CnR_H3K4me3_D53_rep1.hg19.sorted.RmDup.10mNorm.bw<br>CnR_H3K4me3_D53_rep2.hg19.sorted.RmDup.10mNorm.bw<br>CnR_H3K4me3_NPC_rep1.hg19.sorted.RmDup.10mNorm.bw<br>CnR_H3K4me3_NPC_rep2.hg19.sorted.RmDup.10mNorm.bw<br>CnR_H3K9me3_D53_rep1.hg19.sorted.RmDup.10mNorm.bw<br>CnR_H3K9me3_D53_rep2.hg19.sorted.RmDup.10mNorm.bw<br>CnR_H3K9me3_NPC_rep1.hg19.sorted.RmDup.10mNorm.bw<br>CnR_H3K9me3_NPC_rep2.hg19.sorted.RmDup.10mNorm.bw<br>CnR_IgG_D53_rep1.hg19.sorted.RmDup.10mNorm.bw<br>CnR_IgG_D53_rep2.hg19.sorted.RmDup.10mNorm.bw<br>CnR_IgG_NPC_rep1.hg19.sorted.RmDup.10mNorm.bw<br>CnR_IgG_NPC_rep2.hg19.sorted.RmDup.10mNorm.bw<br><br>CnR_DMSO_H3K27me3_rep1.hg19.sorted.RmDup.10mNorm.bw<br>CnR_DMSO_H3K27me3_rep2.hg19.sorted.RmDup.10mNorm.bw<br>CnR_DMSO_H3K4me3_rep1.hg19.sorted.RmDup.10mNorm.bw<br>CnR_DMSO_H3K4me3_rep2.hg19.sorted.RmDup.10mNorm.bw<br>CnR_DOT1Linh_H3K27me3_rep1.hg19.sorted.RmDup.10mNorm.bw<br>CnR_DOT1Linh_H3K27me3_rep2.hg19.sorted.RmDup.10mNorm.bw<br>CnR_DOT1Linh_H3K4me3_rep1.hg19.sorted.RmDup.10mNorm.bw<br>CnR_DOT1Linh_H3K4me3_rep2.hg19.sorted.RmDup.10mNorm.bw<br>CnR_EHMTinh_H3K27me3_rep1.hg19.sorted.RmDup.10mNorm.bw<br>CnR_EHMTinh_H3K27me3_rep2.hg19.sorted.RmDup.10mNorm.bw<br>CnR_EHMTinh_H3K4me3_rep1.hg19.sorted.RmDup.10mNorm.bw<br>CnR_EHMTinh_H3K4me3_rep2.hg19.sorted.RmDup.10mNorm.bw<br>CnR_EZH2inh_H3K27me3_rep1.hg19.sorted.RmDup.10mNorm.bw<br>CnR_EZH2inh_H3K27me3_rep2.hg19.sorted.RmDup.10mNorm.bw<br>CnR_EZH2inh_H3K4me3_rep1.hg19.sorted.RmDup.10mNorm.bw<br>CnR_EZH2inh_H3K4me3_rep2.hg19.sorted.RmDup.10mNorm.bw<br>CnR_DMSO_noab_rep1.hg19.sorted.RmDup.10mNorm.bw<br>CnR_DOT1Linh_noab_rep1.hg19.sorted.RmDup.10mNorm.bw<br>CnR_DOT1Linh_noab_rep2.hg19.sorted.RmDup.10mNorm.bw<br>CnR_EHMTinh_noab_rep1.hg19.sorted.RmDup.10mNorm.bw<br>CnR_EHMTinh_noab_rep2.hg19.sorted.RmDup.10mNorm.bw<br>CnR_EZH2inh_noab_rep1.hg19.sorted.RmDup.10mNorm.bw<br>CnR_EZH2inh_noab_rep2.hg19.sorted.RmDup.10mNorm.bw<br><br>Raw data:<br>CnR_H3K27ac_D53_rep1_IGO_11870_B_16_S152_L003_R1_001.fastq.gz<br>CnR_H3K27ac_D53_rep1_IGO_11870_B_16_S152_L003_R2_001.fastq.gz<br>CnR_H3K27ac_D53_rep2_IGO_11870_B_34_S153_L003_R1_001.fastq.gz<br>CnR_H3K27ac_D53_rep2_IGO_11870_B_34_S153_L003_R2_001.fastq.gz<br>CnR_H3K27ac_NPC_rep1_IGO_11870C_concat_R1_001.fastq.gz<br>CnR_H3K27ac_NPC_rep1_IGO_11870C_concat_R2_001.fastq.gz<br>CnR_H3K27ac_NPC_rep2_IGO_11870C_concat_R1_001.fastq.gz<br>CnR_H3K27ac_NPC_rep2_IGO_11870C_concat_R2_001.fastq.gz<br>CnR_H3K27me3_D53_rep1_IGO_11870_B_15_S154_L003_R1_001.fastq.gz<br>CnR_H3K27me3_D53_rep1_IGO_11870_B_15_S154_L003_R2_001.fastq.gz<br>CnR_H3K27me3_D53_rep2_IGO_11870_B_33_S155_L003_R1_001.fastq.gz<br>CnR_H3K27me3_D53_rep2_IGO_11870_B_33_S155_L003_R2_001.fastq.gz<br>CnR_H3K27me3_NPC_rep1_IGO_11870C_concat_R1_001.fastq.gz<br>CnR_H3K27me3_NPC_rep1_IGO_11870C_concat_R2_001.fastq.gz<br>CnR_H3K27me3_NPC_rep2_IGO_11870C_concat_R1_001.fastq.gz<br>CnR_H3K27me3_NPC_rep2_IGO_11870C_concat_R2_001.fastq.gz

CnR_H3K4me3_D53_rep1_IGO_11870_B_13_S156_L003_R1_001.fastq.gz
CnR_H3K4me3_D53_rep1_IGO_11870_B_13_S156_L003_R2_001.fastq.gz
CnR_H3K4me3_D53_rep2_IGO_11870_B_31_S157_L003_R1_001.fastq.gz
CnR_H3K4me3_D53_rep2_IGO_11870_B_31_S157_L003_R2_001.fastq.gz
CnR_H3K4me3_NPC_rep1_IGO_11870C_concat_R1_001.fastq.gz
CnR_H3K4me3_NPC_rep1_IGO_11870C_concat_R2_001.fastq.gz
CnR_H3K4me3_NPC_rep2_IGO_11870C_concat_R1_001.fastq.gz
CnR_H3K4me3_NPC_rep2_IGO_11870C_concat_R2_001.fastq.gz
CnR_H3K9me3_D53_rep1_IGO_11870_B_14_S158_L003_R1_001.fastq.gz
CnR_H3K9me3_D53_rep1_IGO_11870_B_14_S158_L003_R2_001.fastq.gz
CnR_H3K9me3_D53_rep2_IGO_11870_B_32_S159_L003_R1_001.fastq.gz
CnR_H3K9me3_D53_rep2_IGO_11870_B_32_S159_L003_R2_001.fastq.gz
CnR_H3K9me3_NPC_rep1_IGO_11870C_concat_R1_001.fastq.gz
CnR_H3K9me3_NPC_rep1_IGO_11870C_concat_R2_001.fastq.gz
CnR_H3K9me3_NPC_rep2_IGO_11870C_concat_R1_001.fastq.gz
CnR_H3K9me3_NPC_rep2_IGO_11870C_concat_R2_001.fastq.gz
CnR_IgG_D53_rep1_IGO_11870_B_17_S162_L003_R1_001.fastq.gz
CnR_IgG_D53_rep1_IGO_11870_B_17_S162_L003_R2_001.fastq.gz
CnR_IgG_D53_rep2_IGO_11870_B_35_S163_L003_R1_001.fastq.gz
CnR_IgG_D53_rep2_IGO_11870_B_35_S163_L003_R2_001.fastq.gz
CnR_IgG_NPC_rep1_IGO_11870C_concat_R1_001.fastq.gz
CnR_IgG_NPC_rep1_IGO_11870C_concat_R2_001.fastq.gz
CnR_IgG_NPC_rep2_IGO_11870C_concat_R1_001.fastq.gz
CnR_IgG_NPC_rep2_IGO_11870C_concat_R2_001.fastq.gz

CnR_DMSO_H3K27me3_rep1_IGO_11870_D_1_S64_L002_R1_001.fastq.gz
CnR_DMSO_H3K27me3_rep1_IGO_11870_D_1_S64_L002_R2_001.fastq.gz
CnR_DMSO_H3K27me3_rep2_IGO_11870_D_21_S66_L002_R1_001.fastq.gz
CnR_DMSO_H3K27me3_rep2_IGO_11870_D_21_S66_L002_R2_001.fastq.gz
CnR_DMSO_H3K4me3_rep1_IGO_11870_D_rep2_S75_L002_R1_001.fastq.gz
CnR_DMSO_H3K4me3_rep1_IGO_11870_D_rep2_S75_L002_R2_001.fastq.gz
CnR_DMSO_H3K4me3_rep2_IGO_11870_D_22_S67_L002_R1_001.fastq.gz
CnR_DMSO_H3K4me3_rep2_IGO_11870_D_22_S67_L002_R2_001.fastq.gz
CnR_DOT1Linh_H3K27me3_rep1_IGO_11870_D_16_S60_L002_R1_001.fastq.gz
CnR_DOT1Linh_H3K27me3_rep1_IGO_11870_D_16_S60_L002_R2_001.fastq.gz
CnR_DOT1Linh_H3K27me3_rep2_IGO_11870_D_36_S82_L002_R1_001.fastq.gz
CnR_DOT1Linh_H3K27me3_rep2_IGO_11870_D_36_S82_L002_R2_001.fastq.gz
CnR_DOT1Linh_H3K4me3_rep1_IGO_11870_D_17_S61_L002_R1_001.fastq.gz
CnR_DOT1Linh_H3K4me3_rep1_IGO_11870_D_17_S61_L002_R2_001.fastq.gz
CnR_DOT1Linh_H3K4me3_rep2_IGO_11870_D_37_S83_L002_R1_001.fastq.gz
CnR_DOT1Linh_H3K4me3_rep2_IGO_11870_D_37_S83_L002_R2_001.fastq.gz
CnR_EHMTinh_H3K27me3_rep1_IGO_11870_D_11_S55_L002_R1_001.fastq.gz
CnR_EHMTinh_H3K27me3_rep1_IGO_11870_D_11_S55_L002_R2_001.fastq.gz
CnR_EHMTinh_H3K27me3_rep2_IGO_11870_D_31_S77_L002_R1_001.fastq.gz
CnR_EHMTinh_H3K27me3_rep2_IGO_11870_D_31_S77_L002_R2_001.fastq.gz
CnR_EHMTinh_H3K4me3_rep1_IGO_11870_D_12_S56_L002_R1_001.fastq.gz
CnR_EHMTinh_H3K4me3_rep1_IGO_11870_D_12_S56_L002_R2_001.fastq.gz
CnR_EHMTinh_H3K4me3_rep2_IGO_11870_D_32_S78_L002_R1_001.fastq.gz
CnR_EHMTinh_H3K4me3_rep2_IGO_11870_D_32_S78_L002_R2_001.fastq.gz
CnR_EZH2inh_H3K27me3_rep1_IGO_11870_D_6_S90_L002_R1_001.fastq.gz
CnR_EZH2inh_H3K27me3_rep1_IGO_11870_D_6_S90_L002_R2_001.fastq.gz
CnR_EZH2inh_H3K27me3_rep2_IGO_11870_D_26_S71_L002_R1_001.fastq.gz
CnR_EZH2inh_H3K27me3_rep2_IGO_11870_D_26_S71_L002_R2_001.fastq.gz
CnR_EZH2inh_H3K4me3_rep1_IGO_11870_D_7_S91_L002_R1_001.fastq.gz
CnR_EZH2inh_H3K4me3_rep1_IGO_11870_D_7_S91_L002_R2_001.fastq.gz
CnR_EZH2inh_H3K4me3_rep2_IGO_11870_D_27_S72_L002_R1_001.fastq.gz
CnR_EZH2inh_H3K4me3_rep2_IGO_11870_D_27_S72_L002_R2_001.fastq.gz
CnR_DMSO_noab_rep1_IGO_11870_D_5_S89_L002_R1_001.fastq.gz
CnR_DMSO_noab_rep1_IGO_11870_D_5_S89_L002_R2_001.fastq.gz
CnR_DOT1Linh_noab_rep1_IGO_11870_D_20_S65_L002_R1_001.fastq.gz
CnR_DOT1Linh_noab_rep1_IGO_11870_D_20_S65_L002_R2_001.fastq.gz
CnR_DOT1Linh_noab_rep2_IGO_11870_D_40_S87_L002_R1_001.fastq.gz
CnR_DOT1Linh_noab_rep2_IGO_11870_D_40_S87_L002_R2_001.fastq.gz
CnR_EHMTinh_noab_rep1_IGO_11870_D_15_S59_L002_R1_001.fastq.gz
CnR_EHMTinh_noab_rep1_IGO_11870_D_15_S59_L002_R2_001.fastq.gz
CnR_EHMTinh_noab_rep2_IGO_11870_D_35_S81_L002_R1_001.fastq.gz
CnR_EHMTinh_noab_rep2_IGO_11870_D_35_S81_L002_R2_001.fastq.gz
CnR_EZH2inh_noab_rep1_IGO_11870_D_10_S54_L002_R1_001.fastq.gz
CnR_EZH2inh_noab_rep1_IGO_11870_D_10_S54_L002_R2_001.fastq.gz
CnR_EZH2inh_noab_rep2_IGO_11870_D_30_S76_L002_R1_001.fastq.gz
CnR_EZH2inh_noab_rep2_IGO_11870_D_30_S76_L002_R2_001.fastq.gz

Genome browser session
(e.g. UCSC)

Processed data can be downloaded and viewed in IGV or another browser.

Methodology

Replicates	2 independent biological replicates per time point and antibody
Sequencing depth	Each sample was sequenced using paired-end 100bp reads and sequencing depth varied between 29.8 and 58.4 million reads with an average alignment rate of 88% and average unique alignment of 63%.
Antibodies	rabbit anti-H3K4me3 (ab8580, abcam), rabbit anti-H3K9me3 (ab8898, abcam); rabbit anti-H3K27me3 (9733, Cell Signaling Technologies); rabbit anti-H3K27ac (309034, Active Motif); Normal rabbit IgG (2729, Cell Signaling Technologies)
Peak calling parameters	Reads were aligned to human assembly hg19 with version 2.3.4.1 of bowtie2 and peaks were called using MACS2 with a p-value setting of 0.001 and scored against a matched IgG control.
Data quality	Metrics to evaluate data quality included signal-to-noise ratio (peak versus flanking regions), total number of peaks called versus matched IgG background and the fold change over background signal, and visual inspection of repressed and active (e.g. housekeeping gene) regions using normalized bigwig files and a genome browser.
Software	Trimgalore version 0.4.5, FastQC version 0.11.5 , cutadapt version 1.15 , Bowtie2 version 2.3.4.1 , MarkDuplicates of Picard Tools version 2.16.0 , featureCounts version 1.6.1 , DESeq2 version 1.22.2, BEDTools suite version 2.29.2 and Homer version 4.11 were used for the analysis of these CUT&RUN data. No custom code was written for the analyses.
